# Fatigue following Acute Q-Fever: A Systematic Literature Review

**DOI:** 10.1371/journal.pone.0155884

**Published:** 2016-05-25

**Authors:** Gabriella Morroy, Stephan P. Keijmel, Corine E. Delsing, Gijs Bleijenberg, Miranda Langendam, Aura Timen, Chantal P. Bleeker-Rovers

**Affiliations:** 1 Department of Infectious Diseases, Municipal Health Service Hart voor Brabant, ‘s-Hertogenbosch, the Netherlands; 2 Radboud Expertise Centre for Q fever, Department of Internal Medicine, Division of Infectious Diseases, Radboud university medical center, Nijmegen, the Netherlands; 3 Department of Internal Medicine, Division of Infectious Diseases, Radboud university medical center, Nijmegen, the Netherlands; 4 Expert Centre for Chronic Fatigue, Radboud university medical center, Nijmegen, the Netherlands; 5 Department of Internal Medicine, Medisch Spectrum Twente, Enschede, the Netherlands; 6 Department of Clinical Epidemiology, Biostatistics and Bioinformatics, Academic Medical Centre, Amsterdam, the Netherlands; 7 Centre for Infectious Disease Control, National Institute for Public Health and the Environment, Bilthoven, the Netherlands; Texas A&M Health Science Center, UNITED STATES

## Abstract

**Background:**

Long-term fatigue with detrimental effects on daily functioning often occurs following acute Q-fever. Following the 2007–2010 Q-fever outbreak in the Netherlands with over 4000 notified cases, the emphasis on long-term consequences of Q-fever increased. The aim of this study was to provide an overview of all relevant available literature, and to identify knowledge gaps regarding the definition, diagnosis, background, description, aetiology, prevention, therapy, and prognosis, of fatigue following acute Q-fever.

**Design:**

A systematic review was conducted through searching Pubmed, Embase, and PsycInfo for relevant literature up to 26^th^ May 2015. References of included articles were hand searched for additional documents, and included articles were quality assessed.

**Results:**

Fifty-seven articles were included and four documents classified as grey literature. The quality of most studies was low. The studies suggest that although most patients recover from fatigue within 6–12 months after acute Q-fever, approximately 20% remain chronically fatigued. Several names are used indicating fatigue following acute Q-fever, of which Q-fever fatigue syndrome (QFS) is most customary. Although QFS is described to occur frequently in many countries, a uniform definition is lacking. The studies report major health and work-related consequences, and is frequently accompanied by nonspecific complaints. There is no consensus with regard to aetiology, prevention, treatment, and prognosis.

**Conclusions:**

Long-term fatigue following acute Q-fever, generally referred to as QFS, has major health-related consequences. However, information on aetiology, prevention, treatment, and prognosis of QFS is underrepresented in the international literature. In order to facilitate comparison of findings, and as platform for future studies, a uniform definition and diagnostic work-up and uniform measurement tools for QFS are proposed.

## Introduction

Q-fever, caused by the Gram-negative intracellular coccobacillus *Coxiella burnetii*, is a zoonosis that occurs worldwide [1]. Between 2007 and 2010 the largest Q-fever outbreak ever described in the literature occurred in the Netherlands, resulting in 4107 notifications [2].

Fatigue following acute Q-fever, also referred to as Q-fever fatigue syndrome (QFS), has been described worldwide in up to 20%-30% of patients [3–8] and may last up to ten years or longer [7, 9]. Although some debated the term QFS [10], it has been frequently used throughout literature. QFS patients experience an impaired health status, pulmonary disorders, and impairment of general and social functioning [3, 7–9, 11, 12], and QFS accounted for major Q-fever-related economic cost during the Dutch outbreak [13]. Therefore, although not always recognised as a (diagnostic) problem, this sequel has major implications. The word “syndrome” refers to other frequently accompanying nonspecific symptoms [3, 8, 9, 14] resembling chronic fatigue syndrome (CFS) [15, 16]. However, in CFS the cause is usually unknown, while in QFS a *C*. *burnetii* infection can be identified as the trigger. Furthermore, QFS has a sudden onset of fatigue, while in CFS this is often not the case. Several queries regarding QFS without clear answers exist. A uniform international definition is not available, and tools to assess this syndrome and its consequences vary [5, 6, 17]. Hypotheses on aetiology appear contradictory [18], and vary from altered cytokine production [6, 19], development of symptoms determined by host and genetic factors [19–21], to the perpetuation of symptoms due to psychogenic factors and behaviour [8]. Furthermore, opinions on possible treatment of QFS differ [5, 6, 17], and questions exist regarding prevention and prognosis.

The aim of this first systematic review regarding fatigue after acute Q-fever in humans is to provide an overview of all relevant available literature, and to identify knowledge gaps regarding the definition, diagnosis, background, description, aetiology, prevention, therapy, and prognosis. This provides an evidence map both for physicians and patients.

## Method

### Search strategy and selection criteria

Relevant articles were identified through a systematic literature search in the scientific databases Medline, Embase and PsycInfo up to the 26^th^ of May 2015 (Table 1). As Pubmed was used to search in Medline, only Pubmed is mentioned in this article. There were no restrictions on year of publication, language, and article or study type. Abstracts without full-text were excluded, as well as non-human studies. During the first selection step, potentially relevant references were selected based on screening of titles and or abstracts by two investigators independently (GM and SPK, both content area experts). Potentially relevant articles were included for full-text assessment. Articles on fatigue following acute Q-fever that could provide information on the following domains: diagnosis (i.e. definition and/or diagnosis), background/descriptive (i.e. incidence, prevalence, the course of fatigue and the role of co-morbidity, and other complaints besides fatigue), aetiology (i.e. pathophysiology, predictors), prevention/therapy, and prognosis, were selected.

**Table 1:** Search strategy used in Pubmed, Embase, and PsycInfo.

*Pubmed*	*Search terms*^†^	*Hits*
***6-5-2014***	("coxiella burnetii" OR “Q fever” OR “coxiella” OR “Q-fever” OR “rickettsia burnetii” OR “rickettsia burnetti” OR “rickettsiosis infection” OR “rickettsiosis rickettsia” OR “australian Q fever”)	
	**AND**	
	("fatigue" OR “syndrome” OR “Q fever Fatigue Syndrome” OR "Q-fever Fatigue Syndrome" OR QFFS OR QFS OR persisten* OR progress* OR “long term” OR “long-term” OR consequence* OR “chronic fatigue” OR tired*)	**494**
***26-5-2015***		**537**
***Embase***	***Search terms***^†^	***Hits***
***6-5-2014***	(exp Q fever/ OR Q fever.tw. OR exp Coxiella/ OR coxiella.tw. OR rickettsia burnetii.tw. OR rickettsiosis.tw.)	
	***AND***	
	(exp fatigue/ OR exp Fatigue Impact Scale/ OR exp chronic fatigue syndrome/ OR exp Fatigue Severity Scale/) OR fatigue.tw. OR QFFS.tw. OR QFS.tw. OR exp persistent infection/ OR (persistence or persistent).tw. OR (progression or progressive or consequence or consequential).tw. OR exp chronic fatigue syndrome/ OR (tired or tired' or tiredeness or tireding or tiredness or tiredness).tw.	**440**
***26-5-2015***		**489**
***PsycInfo***	***Search terms***	***Hits***
***6-5-2014***	(Q fever OR coxiella OR rickettsia burnetii OR rickettsia burnetti OR rickettsiosis OR rickettsiosis rickettsia)	**15**
***26-5-2015***		**18**

Literature search performed on 6th May 2014, updated on 26th May 2015, using the same search terms as in the first search.

^†^ Excluded from the search: Mesh term for rickettsiosis, as this labels for several typhus infections with a total hits of 15600 records; and the word ‘chronic’, to avoid inclusion of chronic Q-fever articles.

During the full-text assessment, articles without original or relevant data were excluded, upon an independent decision of each investigator, followed by consensus if needed. In case of any disagreement, the verdict of a third independent investigator was conclusive. If GM or SPK was a (co-)author of a potentially relevant article, a third independent investigator assessed and decided (both selection steps) on inclusion. GM and SPK translated non-English articles, if needed, native speakers where sought. If native speakers were unavailable, the corresponding author was contacted. If this yielded no response, the article was excluded.

Reference lists of included full-text articles were hand searched for additional relevant publications. If the title (or keyword in the title) suggested potential information on the topic, retrieval and full-text assessment followed. Finally, the World Health Organization, Centres for Disease Control and Prevention (CDC), Queensland Health, and gov.uk websites were searched for guidelines. Documents with relevant information that were identified during the search, but not classified as peer-reviewed articles, were included as grey literature.

### Quality assessment

The methodological quality of case-control and cohort studies was assessed with the Newcastle-Ottawa Scale (NOS) [22], that evaluates selection (maximum of 4 stars), comparability (maximum of 2 stars), and outcome (maximum of 3 stars). For economic evaluations, the ‘Evers checklist’ was used [23]. Case-series were assessed with a quality appraisal tool with 18 criteria. A score of ≥14 criteria (≥70%) was considered acceptable [24]. No specific instruments exist to assess the quality of case-reports, which in general is considered to have a low level of evidence. Therefore, the quality was assessed with a method based on the Coordination of Cancer Clinical Practice Guidelines in Europe (CoCanCPG), addressing eight criteria: an appropriate and clearly focused question, representative population, description of the survey method or data collection, outcome measures defined and described, response rate reported, and results valid and applicable to the targeted patient group. Articles could score: -/-, -, +/-, +, or ++ on these items. Although personal opinions were included to obtain a complete overview of all literature, these were not quality assessed as in general the quality is considered low.

### Data extraction and presentation

Study populations and definitions per included article were summarised in a separate table (S1 Table). Included articles were summarised in main domain tables: diagnosis, background/descriptive, aetiology, prevention/therapy, and prognosis (S2–S5 Tables). If articles contained additional information on other domains, this was noted in the main table. The following information was provided per article, if applicable: year of publication in chronological order starting with the oldest articles; first author; country; year of the study; study period and duration; study type; number of patients and controls; patient characteristics; co-morbidity; outcome measurement tools; intervention(s); outcome; conclusion(s)/recommendation(s); and the quality of the article. In case an article could not be assessed with any of the mentioned tools, this was stated in the table in column quality assessment (QA) as not applicable (NA). Grey literature was similarly ordered in a separate table (S6 Table).

## Results

### Inclusion of articles

**Fig 1:**
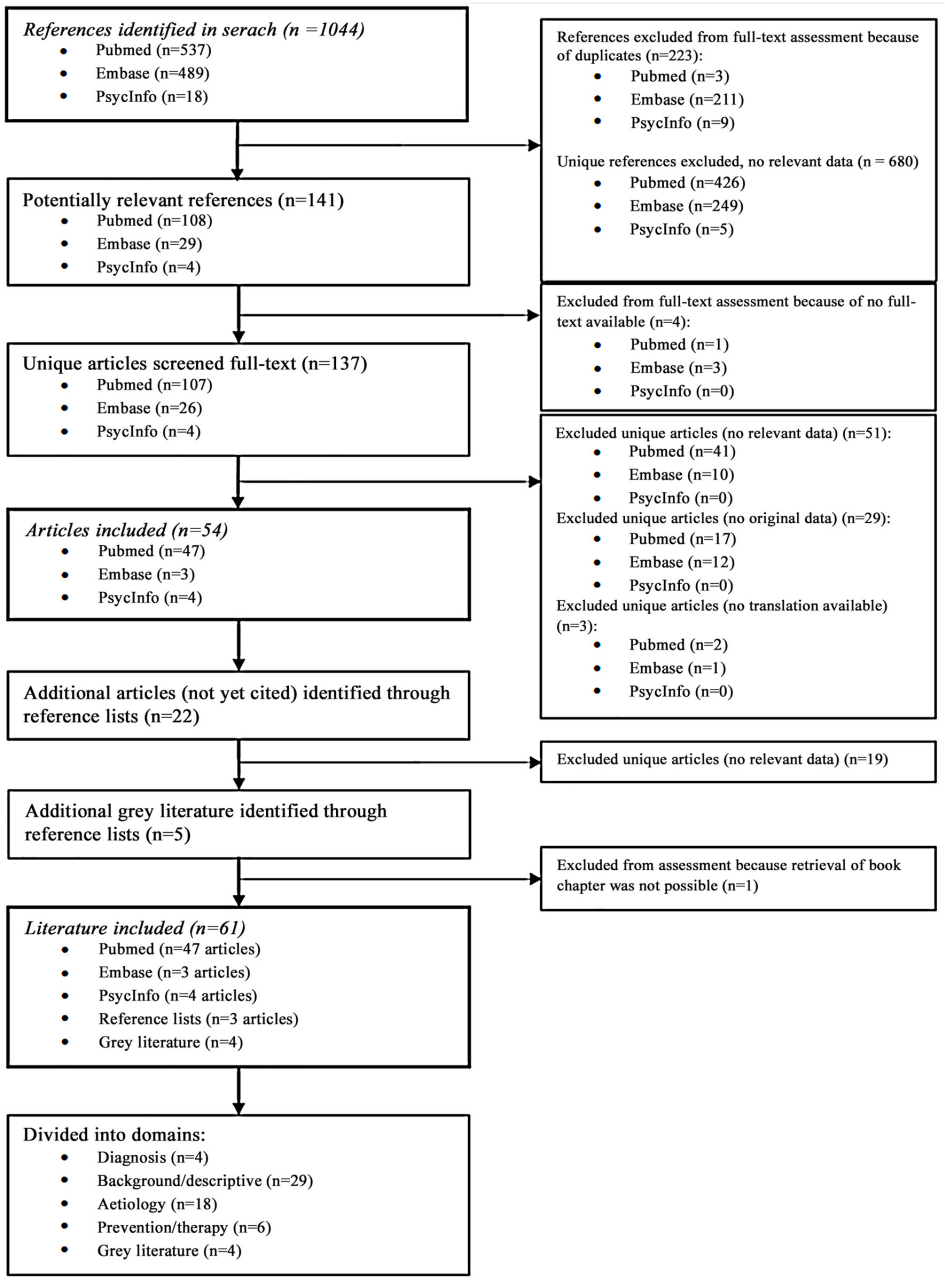
Flow diagram of identified literature.

The search yielded 1044 references (Fig 1); Pubmed n = 537, Embase n = 489, PsycInfo n = 18, of which 223 were duplicates. During the first selection phase, 680 references were excluded as not relevant, 141 identified as potentially relevant, and the full-text articles were searched. One full-text article (Spanish) could not be obtained from three different libraries and as the author could not be reached, the article was excluded. Three conference abstracts without full-text article were excluded. Of the remaining 137 full-text articles, 51 articles were deemed not relevant, 29 had no original data, and for three no translation was available (two Russian, one Japanese). The remaining 54 articles were included and hand searching their reference lists yielded 22 potentially relevant articles, of which three were included after full-text assessment. From the reference lists of included articles, we identified one guideline, one dissertation, two book chapters, and one economic report. After confirmation of relevance, these were included as grey literature except for one book chapter as retrieval was not possible. In total, we included 57 articles and four grey literature documents.

### Classification in domains

The 57 included articles were classified into one of the main domains: diagnosis (n = 4, S2 Table), background/descriptive (n = 29, S3 Table), aetiology (n = 18, S4 Table), and prevention/therapy (n = 6, S5 Table). As none of the included articles described the course of fatigue in QFS, no articles were classified into the domain prognosis. Grey literature (n = 4) is presented in S6 Table.

### Quality of included literature

From the four articles in the table diagnosis, one article was assessed with the NOS and scored 4/9 possible stars [25]. The remaining items (five stars) could not be assessed, as these items were not applicable for this study. The other three articles were personal opinions [10, 26, 27].

The quality of 21/29 articles in the domain background/descriptive was assessed with the NOS. Most articles had a moderate quality; however, none scored on all specific applicable criteria, mostly because of inadequate controls in the design or analysis. For four articles, not all items could be assessed, as these were not applicable for these studies. The quality of three case-reports (n = 1) was low [28–30]. The quality of one study regarding burden of disease was not assessed [31], as no standard quality assessment checklist was available for this study category. One economic evaluation scored well (16/19) [32]. Two articles were personal opinions [33, 34], and one was a personal observation [35].

The quality of 15/18 articles on aetiology was assessed with the NOS. Although none scored on all specific applicable criteria, the quality of the articles was considered moderate. Seven articles did not score on comparability although applicable, as they lacked a correction for other factors that might explain the outcome. For four articles, not all possible stars could be retrieved, as these items were not applicable for these studies. Two laboratory case studies were not quality assessed [36, 37], and one article was a personal opinion [38].

The quality of 2/6 prevention/therapy articles was assessed with the NOS. One study scored 4/9 stars, but none on comparability [39], while the other scored on 4/5 applicable items [6]. The quality of two case-reports (n = 1) [40, 41] was below average, as was that of the case-series (n = 3) [5], that scored on only 9/18 criteria. One article, a study protocol, was not quality assessed [42].

The Dutch QFS guideline was developed based on the AGREE criteria [43], and therefore considered to be of good quality [17]. The quality of the other grey literature was not assessed.

### Definition and diagnosis

Nineteen articles contained information on diagnosis of which four were classified in the main table diagnosis (S2 Table) [10, 25–27].

#### Terminology

The name QFS was introduced in 1992 [44]. Ever since, it has been debated whether fatigue following acute Q-fever is a separate entity compared to other forms of post-infective fatigue or CFS [27]. Some argue that chronic fatigue is a non-specific subjective state or symptom after Q-fever rather than a diagnosis [27]. Other consider QFS as a description of CFS implicating a specific micro-organism, and that this terminology might result in increased health-care costs [10]. Others stated that due to convincing evidence of a causal factor, QFS is a causally-defined subset of CFS, and that this factor should take precedence in the diagnostic statement [26]. Names used to indicate fatigue following acute Q-fever, include: residual asthenia following Q fever [38], postinfective fatigue or postinfective fatigue syndrome [10, 12, 18, 31, 45–47], postinfectious chronic fatigue [11], post-Q-fever debility syndrome [35], post-Q-fever chronic fatigue syndrome [35], qCFS [36], Q fever induced chronic fatigue syndrome [48], post-Q-fever fatigue or post-Q-fever fatigue syndrome [36, 49], post-(acute) Q-fever (fatigue) syndrome [5, 14, 26, 28, 33, 50], and most frequently Q-fever fatigue syndrome (QFS or QFFS) [6, 8, 10, 19–21, 26, 30, 33, 36, 39, 42, 50–52].

In conclusion, the term QFS has been used for years and seems generally accepted.

#### Definition of QFS

An overview of the study populations and definitions used is provided for articles (S1 Table) and grey literature (S6 Table). Seven articles lacked a definition of the study population or of QFS [10, 26, 27, 33–35, 38]. In 32 articles the study population was defined but QFS was not [3, 7, 9, 11, 12, 14, 18, 25, 31, 32, 36, 37, 45–47, 49, 52–67]. In five articles individual patients were considered to have QFS, without providing a definition [5, 28–30, 40]. Six articles provided a definition of QFS [6, 8, 19, 39, 42, 48], which has been used in articles in subsequent years [20, 21, 50, 51]. A detailed description of QFS is published in a thesis [44], but is based on a retrospective comparative-cohort study and is not available online. In the Dutch QFS guideline [17], QFS is defined as: a severe fatigue causing significant disabilities in daily life present for at least 6 months, with a temporal relationship with acute Q-fever, and not caused by co-morbidity. Fatigue should be absent before acute Q-fever or should have significantly increased since the infection.

In conclusion, there is no international uniform definition for QFS.

#### Diagnosis

No articles provided complete information on the diagnostic work-up. The Dutch guideline on QFS bases diagnosis on a combination of history, physical examination and laboratory examination excluding other causes of fatigue, and should at least include erythrocyte sedimentation rate, C-reactive protein (CRP), creatine kinase, thyroid stimulating hormone, leukocytes with differentiation, creatinine, alkaline phosphatase, alanin aminotransferase, calcium, glucose, ferritin, and a urinary sediment. Through the use of validated questionnaires fatigue severity should be objectified. Morbid obesity (BMI>40) and substance abuse should lead to refraining from diagnosing QFS. It is not possible to diagnose QFS in case of: depression (if this preceded current symptoms), schizophrenia, psychosis, dementia or eating disorders (unless already resolved for a minimum of 5 years) [17].

In conclusion, the Dutch guideline on QFS provides a clear diagnostic work-up.

### Background/descriptive

Of the 40 articles containing background/descriptive information, 29 were classified in the main table background/descriptive (S3 Table) [3, 7–9, 11, 12, 14, 28–35, 52, 53, 56–59, 61, 62, 64–69].

#### Incidence and prevalence of fatigue following *C*. *burnetii* infection

Fatigue following acute Q-fever was first described in 1960 [68]. Without indicating a time-relation with acute Q-fever, it was noted in 1990 that 4% of acute Q-fever cases had prolonged fatigue [53]. In 1992, it was stated that approximately 23% of study subjects developed QFS within 12 months following acute Q-fever [44]. Ever since, several studies on fatigue following acute Q-fever reported different prevalences. It was stated that 5–10% of patients experience residual asthenia six months after acute Q-fever and only few after one year [38]. In a reaction, it was underlined that a substantial proportion of acute Q-fever patients have symptoms similar to QFS for 6–9 months after the acute infection and then recover, but 8–10% of patients exhibit symptoms for at least a year [33]. This is similar to other reports, showing persistent symptoms for longer than two years [3], up to six years after the infection with 66% of patients reporting fatigue [14]. In Australia, QFS is the most common sequel of acute Q-fever reported to affect 10–15% of patients [70]. Higher percentages were described, with up to 28% of patients meeting the Centres for Disease Control and Prevention criteria for CFS 5 to 14 years after acute Q-fever, compared to none in the control group [8, 15]. The highest percentage of reported fatigue was 69% five years after acute Q-fever [9]. CFS criteria were met by 42% of *C*. *burnetii*-infected patients and 26% of controls [9, 15]. Ten years after acute Q-fever, 68% of patients reported fatigue of any duration [54], of whom 20% met the CFS criteria [15]. Excluding co-morbidity, 8% of patients met the CFS criteria compared to none of the controls [54]. *C*. *burnetii-*exposed compared to non-exposed subjects reported ten years later a fatigue prevalence of 65% vs. 35%, respectively, and 19% vs. 4% met the CFS criteria [7, 15]. In accordance, later results demonstrated fatigue to be more common after Q-fever compared to controls [58], up to two [61] and six years later [49, 69].

Post-infective fatigue following *Epstein-Barr virus*, *Ross River virus* or *C*. *burnetii* infection, was reported in 35% of cases after six weeks, 27% after three months, 12% after six months, and 9% after 12 months, regardless of the infective agent [12]. And, although not significantly different, 12 months after acute Q-fever, patients were more fatigued than after Legionnaires’ disease, while being younger and having less pre-existing health problems [11]. In patients with a lower respiratory tract infection who were *C*. *burnetii* seropositive 10–19 months after the acute illness, 40% reported clinically relevant fatigue, compared to 64% of seronegatives, concluding that patients have long-term health problems after a lower respiratory tract infection in general [64].

In conclusion, fatigue following acute Q-fever might not be specific but occurs frequently and may persist for years. A large variance in prevalence of fatigue after Q-fever is reported between countries, due to differences in definitions, study designs and populations, and measurement tools, which impairs direct comparisons.

#### Health status, burden of disease and economic impact

A sustained decrease in health status or health-related quality of life was reported [3, 58, 61]. Twelve months after acute Q-fever, 50% of patients had a reduced general quality of life [11]. Other studies show a significant linear improvement in health status after acute Q-fever, but it was still reduced after 24 months in more than one third of all patients [67]. Twenty-seven months after acute Q-fever, 52% of patients reported persistent symptoms and lower scores on 5/8 Short Form 36 (SF-36) scales [71] compared to uninfected controls [3]. Four years after acute Q-fever, patients also had a significantly reduced health status compared to healthy controls [65]. To obtain a detailed overview of the patients’ health, a combination of the complete Nijmegen Clinical Screening Instrument (NCSI) [72] with subdomains (Role Physical, Bodily Pain, Social Functioning, and Role Emotional) of the SF-36 was advised [25]. Two studies focus on the burden of disease of fatigue following acute Q-fever [31, 32], one also assessed the economic impact of the outbreak in the Netherlands [13]. In 1992, for Australian *C*. *burnetii*-infected abattoir workers the costs per year for medical care and loss of wages for endocarditis and for QFS were calculated [44]. QFS represented the largest burden of disease [32, 44]. Furthermore, others found that, although the number of disability adjusted life years was higher for influenza, on a per case basis, Q-fever was more severe, and overall the burden of disease was more than eight times higher than for influenza, due to long-term sequelae [31]. The estimated income loss was largest due to the accumulation over time as a consequence of the projected duration of sick leave, and QFS was estimated to be one of the major Q-fever-related economic cost during the Dutch outbreak [13].

In conclusion, there are clear indications that fatigue following acute Q-fever results in a high burden of disease, a major negative impact on the health status of patients, and has significant economic implications.

#### Work-related consequences

In 1960, it was noticed that the majority of acute Q-fever patients recovered within weeks and returned to work [68]. However, this convalescence period was prolonged in 25% of cases who were absent from work for more than 6 weeks, 20% longer than 8 weeks, up to 23 weeks [68]. The mean period of sick-leave increased with age [68]. Later studies revealed that following acute Q-fever, 40% of patients were absent from work for more than one month [62]. After 12–26 months 9% was unable to function at premorbid levels due to fatigue and diminished concentration while more than 30% had not fully resumed daily activities, in 81% due to fatigue [62]. Besides work-related consequences, patients were more likely to report functional impairment in performing daily activities than healthy controls [46]. Q-fever patients showed a reduced work participation, from 45% after three months to 19% after 12 months, versus 15% of patients with Legionnaires’ disease after 12 months [66]. Factors associated with reduced work participation were: having symptoms; a higher level of sorrow; being a former smoker (compared to never smoking); not consuming alcohol; and receiving treatment for health-related effects of Q-fever [66].

In conclusion, the majority of patients return to work within the first 12 months after acute Q-fever, although up to 20% reported reduced work participation.

#### Course of fatigue following acute Q-fever and the role of co-morbidity

Following acute Q-fever, 69% of patients self-reported fatigue, which dropped to 52% at six months to 26% at 12 months [57]. Studies using the NCSI found that severe fatigue following acute Q-fever improved from 73% at three months, to 60% at 12 months [11, 67]. Twelve to 26 months after acute Q-fever up to 59% of patients reported fatigue of which 44% had severe fatigue [59], whilst after 24 months 37% of patients compared to 3% of healthy controls, reported severe fatigue [67]. Higher rates of 51% were described four years after infection [65]. Most articles describe a continuous fatigue syndrome, up to 74 months after the initial infection [19], while relapsing or remittent fatigue patterns also seemed to occur [3], up to 57 months [19] after acute Q-fever. One article reported a fatigue free period of 2–4 months after acute Q-fever, eventually followed by QFS [5]. A disease period up to 20 years has also been reported [44]. Pre‐existing health problems were associated with a long-term reduced health status including fatigue [59, 62, 67].

In conclusion, the percentage of patients who experience severe fatigue following acute Q-fever slowly decreases over time, mainly in the first 6–12 months. Fatigue remains a persistent complaint in approximately 20% of patients, with varying percentages and variability in the reported course of fatigue following acute Q-fever, and may persist for up to 20 years.

#### Complaints besides fatigue

QFS is frequently compared to CFS, and patients who fulfil the international CFS criteria by definition have multiple symptoms [15, 16]. The mean number of symptoms was higher in Q-fever exposed subjects 10 years after exposure compared to controls [7]. Patients with post-infective fatigue, including Q-fever-related post-infective fatigue, reported more symptoms in general and fatigue-related symptoms in particular [46]. Twelve to 26 months after acute Q-fever 40% of patients reported additional complaints [62]. An overview of frequently reported complaints besides fatigue after acute Q-fever is given below.

Musculoskeletal complaints. Myalgia and arthralgia were frequent complaints of patients considered to have QFS [5, 6, 17, 28, 39, 40, 44, 70]. Musculoskeletal pain accompanied fatigue 12 months after several infections [12], and was associated with a higher age [18]. Myalgia was significantly more often present 5–14 years after acute Q-fever compared to controls [8]. Twelve to 26 months after acute Q-fever, 4% of patients reported myalgia [62]. Myalgia was a major complaint in 23% of working patients 12 months after acute Q-fever [66]. Arthralgia was reported by 69% of patients up to six years after acute Q-fever [14], and was more severe compared to controls [9]. Both myalgia and arthralgia were also described in up to 70% of patients after a laboratory documented *C*. *burnetii* infection [52]. Compared to controls, presumed QFS patients had a higher pain score [48].

Neurocognitive problems. Although some authors found no association between *C*. *burnetii* seropositivity and concentration difficulties [56], neurocognitive difficulties were described in patients with post-infective fatigue, including QFS patients, 12 months after primary infection [12]. In addition, older subjects reported more neurocognitive symptoms [18]. Twelve to 26 months after acute Q-fever, 4% of patients had difficulties concentrating [62]. Concentration and memory problems were also shown to be a major complaint in 24% of working Q-fever patients 12 months after the infection [66]. Although no difference was found in the frequency of memory problems between cases and controls, the severity was significantly higher after Q-fever [9]. A lack of concentration and short memory impairment within a year following acute Q-fever was also reported [17, 44], while another study found decreased concentration and mental acuity that could last up to 5–10 years [70].

Sleeping problems. Six years after acute Q-fever, 65% of patients reported a disturbed sleep pattern, which was significantly more frequent than in controls [14]. This was also reported by others [17, 29, 44, 70], including unrefreshing sleep [5].

Headache. Headache was frequently reported [5, 6, 17, 28, 30, 39, 52, 68, 70]. Twelve months after acute Q-fever, 24% of working patients reported frequent headaches [66]. Another study reported headache in 47% of patients six years after acute Q-fever [14]. Although the frequency of headache was similar to controls, the same authors found that the severity of headache was more profound in those after Q-fever [9].

Blurred vision. Blurred vision six years after acute Q-fever was similar to controls [14], but was more prevalent and more severe five years after acute Q-fever compared to controls in another study (34% vs. 18%) [9]. Blurred vision was also reported by others [17, 44]. Visual complaints were noted by 2% of patients 12 to 26 months after acute Q-fever [62].

Increased (night) sweating. Night sweats starting 6–12 months after acute Q-fever were described [70]. Twelve to 26 months after acute Q-fever, 3% of patients reported night sweats [62]. In comparison to controls, night sweats were more common after acute Q-fever [17, 44, 70]. Most QFS patients had this symptom for 5–10 years [70], up to 14 years [8, 28]. A combination of night sweating and increased sweating was also reported [30]. Increased sweating occurred with 53% more frequent after acute Q-fever compared to controls [14]. Others reported 53% of cases with increased sweating [5, 9]. Some authors considered abnormal sweating at least ten times a year as major QFS symptom [44].

Respiratory tract problems. Following acute Q-fever, 9% of patients complained of persistent chest symptoms [53]. Others reported that 47% of presumed QFS patients complained of cough and a sore throat with a mean symptom duration of four years [52]. Others reported these complaints also [17, 28–30, 39]. Five years after acute Q-fever, 51% of cases complained of breathlessness on exertion [9], compared to 32% of controls. Six years after acute Q-fever, 59% of patients complained of cough, 49% of breathlessness, and 51% of chest pain, all significantly more frequently than controls [14]. Furthermore, an association between QFS and bronchial asthma has been suggested [30].

Mood disorders. Patients with fatigue after acute Q-fever have been reported to experience increased irritability [14], mood disturbances [12, 17], and anger [70]. Mental problems, e.g. depression and unstable moods, can occur within a year following acute Q-fever [44], whereas, with regard to depression, most subjects were healthy before the infection [44]. Two years after acute Q-fever more psychosocial complaints were observed compared to controls [61]. Common symptoms of psychological distress were reported significantly more in patients with post-infective fatigue, including QFS patients, compared to healthy controls [46]. Others hypothesise that Q-fever-related fatigue might be explained by psychological distress, caused by uncertainty about their illness and repeated medical contacts that reinforce perceptions of ill health [7]. Some contradict this hypothesis [67]. Infection with *C*. *burnetii* was followed by depression in 10% of cases [53]. Three case-reports (all n = 1) [28–30] reported a *C*. *burnetii-*triggered depression, leading to thoughts of death [28], a near suicide attempt [30], and suicide [29]. The suggestion was that cytokine network abnormalities after a *C*. *burnetii* infection might underlie the onset of depression [28, 29, 73]. Although a possible relationship between high IgG phase II *C*. *burnetii-*antibodies and depression was suggested [69], others found no association between seropositivity, and depression, depressive ideas or overall psychiatric morbidity [56].

Other complaints. Other reported symptoms accompanying prolonged fatigue after Q-fever are severe malaise [40, 41], setback upon exertion and the need for prolonged rest after simple tasks [5, 8, 68], poor appetite [30, 68], gastrointestinal symptoms [6, 17, 29, 30, 44, 70], muscle fasciculation or spasms [8, 17, 41, 44, 70], dizziness [14, 17, 30], light intolerance [8, 19], tinnitus [28], taste disturbance [28, 29], loss of libido [17, 19], nasal and bronchial congestion [8, 17], and enlarged or painful lymph nodes [17, 70]. Bradycardia was postulated as a sign of QFS [35], and palpitations were described [30]. Even though reported in several studies [8, 17, 19, 44], alcohol intolerance was not statistically more frequent in the Q-fever group six years after acute Q-fever when compared to controls [14]. A slightly elevated body temperature (below 38 degrees Celsius) was described in QFS patients [5, 6, 28, 30, 39–41, 44, 70]. Up to 53% of assumed QFS patients felt feverish for four years [52].

In conclusion, besides fatigue as the main complaint, several nonspecific symptoms accompanying fatigue following *C*. *burnetii* infection were described. Commonly reported symptoms include musculoskeletal complaints, neurocognitive symptoms, sleeping problems, headaches, blurred vision, increased (night) sweating, respiratory complaints, and mood disorders.

### Aetiology

Of the 28 articles that contained information on aetiology, 18 were classified in the main table aetiology (S4 Table) [18–21, 36–38, 45–51, 54, 55, 60, 63].

#### Pathophysiology

Genetic variance and relationship with fatigue. No relation [3] or correlation [47] between genetic factors and QFS was found. A lack of a coherent set of gene expression correlating across cohorts argued against the genetic signature for post-infective fatigue or CFS [47]. In contrast, another study found similar gene expression patterns for QFS and CFS patients [48]. The frequency of human leukocyte antigen—group DR (HLA-DR)-11 was significantly increased in QFS patients compared to controls. Also, more polymorphic variants within the NRAMP1 gene differing from the wild type were found, as well as significant differences in allelic variant frequencies within interferon-y (IFNy) genes, but effects were thought to be multigenic and cumulative. It was hypothesised that QFS might result from individual variations in immune response to *C*. *burnetii* [50]. QFS patients differed in the frequency of HLA-DRB1*11 carriage and the 2/2 genotype of the IFNy intron 1 microsatellite compared to control groups [51]. Carriage was associated with reduced IFNy and interleukin(IL)-2 responses from stimulated peripheral blood mononuclear cells (PBMC) [51].

In conclusion, results regarding genetic variations in host immune responses in QFS were contradictory.

Immunological aspects. An immunological basis for QFS or other post-infective fatigue syndromes was debated in several articles. A reduction in reported fatigue correlated with improvement in the delayed-type hypersensitivity skin response and general health scores [45]. Resolving fatigue after acute infection seemed associated with improved cell-mediated immunity, supporting an immunological basis for post-infective fatigue [45]. Upregulation of 2’,5’-oligoadenylate synthetase (2-5AS) activity in PBMC of CFS patients was present, but a relation between *C*. *burnetii* antibody titres and 2-5AS activities lacked [55]. It was however suggested that *C*. *burnetii* infection is associated with 2-5AS activities in some CFS patients, as 2-5AS activities changed from positive to negative in one CFS patient when *C*. *burnetii* antibodies disappeared [55]. In acute Q-fever IL-6 and CRP seemed predictive of more severe disease, but no support was found that these were associated with prolonged fatigue [63]. Markers of inflammation and pro-inflammatory cytokine concentrations did not remain altered in patients with post-infective fatigue [12, 18].

In conclusion, no clear evidence exists with regard to an immunological basis involving 2-5AS, IL-6, and CRP for the development of QFS.

Immunomodulatory complex and cell-mediated immunity. Persistence of *C*. *burnetii* or its antigens resulting in chronic immune stimulation with subsequent fatigue [8, 19–21, 36, 37, 49], or causing dysregulation of the macrophage/T-lymphocyte axis with subsequently aberrant monokine and lymphokine production mediating symptoms [8], was hypothesised. Cytokine release patterns of PBMC of QFS patients were aberrant with an accentuated IL-6 release, a decreased number of IL-2 responders, and an increased number of IFNy responders [19]. *In vitro*, using human samples, an increased cellular immune response and cytokine dysregulation was found with increased levels of IL-6 and IL-10, and decreased level of IL-2 [70]. A significant correlation between IL-6 and scores for key and total symptoms was found [19]. The detection of low levels of *C*. *burnetii* DNA in bone marrow aspirates, thin needle liver biopsies, and blood mononuclear cells, supports cytokine dysregulation and immunomodulation caused by *C*. *burnetii* persistence [20]. Others showed a more complex interaction between host-regulated disease and persistent *C*. *burnetii* DNA carriage—either live, dormant, or dead but with undegraded DNA—in bone marrow, irrespective of clinical state [21]. An additional but variable factor of host regulation of cell-mediated immunity was postulated, determining the level of persistence and symptomatic outcomes. It was hypothesised that in Q-fever without sequelae, the process of multiplication of live *Coxiella* was largely confined to bone marrow, in contrast to QFS, in which a modulated immune response caused increased levels of *C*. *burnetii* genome in bone marrow with increased shedding into peripheral blood [21]. Subsequently, one of the core hypotheses postulated included the presence of an immunomodulatory complex, consisting of non-viable undegraded *C*. *burnetii* DNA or its antigens, causing an abnormal cell-mediated immune response via damaged macrophages [37]. This stops the patient from clearing the microbe completely, leading to ongoing production of pro-inflammatory cytokines and subsequently fatigue. In contrast to QFS patients, those who fully recovered from acute Q-fever had no immunomodulatory complex [37]. The bacteraemia is restricted by humoral and cell-mediated immunity, by clearing of *C*. *burnetii* DNA containing components with an immunomodulatory effect of cell-mediated immunity and dendritic cells causing dysregulation, cytokines and other immune mediators, giving rise to symptoms [70]. The complexes appeared more likely to be a residue of the original heavy seeding during the bacteraemia of the acute infection, rather than the product of an ongoing multiplication, destruction and renewal of infection [21]. QFS follows clinical overt infection, rarely subclinical infection, and the systemic symptoms of QFS may reflect a wide distribution of parasitized mononuclear phagocytes [36, 37]. In other patient cohorts, neither viable *C*. *burnetii* nor DNA in PBMC was detected [49].

In conclusion, several studies point towards cytokine dysregulation mediating symptoms in QFS. This may originate from an immunomodulatory complex consisting of non-viable undegraded *C*. *burnetii* DNA or its antigens. However, results regarding remnant *C*. *burnetii* DNA were contradictory.

Cardiac involvement in QFS. No ECG abnormalities excess in the *Coxiella*-exposed cohort with fatigue was found in comparison to controls [54]. Post-infective fatigue was associated with higher heartbeat discrimination accuracy, increased resting heart rate with decreased heart rate variability, and a lower pressure pain threshold [46]. The altered cardiac response was believed to be a stress response portraying an over-responsive system lacking dynamic flexibility [46]. Heightened interoceptive sensitivity with strong symptom correlation was also found. This suggests physiological hyper-vigilance and response inflexibility in post-infective fatigue [46].

In conclusion, there is no evidence for direct cardiac involvements in QFS, but there is some evidence for physiological hyper-vigilance and response inflexibility in patients with post-infective fatigue.

(Bio)psychological origin of QFS. It is unknown whether chronic fatigue following Q-fever is directly caused by the bacterium or if it is (bio)psychological in origin [38]. As subjective symptoms are difficult to quantify, it was stated that they might reflect an observational bias, *C*. *burnetii* strain or cultural differences, or genetic susceptibility [38]. In addition to the immune stimulation hypothesis, interpretations range from compensation-driven through psychogenic perpetuation of original symptoms or depression [8]. Q-fever patients with fatigue symptoms had higher somatisation scores, a higher tendency for hypochondriac worries and beliefs, a higher level of psychosocial complaints, and reduced quality of life [61]. The non-proven presumption was that Q-fever triggered fatigue development and that the risk of developing symptoms might be increased by hypochondriac features and a tendency to somatisation, supporting a biopsychological aetiology [61].

In conclusion, some studies supported the view of a biopsychological aetiology of QFS.

#### Predictors of post-infective fatigue syndrome, including QFS

Psychological factors and demographics. Post-infective fatigue appeared to be stereotyped across different infective triggers, and it was suggested that the host response rather than psychological or microbial factors determined ongoing symptoms [18]. No source of exposure was associated with developing persistent symptoms [3]. Premorbid and intercurrent psychiatric disorders were not predictive for post-infective fatigue [12]. In contrast to the biopsychological aetiology [61], it was recently suggested that psychological distress was not an important factor in explaining increased fatigue levels after acute Q-fever [67]. Although some found that gender was not a predictor [12], others found an overrepresentation of women in high severity groups for fatigue, mood disturbance and neurocognitive difficulties [60]. Being female or a young adult, and smoking were characteristics significantly associated with long‐term reduced health status including fatigue [62, 67]. In contrast, another study found no association between fatigue and age [59].

In conclusion, neither psychological nor microbial factors seem to predict post-infective fatigue, including QFS.

Severity of the acute illness.It was stated that one of the key risk factors for the development of post-infective fatigue, including QFS patients, is the severity of the acute illness [12]. Patients with post-infective fatigue had a longer mean duration of the acute illness, and more days in bed and days out of role during the acute phase compared to controls [18].The clinical expression of acute Q-fever seemed an essential factor in the subsequent sustained decrease in health status [58], which is supported by the finding that QFS usually follows acute Q-fever and rarely if ever asymptomatic infection [70]. Pre-existing health problems [62, 67], and hospitalisation, as an indicator of the severity of the initial infection, were also fatigue predictors [59, 62]. No symptoms during the acute Q-fever infection were predictors for persisting symptoms [3], nor did these determine the long-term health status [65]. Neither IL-6 and CRP levels nor antibiotic treatment during the acute infection were predictors for the development of prolonged fatigue [3, 63]. No relationship was found between fatigue and antibody titres six years after the Q-fever infection [49].

In conclusion, the severity of the acute Q-fever infection seems a key factor for worse long-term health status, including fatigue and QFS.

Genetic factors in predicting fatigue. A single nucleotide polymorphism (SNP) of the T allele IFNy+874T/A appeared to be the best predictor of increased fatigue after the acute phase of several infections, including *C*. *burnetii* [60]. While the C allele of IL-10-592C/A SNP exerted a protective effect on neurocognitive difficulties, the A allele IL-10-592 SNP and G allele IL-6-174G/C SNP were associated with increased mood disturbance [60].

In conclusion, as evidence is scarce, more research is needed regarding genetic factors predicting fatigue in QFS.

### Prevention/therapy

Eleven articles contained information on prevention/therapy of which six are classified in the main table prevention/therapy (S5 Table) [5, 6, 39–42].

#### Prevention

No articles on the prevention of QFS were found. The Dutch guideline on QFS proposes to advice patients within the first six months after acute Q-fever or after established QFS to: i) stay mentally and physically as active as possible, adjust pace if necessary; ii) alternate activities, also within activities; iii) keep fulfilling the role in daily life; iv) maintain a regular sleep-wake pattern; v) avoid focusing on fatigue; and vi) focus on feasible activities and appreciate accomplishments [17]. It is also proposed to explain that most patients recover within the first 6–12 months following acute Q-fever.

#### Antibiotic treatment

Four articles reported on the effect of long-term antibiotic treatment in assumed QFS patients [5, 6, 39, 40]. No randomised controlled trial (RCT) was found. Treatment with either 3 months of minocycline 200mg/day (n = 18), levofloxacine 200mg/day (n = 1), or erythromycin 400mg/day (n = 1), improved performance status and reduced fatigue [6], concluding that minocycline was useful in treating QFS [6]. In a pilot-study, treatment with three months of minocycline 100mg/day (n = 29), doxycycline 100mg/day (n = 26), or levofloxacin 200mg/day (n = 3), showed improvement in performance status, headache, and mean weekly temperature [39]. A case-series (n = 3) [5] and case-report (n = 1) [40] showed inconsistent results of treatment with long-term antibiotics. According to others, the positive effect of antibiotic treatment for QFS is not confirmed nor advised [17]. The efficacy of long-term antibiotic treatment is now tested in a RCT but results are not yet available [42].

In conclusion, available data on long-term antibiotic treatment for QFS are scarce and inconsistent.

#### Cognitive behavioural therapy (CBT) and graded exercise therapy (GET)

CBT proved effective in reducing symptoms and improving functioning in CFS patients [74, 75], and in chronic fatigue in chronic illnesses [76–78]. It was suggested as treatment option for QFS patients who experience psychological distress [61]. Based on CFS literature and similarities between CFS and QFS, CBT is advised in the Dutch QFS guideline, although suspected not to be beneficial for all patients [17]. The effectiveness of CBT treatment for QFS is currently under investigation [42]. Also GET is recommended for QFS patients, as proven effective in reducing fatigue in CFS [17].

In conclusion, although evidence is lacking, CBT and GET might be effective in reducing fatigue in QFS patients.

#### Treatment of QFS-related symptoms

Three articles (all n = 1) reported treatment of QFS-related symptoms [28–30]. The authors concluded that education and counselling about QFS and QFS-related symptoms should be provided to QFS patients [28]. Attention to the patient’s mental state is necessary in order to recognise accompanying symptoms, e.g. depressive thoughts, that should be treated [30], and involving a psychiatrist early ought to be considered [29]. This has been recognised before, where tricyclic antidepressants were beneficial treatment of mental problems after acute Q-fever [44].

In conclusion, education and counselling of patients about QFS and QFS-related symptoms seems important, as well as considering a patient’s mental state.

#### Alternative treatment

Alternative therapies for QFS patients were described (both n = 1), including Kampo formula Tsumura Hochu-ekki-To granules, which appeared not to be effective [40], and Kampo formula Shakuyaku-Kanzo-To granules, which resulted in alleviation of stiffness in hand and arm [41].

At present, evidence for the use of alternative treatment lacks.

## Discussion

This first systematic review on fatigue following acute Q-fever, includes 57 articles and four grey documents up to the 26^th^ of May 2015. The main limitation is the lack of a uniform definition of fatigue after Q-fever and the absence of a standardized diagnostic tool. In addition, the terminology both for fatigue and *C*. *burnetii*-related fatigue differed between publications and in time. Consequently, comparison of outcomes is difficult or impossible. Although not all articles could be quality assessed, these were nevertheless included as their information was considered valuable.

An international uniform definition of QFS, discriminating fatigue caused by *C*. *burnetii* from other post-infective fatigue syndromes and CFS is unavailable [19, 26, 36]. As the Dutch QFS guideline provides the most detailed description of QFS [17], we propose to use its definition and diagnostic work-up internationally. An international uniform definition provides the opportunity to achieve uniformity in diagnosis, treatment, and comparison of research results. It also provides recognition for physicians and acknowledgment for patients, reducing fear concerning uncertainty about their disease, providing an opportunity to continue their path to recovery [79, 80].

Whether fatigue following acute Q-fever is a separate entity compared to other forms of post-infective fatigue is debatable [10, 12, 18, 27, 44, 47, 81], but should not hamper the use of the term QFS.

Although differences in incidence and prevalence were reported, approximately 20% of patients remain chronically fatigued following an acute Q-fever infection. These differences can be explained by lack of recognition, uniform definition and diagnostic work-up, follow-up, and assessment tools. Using similar validated screening instruments is essential to compare studies [34]. Therefore, we advocate using validated screening instruments for measuring fatigue severity and disabilities, preferably with international available instruments [82], such as the Checklist Individual Strength or Chalder Fatigue Scale for fatigue [83, 84], and the NCSI, SF-36, or Sickness Impact Profile for disabilities [71, 72, 85]. This also helps to map the impact of QFS. The cut-off period of 6 months to diagnose QFS has been proposed as most patients recover spontaneously within this period, which corresponds with the internationally accepted definition for CFS [15, 16]. In QFS, fatigue frequently lasts beyond a year and mostly more than 5 to 10 years [8, 14]. Many nonspecific symptoms described accompanying fatigue in QFS were not systematically monitored as prospective data were unavailable. Most studies did not report the time-relation between these symptoms, fatigue, and the Q-fever infection, nor the frequency of occurrence. Therefore, it was not possible to list all symptoms possibly related to fatigue following *C*. *burnetii* infection nor provide a temporal or causal relationship. However, guidelines with regard to the examination of chronic fatigue should be followed to rule out other diseases which can cause chronic fatigue.

Several hypotheses regarding the underlying pathophysiological mechanism of QFS were proposed, but no conclusive answers have been identified yet. Research on the relationship between genetic factors and QFS is contradictory and scarce. Several studies point towards cytokine dysregulation mediating symptoms in QFS, including an immunomodulatory complex consisting of non-viable undegraded *C*. *burnetii* DNA and or its antigens. However, these results need further confirmation, as most studies regarding this topic have been done by the same study group and contradictory results exist with regard to the presence of *C*. *burnetii* DNA in QFS. Several queries exist regarding predictors of QFS. Neither psychological nor microbiological factors seemed to predict post-infective fatigue. Only the severity of the acute Q-fever infection appears a predictor of long-term reduced health status.

No uniformity exists regarding optimal treatment for QFS. Results from RCTs using long-term antibiotics are not available, and the available studies all suffer from several important limitations, such as the lack of a clear QFS description, the inclusion of patients with a symptom duration of 1–4 months, and the inclusion of patients with positive *C*. *burnetii* PCR at baseline, possibly indicating chronic Q-fever, and can therefore not be generalized. As the evidence of beneficial antibiotic treatment in QFS patients lacks, it should not be prescribed for QFS patients. The recommended treatment after diagnosis of QFS in the Dutch QFS guideline is based on CFS literature, and consists of CBT and, if available GET. The effectiveness of these treatments in QFS has not been proven yet. A randomised placebo-controlled trial in order to evaluate the efficacy of both long-term doxycycline and CBT in QFS patients is currently performed [42]. Treatment should at least focus on the provision of medical care, physical rehabilitation and additional psychological support [81]. Furthermore, physicians should be aware of accompanying complaints, especially depressive thoughts, which require treatment at an early stage [29]. Alternative treatments were only effective in one case-report and are therefore not recommended. Finally, the prognosis of QFS patients is unclear regardless if treated or not.

In conclusion, the occurrence and long-term persistence of fatigue following acute Q-fever, generally referred to as QFS, has major health-related consequences. Information on aetiology, prevention, treatment, and prognosis of QFS is underrepresented in the international literature. In order to facilitate comparison of findings, and as a platform for future preferably prospective studies, we propose a uniform definition of QFS and the use of uniform measurement tools. In addition, in order to facilitate comparison of long-term sequelae following several infectious agents, and as a platform for further preferably prospective studies, an international collaboration and a research agenda are desirable with regard to micro-organisms known for causing post-infective fatigue, in which *C*. *burnetii* should undoubtedly be included.

## Supporting Information

S1 File PRISMA 2009 checklist. (DOC)

S1 Table Overview of study populations and used definitions. (DOCX)

S2 Table Domain diagnosis. (DOCX)

S3 Table Domain background/descriptive. (DOCX)

S4 Table Domain aetiology. (DOCX)

S5 Table Domain prevention/therapy. (DOCX)

S6 Table Grey literature. (DOCX)
