# Supplementary material for: Fatigue following Acute Q-Fever: A Systematic Literature Review
Source: PLoS One. 2016 May 25;11(5):e0155884. doi: 10.1371/journal.pone.0155884 (PMC4880326; doi:10.1371/journal.pone.0155884)
Supplement: S1 Table — (DOCX) [file pone.0155884.s002.docx]

**S1 Table. Overview of study populations and used definitions**

| **Included articles** | **Study populations and used definitions** |
| --- | --- |
| 1960, O. Powell [1] | 1-2 yrs post AQF (AQF confirmed by demonstration CFT to *C.b.* with either a rise from zero to ≥1:32, or a titre in a single specimen of ≥1:256 in patients admitted to hospital or suspected of infection late in the illness) from Princess Alexandra Hospital, Brisbane. No definition for QFS or fatigue |
| 1990, S. Reilly [2] | All AQF cases diagnosed and monitored by the Public Health Laboratory in Plymouth between 1972 and 1988 out of FUO, respiratory infections, CNE, and hepatitis cases. Clinical and serological status assessed in 1989. AQF: ≥fourfold rise in phase II titre, or by a stable phase II titre ≥80 if there was strong clinical evidence of AQF. Past infection: evidence of past exposure to *C.b.* by single or sustained phase II titres ≥10 to ≤40, with QF not being considered to be causally related to the presenting complaint. No definition for fatigue |
| 1995, P. Harvey-Sutton [3] | No study population or QFS definition |
| 1996, B. Marmion [4] | 5-14 yrs post laboratory-proven AQF (AQF defined as a CFT titre of ≥1:256 or a 4-fold rise in phase II antibodies), QFS defined as: 1) incapacitating fatigue requiring prolonged rest after simple tasks; 2) nausea, persistent headache; 3) feeling feverish with profuse, odoriferous sweats at night, usually afebrile; 4) myalgia in any muscle group; 5) intermittent fasciculation of muscle fibres and muscle tenderness on palpation; 6) arthralgia without swelling, in any joint including costochondrals; 7) ethanol intolerance compared with capacity before AQF; and 8) interrupted sleep patterns, excessive and unreasonable irritability, unreliable short-term memory, and poor concentration. Less frequent complaints: bloating, irritable bowel syndrome, nasal and bronchial congestion, blurred vision, bright light intolerance, and enlargement and pain in lymph nodes. Definition CFS: according to the 1994 international CFS criteria [5] |
| 1996, J. Ayres [6] | 6 yrs post AQF [7, 8] (AQF defined as a CFT titre of ≥1:256 or a 4-fold rise in phase II antibodies), no QFS definition, but rather description of complaints being significantly more prevalent in past QF cases i.c.w. controls: joint pains, sleep disturbance, cough, sweating, irritability, chest pain, breathlessness, and dizziness |
| 1998, J. Ayres [9] | 5 yrs post AQF [7, 8] (AQF defined as a CFT titre of ≥1:256 or a 4-fold rise in phase II antibodies), no QFS definition, but rather description of complaints being significantly more prevalent in past QF cases i.c.w. controls: fatigue, sweating, breathlessness on exertion, blurred vision, with symptom severity in QF cases being higher for fatigue, blurred vision, sweating, memory deterioration, joint pains and headaches |
| 1998, B. Bennet [10] | PIFS patients from DIOS or from the University Health Service at the University of New South Wales whose symptoms have been present ≤4 wks |
| 1998, K. Kato [11] | Patients with chronic nonspecific symptoms, such as fatigue, joint aches, sleep disturbance, night sweats, myalgia affecting various muscle groups, nausea, persistent headache, and so on, without diagnosis or treatment history of QF and living in close contact with animals, presented between March 1996 and April 1997 to the Department of Internal Medicine and Psychosomatic Medicine, Nihon University Health Science Centre. Healthy controls: without/few complaints, who received annual examinations at the same hospital |
| 1998, I. Penttila [12] | Definition QFS patients: 1) severe incapacitating fatigue ≥6 mo post AQF, with symptom score >100; 2) presence of myalgia and arthralgia; and 3) abnormal sweats, particularly at night. In addition, most patients had other symptoms such as inappropriate exhaustion on minor exertion, muscle fasciculation, headaches, bright light intolerance, ethanol intolerance, interrupted and unrefreshing sleep patterns, irrational irritability, loss of libido, depression, impairment mental concentration and short-term memory. Resolving QFS: recruited in similar way after several yrs observation, but symptom score dropped from values >100 to ≤95. QF without QFS: 6 mo post AQF without complex of symptoms and low symptom score (1-35) |
| 1999, J. Scadding [13] | No study population or QFS definition |
| 2000, R. Harris [14] | Definition of QFS patients: conform [12]. Controls: conform [12] |
| 2002, J. Ayres [15] | 10 yrs post laboratory-proven AQF [7, 8] (AQF defined as a CFT titre of ≥1:256 or a 4-fold rise in phase II antibodies). Controls: no serological evidence of past exposure to *C.b*. Definition fatigue: according to the 1994 international CFS criteria [5, 16] |
| 2002, M. Wildman [17] | 10 yrs post laboratory-proven AQF [7, 8] (AQF defined as a CFT titre of ≥1:256 or a 4-fold rise in phase II antibodies). Definition fatigue: score ≥4 using the traditional scoring system for the fatigue questionnaire [18]. Definition ICF: fatigued and describing fatigue >50% of the time for 6 mo. Definition CFS: ICF and functional impairment and ≥4 additional symptoms according to the 1994 international diagnostic criteria [5]. Controls: no serological evidence of past exposure to *C.b.* |
| 2002, D. Raoult [19] | Definition QFS patient: residual asthenia following QF at 6 mo post AQF |
| 2002, B. Marmion [20] | No study population or QFS definition |
| 2002, M. Wildman [21] | No study population or QFS definition. Definition fatigue: according to the 1994 international CFS criteria [5, 16] |
| 2003, T. Hatchette [22] | 3 and 27 mo post AQF [23], no QFS definition. Controls: without AQF during same outbreak cohort |
| 2003, K. Helbig [24] | Definition QFS patients: conform [12]. Recovered QFS: conform [12] |
| 2003 K. Ikuta [25] | CFS based on the 1988 CDC working case definition [26] and the Ministry of Health and Welfare of Japan, from Tottori University Hospital, Yonago, and from Osaka University Hospital, Osaka, Japan. Healthy controls: from Tottori University Hospital Yonago |
| 2004, Y. Arashima [27] | Definition QFS patients: prolonged nonspecific complaints, with general fatigue of unknown origin, or headache, slightly elevated body temperature (37-37,5˚C), arthralgia, or myalgia, with *C.b.* seropositive defined by IgMII ≥1:32 or IgGII ≥1:128 (or ≥1:64 if antibody for *B. henselae* was negative) and/or detectable *C.b.* DNA, for 3 mo till 4 yrs, between July and November 2001 from the Department of Internal Medicine of the Nihon University School of Medicine, Tokyo |
| 2004, H. Thomas [28] | 8 yrs post recruitment in 1991 from a random sample of farmers drawn from the Ministry of Agriculture, Fisheries and Food June Agricultural Census lists of agricultural holdings, with *C.b.* seropositivity defined by IgGII ≥1:32. No QFS definition |
| 2005, B. Marmion [29] | Definition UK cases: 12 yrs post laboratory-proven AQF [7, 8] (AQF defined as a CFT titre of ≥1:256 or a 4-fold rise in phase II antibodies). Definition fatigue: conform [17]. Definition Australian QFS cases: conform [12, 14], 9 mo-5 yrs post AQF. Definition fatigue: according to the 1994 international CFS criteria [5] |
| 2005, K. Helbig [30] | Definition QFS patients: as in [12, 29]. Definition AQF with asymptomatic recovery: 12 yrs post laboratory-proven AQF [7, 8] (AQF defined as a CFT titre of ≥1:256 or a 4-fold rise in phase II antibodies), with complete recovery without QFS or other chronic sequel. Definition QIE: clinical evidence of endocarditis by observation of vegetations on ultrascan or on histopathological examination of the diseased valve, and a compatible serological profile defined by IgGI and II >320, low or no IgM and IgAI ≥160, and PCR positive examination of valve vegetation specimens and in some instances by isolation of *C.b.* in cell culture or laboratory animals, Caucasians mainly from New South Wales and Queensland |
| 2005, E. Iwakami [31] | Definition CFS patients: according to the 1994 international CFS criteria [5, 32], in combination with proven *C.b.* infection defined by IgG ≥1:128 (or ≥1:64 if *B. henselae* was negative), or IgM ≥1:32, and/or detectable *C.b.* DNA, for 8 mo till 11 yrs. Definition QFS patients: nonspecific complaints such as CF, slightly elevated body temperature, headache, arthralgia and myalgia of unknown origin for several mo or longer, but not meeting the 1994 international CFS criteria, in combination with a confirmed *C.b.* infection defined by IgG ≥1:128 (or ≥1:64 if *B. henselae* was negative), or IgM ≥1:32, and/or detectable *C.b.* DNA by n-PCR, regardless of the presence or absence of pre-existing infection, for 1 mo till 10 yrs |
| 2006, I. Hickie [33] | Patients from DIOS with symptoms ≤6 weeks assessed at 3 and 6 wks, and 3 and 12 mo post AI, without pre-existing medical disorders or drug use likely to be associated with prolonged fatigue. Provisional PIFS: if SOMA scores at all time points up to and including 3 mo exceeded the established threshold score [34]. Confirmed PIFS: CFS at 6 mo post AI according to the 1994 international CFS criteria [5]. Controls: recovered promptly from the same infection |
| 2007, D. Ledina [35] | Definition QFS patients: between January 2000 and December 2004 at Split University Hospital, Croatia. 1) 12 mo post AQF complaints of morning fatigue, disrupted sleep, headache, prolonged fatigue >24 hours post exertion, muscle pain, persistent slightly elevated body temperature, without CQF, meeting the 1994 international CFS criteria [5]. 2) 2 mo post AQF no symptoms, than start neck pain with 6 mo post AQF start of fatigue, insomnia, headache, sweating, unrefreshing sleep, for 12 mo, meeting the 1994 international CFS criteria [5] with positive ELISA IgG 1.6 and IgA 1.4. 3) 4 mo post AQF start symptoms of fatigue, disrupted sleep, headaches, muscle and joint pain, for 7 mo, meeting the 1994 international CFS criteria [5], with positive ELISA IgG 2.4 and IgA 1.5 |
| 2007, U. Vollmer-Conna [36] | PIFS patients from DIOS assessed at 1, 2, 3, 6, and 12 mo post AI, with confirmed PIFS if symptoms persisted beyond 6 mo with a score of ≥3 at all time points on the empirically derived subscale SOMA, without alternative explanations for ongoing illness and meeting the 1994 international CFS criteria [5] |
| 2009, B. Marmion [37] | Samples from 11 patients ≥12 yrs post laboratory-proven AQF [7, 8], of whom 1 patients had slightly elevated body temperature, late-stage QIE |
| 2009, L. Zhang [38] | Definition CFS/ME: idiopathic CFS/ME according to the 1994 international CFS criteria [5], from Bristol, London, and New York, and CFS/ME from [39, 40]. Definition Q-CFS/ME: CFS/ME according to the 1994 international CFS criteria [5] triggered by laboratory documented QF, from Birmingham. Definition endogenous depression: fulfilled DSM-IV criteria, from Bristol and surrounding area. Definition healthy blood donors: from Dorset National Blood Service [41]. Excluded were psychiatric diseases, smoking previous yr, alcohol or drugs abuse, current use or ≤3 mo of antibiotics, steroids, cytotoxic drugs or antidepressant |
| 2010, Y. Kadota [42] | PIFS patients from DIOS or from a tertiary referral assessment clinic at a public teaching hospital in Sydney, and patients’ current symptom profiles had to fulfill the 1994 international CFS criteria [5] |
| 2010, O. Sukocheva [43] | Samples from patients 12 yrs post laboratory-proven AQF [7, 8], classification of patients into clinical groupings according to asymptomatic recovery or presence of QFS with or without other co-morbidity [17, 44], with a chosen subset from 1) recGr3, AQF with asymptomatic recovery; 2) QFSGr5, AQF followed by QFS without co-morbidity; 3) QFSGr6, AQF followed by QFS with fatigue-associated co-morbidity |
| 2010, G. Limonard [45] | 12 mo post laboratory-proven AQF (AQF defined as any inhabitant of the outbreak cluster area who presented with compatible clinical symptoms and a positive IFA serology, with an IgMII and IgGII ≥1:64 or seroconversion with 4-fold rise in antibody titre during FU). Controls: from neighbourhood of QF patient without QF history, with negative QF serology |
| 2010, G. Limonard [46] | Post laboratory-proven AQF (AQF defined as any inhabitant of the outbreak cluster area who presented with ≥1 compatible clinical symptoms (fever, fatigue, chills, headache, myalgia, sweats, cough) and the demonstration of *C.b.* infection, as evidence by: 1) seroconversion or 4-fold rise in antibody titre using CFT in samples taken ≥14 days apart; 2) presence of IFA IgMII and IgGII ≥1:64; or 3) a positive serum PCR) assessed at baseline, 3, 6, 12 mo. Definition CQF: any inhabitant of outbreak cluster area with clinical entity compatible with endocarditis, vascular infection, osteoarticular infection, chronic hepatitis, or pregnancy, with an IgGI ≥800, for ≥6 mo post AQF |
| 2011, G. Morroy [47] | 12-26 mo post AQF (AQF according to the Dutch notification criteria [48] defined as a laboratory confirmation of QF with a seroconversion or a 4-fold rise in antibody titre between 2 subsequent tests with 2-4 wks time interval using CFT or IFA, and clinical presentation of fever, pneumonia or hepatitis, ≥18 yrs, notified in 2007/2008. Excluded: unknown onset of QF infection, incomplete questionnaires and questionnaires completed by another person |
| 2011, H. van Woerden [49] | 6 yrs post AQF (AQF defined as those who had clinical symptoms and serological evidence of AQF as demonstrated by an IgMII ≥80, or a fourfold rise on sequential CFT in 2002). Definition controls: who worked in the same factory but had no symptoms of AQF and no serological evidence of infection with no IgM, no CFT and no IgGI or IgGII at the time of the outbreak |
| 2011, S. Galbraith [50] | Caucasian PIFS patients from DIOS with unexplained illness persisting ≥6 mo with a score of ≥3 at all time points on the empirically derived subscale SOMA, without alternative explanations for ongoing illness and meeting the 1994 international CFS criteria [5]. Controls: recovered promptly from the same infection |
| 2012, B. Piraino [51] | Caucasian adult PIFS patients from DIOS [33] assessed at baseline, 2-3 wks, 4-6 wks, followed by 3-mo interval until 12 mo post AI |
| 2012, B. Strauss [52] | 2 yrs post laboratory-proven AQF [53]. Controls: without registered indicator for QF infection, from same general practitioners as study patients |
| 2012, G. Morroy [54] | 12-26 mo post AQF (AQF according to the Dutch notification criteria [48] defined as a laboratory confirmation of QF and clinical presentation with fever, pneumonia or hepatitis, notified in 2007/2008) |
| 2012, Y. Arashima [55] | Definition QFS patient: 3 mo post AI with general fatigue, slightly elevated body temperature (37˚C or higher), cough, night sweats, arthralgia, noise in his ears, taste disturbance, and headache, without abnormalities in physical examination, laboratory examination including cultures and additional tests (X-rays, abdominal ultrasound, echocardiography, treadmill exercise test), but with positive n-PCR for *C.b.,* IgGII 1:64 |
| 2012, D. Raoult [56] | No study population or QFS definition |
| 2012, H. Hussain-Yusuf [57] | Patients 6 yrs post serological evidence of AQF in 2002 [58]. Controls: worked in the same factory but were serologically negative for QF at the time of the outbreak |
| 2012, J. Oosterheert [59] | No study population or QFS definition |
| 2012, S. Yakubo [60] | Definition QFS patients: general fatigue, nausea, stomach pain, abnormal sensation in the mouth, sore throat, and trouble sleeping, with IgGI 1:256 |
| 2013, S. Keijmel [61] | Definition QFS patients: according to the Dutch guideline on QFS [62], referred to Radboud university medical center, Nijmegen, the Netherlands; adults (non-pregnant, non-lactating), ≥18 yrs, with laboratory-proven AQF since 2007 and/or positive serology fitting a past infection with *C.b.*, and being severely fatigued (CIS fatigue ≥35) for ≥6 mo, and being disabled because of fatigue (SIP total score ≥450), with a reference to AQF and absence of fatigue before the episode of AQF or a significant increase ever since. Excluded: CQF [63], AQF in the presence of risk factors for developing CQF necessitating prophylactic use of doxycycline, pregnancy or unwillingness to use effective contraceptives during the study, imminent death, inability to give informed consent, allergy or intolerance to doxycycline, somatic or psychiatric illness explaining chronic fatigue, current enrolment in other investigational drug trials or receiving investigational agents, receiving or having received AB >4 wks potentially active against *C.b.*, use of barbiturates, phenytoin, or carbamazepine, moderate or severe liver disease, current engagement in legal procedure for financial benefits |
| 2013, S. Yakubo [64] | Definition QFS patient: 6 yrs post AI with general malaise, spasm left hand, slightly elevated body temperature, without abnormalities in physical examination, laboratory examination including pharyngeal culture and additional tests (chest X-ray, X-ray of larynx/pharynx/ears and paranasal sinuses, ECG, abdominal ultrasound, brain CT, EEG), with negative n-PCR for *C.b.*, IgMI and IgMII <1:16, IgGI <1:16, IgGII 1:32. Six mo after presentation IgGI 1:128 |
| 2013, M. van Asseldonk [65] | All notified, hospitalised, deceased and non-reported cases of QF, determined from [66] and [67] |
| 2013, J. van Loenhout [68] | 12 mo post AQF, patients ≥18 yrs diagnosed with QF in 2010 and 2011, who fulfilled the Dutch notification criteria for QF [69] were eligible for participation |
| 2013, S. Yakubo [70] | Definition QFS patient: 2 mo post AI with severe fatigue, general malaise, arthralgia, myalgia, persistent slightly elevated body temperature (around 37˚C), whole-body lassitude, without abnormalities in physical examination, laboratory examination including a pharyngeal culture and additional tests (chest X-ray, ECG), but with positive n-PCR for *C.b.*, without positive antibodies |
| 2013, R. Brooke [71] | QF notified patients with onset symptoms between 1 January 2009 and 31 December 2013. A(H1N1)pdm09 notified patients, reflected by influenza-like-illness registration from the Dutch Sentinel General Practice Network for influenza-like-illness from NIVEL Netherlands Institute for Health Services Research between 27 April 2009 and 26 April 2010 |
| 2013, Y. Arashima [72] | Definition QFS patients: 18 mo post AI with general fatigue, cough, dyspnoea, sputum, breathing difficulty, slightly elevated body temperature, headache, poor appetite, copious sweating, night sweating, nausea, vomiting, palpitations, and dizziness, without abnormalities on physical examination, laboratory examination (besides slight liver dysfunction), but with positive n-PCR for *C.b.*, IgMII 1:16, IgGII 1:128 |
| 2014, M. Kremers [73] | Post laboratory-proven AQF (AQF according to the Dutch notification criteria [48] defined as symptomatic patients with positive PCR for *C.b.* DNA in serum samples before the development of an IgMII antibody response measured by IFA or ELISA), between April 2009 and August 2009, and assessment 4 yrs post AQF, all who were still alive, ≥18 yrs and of whom a 12 mo FU sample was present |
| 2014, J. van Loenhout [74] | Definition QF study population [75]: notified patients 1 yr post AQF in 2010 and 2011 (AQF according to the Dutch notification criteria defined as a laboratory confirmation of QF with a seroconversion or a 4-fold rise in IgG antibody titre in a paired serum sample with ≥2 wks time interval using CFT or IFA, presence of IgMII antibodies, positive PCR or culture in blood or respiratory material, presence of phase I antibodies, combined with a clinical presentation with fever, pneumonia or hepatitis, an onset of illness within previous 90 days [69], and ≥18 yrs. Definition Legionnaires disease study population: notified patients, 1 yr post Legionnaires’ disease in 2010 (Legionnaires’ disease according to the Dutch notification criteria defined as matching clinical symptoms, usually pneumonia, confirmed by at least 1 but preferably 2 of the laboratory diagnostic test: isolation of Legionella-species from respiratory secretions or blood; Legionella pneumophila-antigen in urine by radio-immuno-assay, ELISA, or immuno-chromatographic assay; Legionella-species by PCR in clinical material; significant titre of IgM by ELISA; significant titre elevation of antibodies. Healthy controls: via advertisements in local newspapers in the city of Nijmegen area. Excluded controls: underlying respiratory illness |
| 2014, A. van Dam [76] | 10-19 mo post LRTI as diagnosed by general practitioner between 1 May 2009 and 30 September 2009 in provinces of Northern Brabant and Gelderland, categorized into following ICPC groups: acute bronchitis, influenza, pneumonia, and other LRTI who were initially tested for QF, ≥18 yrs and ≤75 yrs. Definition QF positive: LRTI patients with positive diagnostic tests by either PCR, IFA, or CFT |
| 2015, J. van Loenhout [77] | Over a period of 24 mo (assessed at 3, 6, 9, 12, 18 and 24 mo) post laboratory-proven AQF in 2010 and 2011 (AQF according to the Dutch notification criteria [69]), ≥18 yrs |
| 2015, J. van Loenhout [78] | Definition notified QF patients: 4 yrs post laboratory-proven AQF in 2007 and 2008 (AQF according to the EU case definition [79] with laboratory criteria (isolation of *C.b.* from clinical specimen; detection of *C.b*. nucleid acid in clinical specimen; *C.b*. specific antibody response (IgGII or IgMII)), epidemiological criteria (exposure to common source; animal to human transmission), and clinical criteria (fever, pneumonia and/or hepatitis), onset of disease <90 days, ≥18 yrs. Definition non-notified QF patients: 4 yrs post laboratory-proven QF in 2008 and 2009 (according to the EU case definition, but only fulfilling the laboratory criteria and not the clinical criteria of fever, pneumonia or hepatitis), onset of disease <90 days, ≥18 yrs |
| 2015, J. van Loenhout [80] | Definition QF study population [75]: notified patients assessed 3, 6, 9 and 12 mo post laboratory-proven AQF in 2010 and 2011 (AQF according to the Dutch notification criteria), ≥18 yrs. Definition Legionnaires disease study population [75]: notified patients 12 mo post Legionnaires’ disease in 2010 (Legionnaires’ disease according to the Dutch notification criteria) |

***Abbreviations:*** AI= Acute infection, AQF= Acute Q-fever, *B. henselae= Bartonella henselae*, *C.b.=* *Coxiella burnetii,* CDC= Centres for Disease Control and Prevention, CF= Chronic fatigue, CFS(/ME)= Chronic fatigue syndrome (/myeloencephalitis), CFT= Complement fixation test, CIS= Checklist Individual Strength, CNE= Culture negative endocarditis, CQF= Chronic Q-fever, DIOS= Dubbo Infection Outcomes Study**,** cohort study of subjects ≥16 yrs followed from the onset of a confirmed and documented AI due to EBV; *C.b.*; or RRV ≤6 wks post AI until complete recovery, DSM-IV= Diagnostic Statistical Manual of Mental Disorders, EBV= *Epstein-Barr virus*, ECG= Electrocardiography, ELISA= enzyme-linked immunfluorsorbent assay, EU= European Union, FU= Follow-up, FUO= Fever of unknown origin, I.c.w.= In comparison with, ICF= Idiopathic chronic fatigue, ICPC= International classification of primary care, IFA= Immunofluorescence assay, IgA= Anti-phase IgA, IgG= Anti-phase IgG, IgGI= Anti-phase IgG I titre, IgGII= Anti-phase IgG II titre, IgM= Anti-phase IgM, IgMI= Anti-phase IgM I titre, IgMII= Anti-phase IgM II titre, LRTI= Lower respiratory tract infection, Mo= Month(s), (n-)PCR= (nested-) Polymerase chain reaction, PIF(S)= Post-infective fatigue (syndrome), Q-CFS(/ME)= Q-fever induced chronic fatigue syndrome (/myeloencephalitis), QF= Q-fever, QF(F)S= Q-fever fatigue syndrome, or Post-Q-fever chronic fatigue syndrome, or Post-Q-fever debility syndrome, or PQFS= Post-(acute)Q-fever (fatigue) syndrome, (Q)IE= (Q-fever induced) Infective endocarditis, Ref= Reference, RRV= *Ross River virus*, SIP= Sickness Impact Profile, SOMA= Empirically derived subscale of the SPHERE, used to record PIFS or illness duration. This reliably predicts disability and reflects patients’ and doctors’ reports of reasons for presentation to primary care. Scores ≥3 represents a clinically-significant fatigue state, UK= United Kingdom, Wks= Weeks, Yr(s)= Year(s)

**References**

1. Powell O. "Q" fever: clinical features in 72 cases. Aust Ann Med. 1960;9:214-23. PubMed PMID: 13737616.

2. Reilly S, Northwood JL, Caul EO. Q fever in Plymouth, 1972-88. A review with particular reference to neurological manifestations. Epidemiol Infect. 1990;105(2):391-408. Epub 1990/10/01. PubMed PMID: 2209742; PubMed Central PMCID: PMCPmc2271878.

3. Harvey-Sutton PL. Post-Q fever syndrome. Med J Aust. 1995;162(3):168. Epub 1995/02/06. PubMed PMID: 7854246.

4. Marmion BP, Shannon M, Maddocks I, Storm P, Penttila I. Protracted debility and fatigue after acute Q fever. Lancet. 1996;347(9006):977-8. Epub 1996/04/06. PubMed PMID: 8598796.

5. Fukuda K, Straus SE, Hickie I, Sharpe MC, Dobbins JG, Komaroff A. The Chronic Fatigue Syndrome: A Comprehensive Approach to Its Definition and Study. Ann Intern Med. 1994;121(12):953-9. doi: 10.7326/0003-4819-121-12-199412150-00009.

6. Ayres JG, Smith EG, Flint N. Protracted fatigue and debility after acute Q fever. Lancet. 1996;347(9006):978-9. Epub 1996/04/06. PubMed PMID: 8598797.

7. Smith G. Q fever outbreak in Birmingham, UK. Lancet. 1989;2(8662):557. PubMed PMID: 2570248.

8. Smith DL, Ayres JG, Blair I, Burge PS, Carpenter MJ, Caul EO, et al. A large Q fever outbreak in the West Midlands: clinical aspects. Respiratory Medicine. 1993;87(7):509-16. PubMed PMID: 8265838.

9. Ayres JG, Flint N, Smith EG, Tunnicliffe WS, Fletcher TJ, Hammond K, et al. Post-infection fatigue syndrome following Q fever. QJM. 1998;91(2):105-23. Epub 1998/05/14. PubMed PMID: 9578893.

10. Bennett BK, Hickie IB, Vollmer-Conna US, Quigley B, Brennan CM, Wakefield D, et al. The relationship between fatigue, psychological and immunological variables in acute infectious illness. Aust N Z J Psychiatry. 1998;32(2):180-6. Epub 1998/05/20. PubMed PMID: 9588296.

11. Kato K, Arashima Y, Asai S, Furuya Y, Yoshida Y, Murakami M, et al. Detection of Coxiella burnetii specific DNA in blood samples from Japanese patients with chronic nonspecific symptoms by nested polymerase chain reaction. FEMS Immunol Med Microbiol. 1998;21(2):139-44. Epub 1998/07/31. PubMed PMID: 9685003.

12. Penttila IA, Harris RJ, Storm P, Haynes D, Worswick DA, Marmion BP. Cytokine dysregulation in the post-Q-fever fatigue syndrome. QJM. 1998;91(8):549-60. Epub 1999/01/20. PubMed PMID: 9893758.

13. Scadding JG. Fatigue syndromes. QJM. 1999;92(5):293-4. Epub 2000/01/01. PubMed PMID: 10615486.

14. Harris RJ, Storm PA, Lloyd A, Arens M, Marmion BP. Long-term persistence of Coxiella burnetii in the host after primary Q fever. Epidemiol Infect. 2000;124(3):543-9. Epub 2000/09/12. PubMed PMID: 10982079; PubMed Central PMCID: PMCPmc2810941.

15. Ayres JG, Wildman M, Groves J, Ment J, Smith EG, Beattie JM. Long-term follow-up of patients from the 1989 Q fever outbreak: no evidence of excess cardiac disease in those with fatigue. QJM. 2002;95(8):539-46. Epub 2002/07/30. PubMed PMID: 12145393.

16. Wessely S, Chalder T, Hirsch S, Wallace P, Wright D. The prevalence and morbidity of chronic fatigue and chronic fatigue syndrome: a prospective primary care study. Am J Public Health. 1997;87(9):1449-55. PubMed PMID: 9314795; PubMed Central PMCID: PMC1380968.

17. Wildman MJ, Smith EG, Groves J, Beattie JM, Caul EO, Ayres JG. Chronic fatigue following infection by Coxiella burnetii (Q fever): ten-year follow-up of the 1989 UK outbreak cohort. QJM. 2002;95(8):527-38. Epub 2002/07/30. PubMed PMID: 12145392.

18. Chalder T, Berelowitz G, Pawlikowska T, Watts L, Wessely S, Wright D, et al. Development of a fatigue scale. J Psychosom Res. 1993;37(2):147-53. PubMed PMID: 8463991.

19. Raoult D. Q fever: still a mysterious disease. QJM. 2002;95(8):491-2. Epub 2002/07/30. PubMed PMID: 12145387.

20. Marmion BP, Harris RJ, Storm PA, Semendric L. Q fever: still a mysterious disease. QJM. 2002;95(12):832-3. Epub 2002/11/28. PubMed PMID: 12454328.

21. Wildman MJ, Ayres JG. Q fever: still a mysterious disease. QJM. 2002;95(12):833-4. Epub 2003/01/15. PubMed PMID: 12524714.

22. Hatchette TF, Hayes M, Merry H, Schlech WF, Marrie TJ. The effect of C. burnetii infection on the quality of life of patients following an outbreak of Q fever. Epidemiol Infect. 2003;130(3):491-5. Epub 2003/06/27. PubMed PMID: 12825734; PubMed Central PMCID: PMCPmc2869986.

23. Hatchette TF, Hudson RC, Schlech WF, Campbell NA, Hatchette JE, Ratnam S, et al. Goat-associated Q fever: a new disease in Newfoundland. Emerg Infect Dis. 2001;7(3):413-9. doi: 10.3201/eid0703.010308. PubMed PMID: 11384518; PubMed Central PMCID: PMC2631794.

24. Helbig KJ, Heatley SL, Harris RJ, Mullighan CG, Bardy PG, Marmion BP. Variation in immune response genes and chronic Q fever. Concepts: preliminary test with post-Q fever fatigue syndrome. Genes Immun. 2003;4(1):82-5. Epub 2003/02/22. doi: 10.1038/sj.gene.6363912. PubMed PMID: 12595908.

25. Ikuta K, Yamada T, Shimomura T, Kuratsune H, Kawahara R, Ikawa S, et al. Diagnostic evaluation of 2', 5'-oligoadenylate synthetase activities and antibodies against Epstein-Barr virus and Coxiella burnetii in patients with chronic fatigue syndrome in Japan. Microbes Infect. 2003;5(12):1096-102. Epub 2003/10/14. PubMed PMID: 14554250.

26. Holmes GP, Kaplan JE, Gantz NM, Komaroff AL, Schonberger LB, Straus SE, et al. Chronic fatigue syndrome: a working case definition. Ann Intern Med. 1988;108(3):387-9. PubMed PMID: 2829679.

27. Arashima Y, Kato K, Komiya T, Kumasaka K, Matsukawa Y, Murakami M, et al. Improvement of chronic nonspecific symptoms by long-term minocycline treatment in Japanese patients with Coxiella burnetii infection considered to have post-Q fever fatigue syndrome. Intern Med. 2004;43(1):49-54. Epub 2004/02/18. PubMed PMID: 14964579.

28. Thomas HV, Thomas DR, Salmon RL, Lewis G, Smith AP. Toxoplasma and coxiella infection and psychiatric morbidity: a retrospective cohort analysis. BMC Psychiatry. 2004;4:32. Epub 2004/10/20. doi: 10.1186/1471-244x-4-32. PubMed PMID: 15491496; PubMed Central PMCID: PMCPmc526777.

29. Marmion BP, Storm PA, Ayres JG, Semendric L, Mathews L, Winslow W, et al. Long-term persistence of Coxiella burnetii after acute primary Q fever. QJM. 2005;98(1):7-20. Epub 2004/12/31. doi: 10.1093/qjmed/hci009. PubMed PMID: 15625349.

30. Helbig K, Harris R, Ayres J, Dunckley H, Lloyd A, Robson J, et al. Immune response genes in the post-Q-fever fatigue syndrome, Q fever endocarditis and uncomplicated acute primary Q fever. QJM. 2005;98(8):565-74. Epub 2005/06/16. doi: 10.1093/qjmed/hci086. PubMed PMID: 15955794.

31. Iwakami E, Arashima Y, Kato K, Komiya T, Matsukawa Y, Ikeda T, et al. Treatment of chronic fatigue syndrome with antibiotics: pilot study assessing the involvement of Coxiella burnetii infection. Intern Med. 2005;44(12):1258-63. Epub 2006/01/18. PubMed PMID: 16415546.

32. Reid S, Chalder T, Cleare A, Hotopf M, Wessely S. Chronic fatigue syndrome. BMJ (Clinical Research ed). 2000;320(7230):292-6. PubMed PMID: 10650029; PubMed Central PMCID: PMC1117488.

33. Hickie I, Davenport T, Wakefield D, Vollmer-Conna U, Cameron B, Vernon SD, et al. Post-infective and chronic fatigue syndromes precipitated by viral and non-viral pathogens: prospective cohort study. BMJ (Clin Res ed). 2006;333(7568):575. Epub 2006/09/05. doi: 10.1136/bmj.38933.585764.AE. PubMed PMID: 16950834; PubMed Central PMCID: PMCPmc1569956.

34. Hadzi-Pavlovic D, Hickie IB, Wilson AJ, Davenport TA, Lloyd AR, Wakefield D. Screening for prolonged fatigue syndromes: validation of the SOFA scale. Soc Psych Psych Epid. 2000;35(10):471-9. doi: DOI 10.1007/s001270050266. PubMed PMID: WOS:000165107600006.

35. Ledina D, Bradaric N, Milas I, Ivic I, Brncic N, Kuzmicic N. Chronic fatigue syndrome after Q fever. Med Sci Monit. 2007;13(7):Cs88-92. Epub 2007/06/30. PubMed PMID: 17599032.

36. Vollmer-Conna U, Cameron B, Hadzi-Pavlovic D, Singletary K, Davenport T, Vernon S, et al. Postinfective fatigue syndrome is not associated with altered cytokine production. Clin Infect Dis. 2007;45(6):732-5. doi: <http://dx.doi.org/10.1086/520990>. PubMed PMID: 2007442464.

37. Marmion BP, Sukocheva O, Storm PA, Lockhart M, Turra M, Kok T, et al. Q fever: persistence of antigenic non-viable cell residues of Coxiella burnetii in the host--implications for post Q fever infection fatigue syndrome and other chronic sequelae. QJM. 2009;102(10):673-84. Epub 2009/06/27. doi: 10.1093/qjmed/hcp077. PubMed PMID: 19556396.

38. Zhang L, Gough J, Christmas D, Mattey DL, Richards SC, Main J, et al. Microbial infections in eight genomic subtypes of chronic fatigue syndrome/myalgic encephalomyelitis. J Clin Pathol. 2010;63(2):156-64. Epub 2009/12/04. doi: 10.1136/jcp.2009.072561. PubMed PMID: 19955554; PubMed Central PMCID: PMCPmc2921262.

39. Kerr JR, Burke B, Petty R, Gough J, Fear D, Mattey DL, et al. Seven genomic subtypes of chronic fatigue syndrome/myalgic encephalomyelitis: a detailed analysis of gene networks and clinical phenotypes. J Clin Pathol. 2008;61(6):730-9. doi: 10.1136/jcp.2007.053553. PubMed PMID: 18057078.

40. Kerr JR, Petty R, Burke B, Gough J, Fear D, Sinclair LI, et al. Gene expression subtypes in patients with chronic fatigue syndrome/myalgic encephalomyelitis. J Infect Dis. 2008;197(8):1171-84. doi: 10.1086/533453. PubMed PMID: 18462164.

41. Kaushik N, Fear D, Richards SC, McDermott CR, Nuwaysir EF, Kellam P, et al. Gene expression in peripheral blood mononuclear cells from patients with chronic fatigue syndrome. J Clin Pathol. 2005;58(8):826-32. doi: 10.1136/jcp.2005.025718. PubMed PMID: 16049284; PubMed Central PMCID: PMC1770875.

42. Kadota Y, Cooper G, Burton AR, Lemon J, Schall U, Lloyd A, et al. Autonomic hyper-vigilance in post-infective fatigue syndrome. Biol Psychol. 2010;85(1):97-103. doi: <http://dx.doi.org/10.1016/j.biopsycho.2010.05.009>. PubMed PMID: 2010421837.

43. Sukocheva OA, Marmion BP, Storm PA, Lockhart M, Turra M, Graves S. Long-term persistence after acute Q fever of non-infective Coxiella burnetii cell components, including antigens. QJM. 2010;103(11):847-63. Epub 2010/07/20. doi: 10.1093/qjmed/hcq113. PubMed PMID: 20639288.

44. Wessely S. Chronic fatigue: Symptom and syndrome. Ann Intern Med. 2001;134(9):838-43. PubMed PMID: WOS:000168543100007.

45. Limonard GJ, Peters JB, Nabuurs-Franssen MH, Weers-Pothoff G, Besselink R, Groot CA, et al. Detailed analysis of health status of Q fever patients 1 year after the first Dutch outbreak: a case-control study. QJM. 2010;103(12):953-8. Epub 2010/08/31. doi: 10.1093/qjmed/hcq144. PubMed PMID: 20802011.

46. Limonard GJ, Nabuurs-Franssen MH, Weers-Pothoff G, Wijkmans C, Besselink R, Horrevorts AM, et al. One-year follow-up of patients of the ongoing Dutch Q fever outbreak: clinical, serological and echocardiographic findings. Infection. 2010;38(6):471-7. Epub 2010/09/22. doi: 10.1007/s15010-010-0052-x. PubMed PMID: 20857313; PubMed Central PMCID: PMCPmc3003145.

47. Morroy G, Peters JB, van Nieuwenhof M, Bor HH, Hautvast JL, van der Hoek W, et al. The health status of Q-fever patients after long-term follow-up. BMC Infect Dis. 2011;11:97. Epub 2011/04/20. doi: 10.1186/1471-2334-11-97. PubMed PMID: 21501483; PubMed Central PMCID: PMCPmc3110112.

48. Wegdam-Blans MC, Nabuurs-Franssen MN, Horrevorts AM, Peeters MF, Schneeberger PM, Bijlmer HA. Laboratory diagnosis of acute Q fever [in Dutch]. Nederlands Tijdschrift voor Geneeskunde. 2010;154:A2388. PubMed PMID: Medline:20858325.

49. van Woerden HC, Healy B, Llewelyn MB, Matthews IP. A nested case control study demonstrating increased chronic fatigue six years after a Q fever outbreak. Microbiol Res. 2011;2(e19):69-72. doi: 10.4081/mr.2011.e19.

50. Galbraith S, Cameron B, Li H, Lau D, Vollmer-Conna U, Lloyd AR. Peripheral blood gene expression in postinfective fatigue syndrome following from three different triggering infections. J Infect Dis. 2011;204(10):1632-40. Epub 2011/10/04. doi: 10.1093/infdis/jir612. PubMed PMID: 21964398.

51. Piraino B, Vollmer-Conna U, Lloyd AR. Genetic associations of fatigue and other symptom domains of the acute sickness response to infection. Brain Behav Immun. 2012;26(4):552-8. Epub 2012/01/10. doi: 10.1016/j.bbi.2011.12.009. PubMed PMID: 22227623.

52. Strauss B, Loschau M, Seidel T, Stallmach A, Thomas A. Are fatigue symptoms and chronic fatigue syndrome following Q fever infection related to psychosocial variables? J Psychosom Res. 2012;72(4):300-4. Epub 2012/03/13. doi: 10.1016/j.jpsychores.2012.01.010. PubMed PMID: 22405225.

53. Gilsdorf A, Kroh C, Grimm S, Jensen E, Wagner-Wiening C, Alpers K. Large Q fever outbreak due to sheep farming near residential areas, Germany, 2005. Epidemiol Infect. 2008;136(8):1084-7. PubMed PMID: Medline:17892631.

54. Morroy G, Bor HH, Polder J, Hautvast JL, van der Hoek W, Schneeberger PM, et al. Self-reported sick leave and long-term health symptoms of Q-fever patients. Eur J Public Health. 2012;22(6):814-9. Epub 2012/02/09. doi: 10.1093/eurpub/cks003. PubMed PMID: 22315459.

55. Arashima Y, Yakubo S, Nagaoka H, Komiya T, Murakami M, Nakayama T, et al. A patient in whom treatment for coxiella burnetii infection ameliorated a depressive state and thoughts of impending death. International Medical Journal. 2012;19(1):65-6. PubMed PMID: 2012223501.

56. Raoult D. Chronic Q fever: expert opinion versus literature analysis and consensus. J Infect. 2012;65(2):102-8. Epub 2012/04/28. doi: 10.1016/j.jinf.2012.04.006. PubMed PMID: 22537659.

57. Hussain-Yusuf H, Islam A, Healy B, Lockhart M, Nguyen C, Sukocheva O, et al. An analysis of Q fever patients 6 years after an outbreak in Newport, Wales, UK. QJM. 2012;105(11):1067-73. Epub 2012/07/10. doi: 10.1093/qjmed/hcs119. PubMed PMID: 22771556.

58. van Woerden HC, Mason BW, Nehaul LK, Smith R, Salmon RL, Healy B, et al. Q fever outbreak in industrial setting. Emerg Infect Dis. 2004;10(7):1282-9. doi: 10.3201/eid1007.030536. PubMed PMID: 15324550; PubMed Central PMCID: PMC3323322.

59. Oosterheert JJ, Kampschreur L, Hoepelman AI. [Fatigue after Q fever: nothing new]. Nederlands Tijdschrift voor Geneeskunde. 2012;156(48):A5474. Epub 2012/11/30. PubMed PMID: 23191975.

60. Yakubo S, Ueda Y, Tanekura N, Arashima Y, Nakayama T, Komiya T, et al. The first case of a patient suffering from Coxiella burnetii infection attempting suicide arising from a state of depression. International Medical Journal. 2012;19(4):312-3. PubMed PMID: 2012700928.

61. Keijmel SP, Delsing CE, Sprong T, Bleijenberg G, van der Meer JW, Knoop H, et al. The Qure study: Q fever fatigue syndrome--response to treatment; a randomized placebo-controlled trial. BMC Infect Dis. 2013;13:157. Epub 2013/03/30. doi: 10.1186/1471-2334-13-157. PubMed PMID: 23536997; PubMed Central PMCID: PMCPmc3620935.

62. National Institute for Public Health and the Environment. Dutch guideline Q fever fatigue syndrome (QFS) [in Dutch] 2012. Available from: <http://www.rivm.nl/dsresource?objectid=rivmp:118226&type=org&disposition=inline>.

63. Wegdam-Blans M, Kampschreur L, Delsing C, Bleeker-Rovers C, Sprong T, van Kasteren M, et al. Chronic Q fever: review of the literature and a proposal of new diagnostic criteria. J Infect. 2012;64:247 - 59. PubMed PMID: doi:10.1016/j.jinf.2011.12.014.

64. Yakubo S, Yakubo S, Ueda Y, Tanekura N, Arashima Y, Munemura T, et al. Kampo Formula Shakuyaku-kanzo-To Alleviates Sensation of Muscle Spasm in Coxiella burnetii Infection. International Medical Journal. 2013;20(2):218-20. PubMed PMID: 2012154829.

65. van Asseldonk MA, Prins J, Bergevoet RH. Economic assessment of Q fever in the Netherlands. Prev Vet Med. 2013;112(1-2):27-34. Epub 2013/07/23. doi: 10.1016/j.prevetmed.2013.06.002. PubMed PMID: 23866818.

66. National Institute for Public Health and the Environment. Available from: <http://www.rivm.nl/Onderwerpen/Q/Q_koorts>.

67. van der Hoek W, Dijkstra F, Schimmer B, Schneeberger PM, Vellema P, Wijkmans C, et al. Q fever in the Netherlands: an update on the epidemiology and control measures. Eurosurveillance. 2010;15(12). PubMed PMID: 20350500.

68. van Loenhout JA, Paget WJ, Sandker GW, Hautvast JL, van der Velden K, Vercoulen JH. Assessing health status and quality of life of Q-fever patients: The Nijmegen Clinical Screening Instrument versus the Short Form 36. Health Qual Life Outcomes. 2013;11(1). doi: <http://dx.doi.org/10.1186/1477-7525-11-112>.

69. Dijkstra F, van der Hoek W, Wijers N, Schimmer B, Rietveld A, Wijkmans CJ, et al. The 2007-2010 Q fever epidemic in The Netherlands: characteristics of notified acute Q fever patients and the association with dairy goat farming. FEMS Immunol Med Microbiol. 2012;64(1):3-12. doi: 10.1111/j.1574-695X.2011.00876.x. PubMed PMID: 22066649.

70. Yakubo S, Ueda Y, Arashima Y. Long-term absence from school of a boy suffering severe general Malaise from coxiella burnetii infection. International Medical Journal. 2013;20(6):688-90. PubMed PMID: 2014054222.

71. Brooke RJ, van Lier A, Donker GA, W VDH, Kretzschmar ME. Comparing the impact of two concurrent infectious disease outbreaks on The Netherlands population, 2009, using disability-adjusted life years. Epidemiol Infect. 2014:1-10. Epub 2014/01/31. doi: 10.1017/s0950268813003531. PubMed PMID: 24476696.

72. Arashima Y, Yakubo S, Ueda Y, Munemura T, Komiya T, Isa H, et al. A first case of asthma thought to be caused by coxiella burnetti infection. International Medical Journal. 2013;20(6):699-700. PubMed PMID: 2014054225.

73. Kremers MN, Janssen R, Wielders CC, Kampschreur LM, Schneeberger PM, Netten PM, et al. Correlations between peripheral blood coxiella burnetii DNA load, interleukin-6 levels, and C-reactive protein levels in patients with acute Q fever. Clin Vaccine Immunol. 2014;21(4):484-7. doi: <http://dx.doi.org/10.1128/CVI.00715-13>. PubMed PMID: 2014239787.

74. van Loenhout JA, van Tiel HH, van den Heuvel J, Vercoulen JH, Bor H, van der Velden K, et al. Serious long-term health consequences of Q-fever and Legionnaires' disease. J Infect. 2014. Epub 2014/01/29. doi: 10.1016/j.jinf.2014.01.004. PubMed PMID: 24468188.

75. van Loenhout JA, Paget WJ, Vercoulen JH, Wijkmans CJ, Hautvast JL, van der Velden K. Assessing the long-term health impact of Q-fever in the Netherlands: a prospective cohort study started in 2007 on the largest documented Q-fever outbreak to date. BMC Infect Dis. 2012;12(280). doi: <http://dx.doi.org/10.1186/1471-2334-12-280>. PubMed PMID: 2012706082.

76. van Dam S, van Loenhout JA, Peters JB, Rietveld A, Paget WJ, Akkermans RP, et al. A cross-sectional study to assess the long-term health status of patients with lower respiratory tract infections, including Q fever. Epidemiol Infect. 2014:1-7. Epub 2014/03/15. doi: 10.1017/s0950268814000417. PubMed PMID: 24625631.

77. van Loenhout JA, Hautvast JL, Vercoulen JH, Akkermans RP, Wijkmans CJ, van der Velden K, et al. Q-fever patients suffer from impaired health status long after the acute phase of the illness: results from a 24-month cohort study. J Infect. 2015;70(3):237-46. doi: 10.1016/j.jinf.2014.10.010. PubMed PMID: 25452036.

78. van Loenhout JA, Wielders CC, Morroy G, Cox MJ, van der Hoek W, Hautvast JL, et al. Severely impaired health status of non-notified Q fever patients leads to an underestimation of the true burden of disease. Epidemiol Infect. 2015:1-8. Epub 2015/01/15. doi: 10.1017/s0950268814003689. PubMed PMID: 25582890.

79. European Union. Amending Decision 2002/253/EC laying down case definitions for reporting communicable disease s to the Community network under Decision No. 2119/98/EC of the European Parliament and of the Council. Official Journal of the European Union. 2008.

80. van Loenhout JA, Hautvast JL, Akkermans RP, Donders NC, Vercoulen JH, Paget WJ, et al. Work participation in Q-fever patients and patients with Legionnaires' disease: A 12-month cohort study. Scand J Public Health. 2015;43(3):294-301. doi: 10.1177/1403494815571030. PubMed PMID: 25724468.
