# Supplementary material for: Fatigue following Acute Q-Fever: A Systematic Literature Review
Source: PLoS One. 2016 May 25;11(5):e0155884. doi: 10.1371/journal.pone.0155884 (PMC4880326; doi:10.1371/journal.pone.0155884)
Supplement: S2 Table — (DOCX) [file pone.0155884.s003.docx]

**S2 Table. Domain diagnosis**

| **Ref** | **Country, yr study, period and duration** | **Study type** | **Patients, controls, characteristics, co-morbidity*** | **Tool** | **Inter-ven-tion** | **Outcome** | **Conclusions/recommendations** | **Other do-main** | **QA (NOS)** | | |
| --- | --- | --- | --- | --- | --- | --- | --- | --- | --- | --- | --- |
| 1999, J. Scadding [1] | Country unknown, 1999. Duration study NA | PO, comment on [2] | No patients/controls. Characteristics and co-morbidity: NR | NA | NA | CFS, defined in clinical-descriptive terms, should convey no causal implication; when there is convincing evidence of a causal factor, the case belongs to a causally-defined subset of this syndrome. PQFS conforms to this desideratum | If mechanisms of complaints and specific therapeutic approaches are unknown, the term PQFS/QFS should be used as this leaves no doubt that findings are relevant to a CFS subset | NA | NA | | |
| 2012, D. Raoult [3] | France, 2012 | PO, comment on [4] | No patients/controls. Characteristics and co-morbidity: NR. Focus on CQF | NA | NA | NA | CF is a non-specific subjective state, not a specific symptom of QF; no treatment is currently effective, it is not a diagnostic problem. Some patients with fatigue have high antibody titres, others not | NA | NA | | |
| 2012, J. Ooster-heert [5] | Netherlands, 2012 | PO, comment on [6] | No patients/controls. Characteristics and co-morbidity: NR. Focus on terminology of fatigue following QF | NA | NA | NA | Important to underline and recognise PIFS. New terminology QFS not useful; PIF described for many infectious diseases; not causative micro-organism, but disease severity correlates with symptom duration post AI. Can lead to cultivation, attracting patients with other intentions then getting better, ↑ healthcare costs | NA | NA | | |
| 2013, J. van Loen-hout [7] | Netherlands, 2011-2012, single mea-surement 12 mo post illness onset | CoS | 309 AQF patients, no controls. To assess use of NCSI and SF-36 in providing a detailed assessment of health status of QF patients and to evaluate which subdomains measure unique aspects of health status | NCSI, SF-36 | NA | NCSI: ↓ intercorrelations subdomains. 4 subdomains showed conceptual similarity (Subjective Pulmonary Symptoms, Subjective Impairment and Dyspnoea Emotions, and between Fatigue and Health Related Quality of Life) with ≥1 SF-36 subdomain (Vitality and General Health, and between Vitality and Mental Health and Social Functioning) and vice versa | Both NCSI and SF-36 can be used to measure health status in QF patients. Combining NCSI and 4 SF-36 subdomains (Role Physical, Bodily Pain, Social Functioning, Role Emotional), is preferred to obtain a detailed overview | NA | ★★  ✰  ✰ | ✰  ✰ | ★  ✰★ |

**** Definition of used study population in articles explained in a different table, including definitions of QFS and/or fatigue is applicable. Main information is on diagnosis. Some articles also contain relevant information on other domains: A= Aetiology, B/D= Background/descriptive, P/T= Prevention/therapy***

***Abbreviations:*** AI= Acute infection, AQF= Acute Q-fever, CF= Chronic fatigue, CFS= Chronic fatigue syndrome, CoS= Cohort study, CQF= Chronic Q-fever, Mo= Month(s), NA= Not applicable, NCSI= Nijmegen clinical screening instrument, originally developed to provide a detailed assessment of health status of COPD patients. It combines a number of existing health status questionnaires, NOS= Newcastle–Ottawa Scale: S= selection (maximum of 4 stars), C= comparability (maximum of 2 stars), O= outcome (maximum of 3 stars); ★: star earned; ☆: item not applicable, NR= Not reported, PIF(S)= Post-infective fatigue (syndrome), PO= Personal opinion, PQFS= Post-(acute)Q-fever (fatigue) syndrome, QA= Quality assessment, QF= Q-fever, QF(F)S= Q-fever fatigue syndrome, Ref= Reference, SF-36= The Short Form (36) Health Survey, a patient-reported survey of patient health to assess quality of life of patients, functional impairment and reduced health related quality of life, Yr(s)= Year(s)

**References**

1. Scadding JG. Fatigue syndromes. QJM. 1999;92(5):293-4. Epub 2000/01/01. PubMed PMID: 10615486.

2. Penttila IA, Harris RJ, Storm P, Haynes D, Worswick DA, Marmion BP. Cytokine dysregulation in the post-Q-fever fatigue syndrome. QJM. 1998;91(8):549-60. Epub 1999/01/20. PubMed PMID: 9893758.

3. Raoult D. Chronic Q fever: expert opinion versus literature analysis and consensus. J Infect. 2012;65(2):102-8. Epub 2012/04/28. doi: 10.1016/j.jinf.2012.04.006. PubMed PMID: 22537659.

4. Wegdam-Blans M, Kampschreur L, Delsing C, Bleeker-Rovers C, Sprong T, van Kasteren M, et al. Chronic Q fever: review of the literature and a proposal of new diagnostic criteria. J Infect. 2012;64:247 - 59. PubMed PMID: doi:10.1016/j.jinf.2011.12.014.

5. Oosterheert JJ, Kampschreur L, Hoepelman AI. [Fatigue after Q fever: nothing new]. Nederlands Tijdschrift voor Geneeskunde. 2012;156(48):A5474. Epub 2012/11/30. PubMed PMID: 23191975.

6. Keijmel SP, Morroy G, Delsing CE, Bleijenberg G, Bleeker-Rovers CP, Timen A. [Persistent fatigue following Q fever]. Nederlands Tijdschrift voor Geneeskunde. 2012;156(48):A5258. Epub 2012/11/30. PubMed PMID: 23191971.

7. van Loenhout JA, Paget WJ, Sandker GW, Hautvast JL, van der Velden K, Vercoulen JH. Assessing health status and quality of life of Q-fever patients: The Nijmegen Clinical Screening Instrument versus the Short Form 36. Health Qual Life Outcomes. 2013;11(1). doi: <http://dx.doi.org/10.1186/1477-7525-11-112>.
