# Supplementary material for: Fatigue following Acute Q-Fever: A Systematic Literature Review
Source: PLoS One. 2016 May 25;11(5):e0155884. doi: 10.1371/journal.pone.0155884 (PMC4880326; doi:10.1371/journal.pone.0155884)
Supplement: S3 Table — (DOCX) [file pone.0155884.s004.docx]

**S3 Table. Domain background/descriptive**

| **Ref** | **Country, yr study, period and duration** | **Study type** | **Patients, controls, characteristics, co-morbidity*** | **Tool** | **Inter-ven-tion** | **Outcome** | **Conclusions/recommendations** | **Other do-main** | **QA (CR or NOS)** | | |
| --- | --- | --- | --- | --- | --- | --- | --- | --- | --- | --- | --- |
| 1960, O. Powell [1] | Australia, 1958 July-1959 June | Observa-tional pros. CoS | AQF patients (n=72, all ♂), describe clinical features and FU | NR | NA | Proportion of cases convalescence prolonged, with undue fatigue, setback up on moderate exertion, poor appetite, and occasional headache. 15/61 returned to work >6 wks post AQF, 12 >8wks. Mean period off work: 0-29 yrs 29 days, 30-49 yrs 45 days, 50-69 yrs 68 days. Total amount of time on workers’ compensation payment 2013 days | Confirms previous observations that convalescence is more protracted in elderly | NA | ★  ★  ★ |  |  |
| 1990, S. Reilly [2] | UK, 1972-1988, study period 16 yrs | Observa-tional pros. CoS | Seroprevalence *C.b.* assessed after testing all FUO, respiratory infections, CNE, and hepatitis cases. Co-morbidity: NR. Time baseline (AQF) to measurement complaints NR | CFT, IFA (selected cases) | NA | 103 *C.b.* infections: 46 AQF, 5 CQF, 52 past infections. Details 61 cases (46 AQF, 5 CQF, 10 past infections). Outcome AQF: 57% uncomplicated, 4% prolonged fatigue (duration unknown), 11% underlying malignancy, 9% neurological sequelae, 9% persistent chest symptoms, 9% hepatic dysfunction. Outcome previous infection: 10% prolonged fatigue, 10% depression, 10% lymphadenopathy, 20% sarcoidosis, 10% polyarthritis nodosa | QF remains unpredictable, with a propensity to follow a protracted course. Prolonged serological and clinical surveillance of all QF cases is suggested | NA | ★  ✰★ |  | ★  ★ |
| 1995, P. Harvey-Sutton [3] | Australia, yr NR | POB | N unknown. PQDS or PQCFS. No control group | NA | NA | Observation of bradycardia in PQDS patients | Bradycardia may be a sign of PQDS | NA | NA | | |
| 1996, B. Marmion [4] | Australia, 1995. Study period: 5-14 yrs post AQF in 1981-89 | CC | Post AQF laboratory proven (n=39) with QFS, skin-test /antibody negative vaccinated (n=39), skin-test or antibody positive without QF history (39), seronegative (n=39). Controls matched (sex, ≤10 yrs age). Co-morbidity: NR | 54-item questionnaire based on symptoms | NA | Combinations fatigue, night sweats, myalgia, fasciculation, with various minor symptoms more common in post AQF group, in 18-48% depending on number and mix of symptoms used for QFS definition. Met CFS CDC criteria: 11/39 post AQF, 0/39 vaccinees, 0/39 other controls | Interpretations range from compensation-driven through psychogenic perpetuation of original symptoms/depression, to chronic immune stimulation. Hypothesis persistence *C.b./*its antigens causes dysregulation macrophage/T-lymphocyte axis with aberrant monokine and lymphokine production mediating symptoms | Diag,  A | ★  ★  ★ | ★  ★ | ★  ★ |
| 1996, J. Ayres [5] | UK, 1995. Study period 6 yrs post AQF | CC | QF patients (n=83, 70 ♂) vs. matched (age, sex) controls (n=26). Co-morbidity: NR. Assess prevalence chronic symptoms 6 yrs post AQF | Questionnaire as in [4] | NA | QF group: 66% fatigue, 69% joint aches, 65% sleep disturbance, 59% cough, 53% sweats, irritability 54%, chest pain 51%, breathlessness 49%, headaches 47%, dizziness 39%, blurred vision 34%, alcohol intolerance 33%. ↑ Prevalence cases i.c.w. controls: joint pains, sleep disturbance, cough, sweats, irritability, chest pain, breathlessness, dizziness. No difference prevalence fatigue, blurred vision, headaches, alcohol intolerance | Findings support view that chronic PQFS exists which is in many ways similar to CFS | NA | ★  ★  ★ | ★  ★ | ★  ✰ |
| 1998, J. Ayres [6] | UK, 1994. Study period: 5 yrs post AQF | CC | 71 symptomatic *C.b.* (mean age 55, 81.7% ♂, 32.4% current smokers). Matched (sex, age, ethnicity) controls: 142 (55 yrs, 81.7% ♂, 16.9% no febrile illness needing medical attention April-July 1989). Asses CFS symptoms prevalence post AQF | Modified questionnaire [4], including VAS per symptom | NA | QF symptom prevalence: significant ↑ fatigue, sweating, blurred vision, breathlessness on exertion (especially non-smokers) than controls. QF cases symptom severity: ↑ fatigue, blurred vision, sweating, memory ↓, joint pains and headaches. 42.3% QF cases and 26% controls had CFS according to CDC criteria (p=0.025, post-hoc) | A syndrome characterized by undue fatigue, breathlessness on exertion, excessive sweating and blurred vision post *C.b.* infection, persists yrs. Defining questionnaire based syndrome due to QF is dangerous, objective measures needed. Mechanism elusive, subclinical cardiomyopathy/autonomic dysfunction suggested | NA | ★  ★  ★  ★ | ★  ★ | ★ |
| 1998, K. Kato [7] | Japan, March 1996-April 1997. Period: NA. Single blood samples | CC | 52 patients (13 ♂, mean age 41 SD15, range 9-74): fatigue 77%, feeling feverish 44%, joint aches/ myalgia 70%, headache 56%, cough/sore throat 42%; duration 4.9 yrs SD1.0, range 0.5-22. 52 healthy controls (35 ♂, mean age 52, SD10, range 38-82), and 70 cord blood samples | n-PCR | NA | Physical examination. CFS: 17/52 *C.b.* positive, amplification 438-bp fragments n-PCR. 52 controls 5/52 and 2/70 cord blood samples positive n-PCR. Mean age patients positive n-PCR 42, SD14, range 9-67. Estimated duration fatigue 77%, feeling feverish 53%, joint aches/myalgia 70%, headache 41%, cough/sore throat 47% was 4.0 yrs SD1.2. Positive ratio patients nonspecific complaints ↑ i.c.w. healthy controls (p<0.05) and cord blood (p<0.001) | High prevalence *C.b.* infection adult patients with long term, nonspecific complaints i.c.w. healthy controls, and possible existence chronic post AQF syndrome in Japan. Results appear to support the report of [4] and QFS concept | NA | ★  ★ |  | ★  ★ |
| 2002, M. Wildman [8] | UK, 1999 | CC | 10 yrs post *C.b.* outbreak. 80 matched controls (sex, age, and smoking) random 2 local general practitioners (mean age 55.4, SD11.7, 68 ♂). 108 Q-exposed cases (mean age 55.6, SD11.8, 68 ♂) last contacted 1989/1994. Exclusion controls serology positive *C.b*. 77 matched pairs analysed. Aim: had subjects involved in West Midlands 1989 outbreak ↑ fatigue i.c.w. non-exposed controls 10 yrs later | 11‐item fatigue questionnaire, GHQ, CIS-R. MOS, SDQ. Laboratory test *C.b.*, spirome-try, ECG, shuttle walk, incremen-tal exercise test | NA | 108 Q‐exposed, 64.8% fatigue, 34.3% ICF vs. controls 36.3% and 15.0%. 77 matched pairs: fatigue Q‐exposed vs. controls: 64.9% vs. 35.1%, p<0.0001. ICF in 32.5% Q‐exposed and 14.3% controls, p=0.01. 46.8% GHQ cases Q‐exposed vs. 23.4% controls, p=0.004. Matched analysis: fatigue 66.7% Q‐exposed, 34.7% controls, p<0.001, ICF 34.7% Q‐exposed vs. 13.9% controls, p=0.004. CFS 19.4% Q‐exposed vs. 4.2% controls. p=0.003. 47.2% Q‐exposed had GHQ vs. 23.6% controls, p=0.004 | *C.b.* cases exposed in 1989 had more fatigue than controls, some fulfilled CFS criteria. Uncertain if this is due to ongoing antigen persistence or to psychological effects of prolonged medical follow‐up | A | ★  ★  ★  ★ | ★  ★ | ★  ★ |
| 2002, B. Marmion [9] | Australia, 2002. Duration study NA | PO, comment on [10] | No patients/controls. Characteristics and co-morbidity: NR | NA | NA | Previous report [11] did not claim persistent infection to cause PQFS. Substantial proportion AQF patients have QFS-like symptoms to QFS (milder version of acute phase symptoms without fever) for 6-9 mo post AQF and then recover. ±8-10% exhibit similar symptoms and do not reach immune/other homeostasis ≥1 yr | Systematic FU AQF patients needed, as 8-10% not recover ≥2 yrs post AQF | NA | NA | | |
| 2002, M. Wildman [12] | UK, 2002. Duration study NA | PO, comment on [10] | No patients/controls. Characteristics and co-morbidity: NR | CFS study group’s 1994 CDC definition; 3 fatigue levels with ↑ seve-rity: (i) fatigue, (ii) idiopathic CF, and (iii) CFS | NA | Findings in fatigue prevalence study [8] differs from 5-10% found by others [10]. However, prevalence of fatigue in UK’s general practice population is 38% vs. 36.3% in controls [8]. Idiopathic CF: 18.3% general practice vs. 15% in [8] | Lack explicit measurement instruments make comparison fatigue between studies impossible. Increased fatigue scores in QF exposed cohort were measured with standardized and well-validated instru-ments, permitting replication. Fatigue measurement is essential and should be standardized to compare studies | NA | NA | | |
| 2003, T. Hatchette [13] | Canada, yr study NR, study period: 1999-2001 | CoS | Post AQF (n=33), controls without AQF during same outbreak cohort (n=24). Characteristics and co-morbidity NR. To follow effect of AQF on quality of life of patients 3 and 27 mo post AQF | Questionnaires on nature and duration of symptoms, SF-36 | NA | 3 mo post AQF only General Health scores of *C.b.* infected were ↓ than controls (p=0.03). 27 mo post AQF scores 5/8 domains and physical/mental summary scales ↓ i.c.w. controls. 27 mo post AQF 52% *C.b.* infected still reported symptoms, incl. 7 with initially resolved symptoms 3 mo post AQF. Of 3 *C.b.* infected symptoms resolved at 27 mo, who initially had persistent symptoms. ↓ scores General Health, Mental Health, Vitality and physical summary scales in those with persistent symptoms i.c.w. no symptoms. No initial symptoms nor antibiotic treatment of AQF predictive for developing persistent symptoms post AQF | Post *C.b.* infection symptoms can persist >2 yrs with significant quality of life impact. Data reflect further evidence of QFFS. Differences may reflect socioeconomic, physiological/ psychological effects of being labelled with QF rather than true post-infectious sequelae | A | ★  ★  ★  ★ |  | ★  ★ |
| 2004, H. Thomas [14] | UK, 1999. Study period March-July 1999 | CC | Random sample farmers (n= 425). Test seroprevalence *T. gondii* and *C.b.,* and association *T. gondii*, slow reaction and poor concentration, and between *C.b.* and persistent fatigue, and association organisms with depression/ depressive ideas. Duration infection unknown | CIS-R, venous blood | NA | 15% relevant fatigue levels, 5% concentration problems, 5% depressive ideas, 4% depression, 6% general psychiatric morbidity. Seroprevalence: 45% *T. gondii*, ↑ with age, no gender differences; 31% *C.b.*, no association age/gender. 46 seropositive for both. Neither infection associated clinical relevant fatigue, concentration problems, depression, depressive ideas/overall psychiatric morbidity i.c.w. seronegative individuals, not associated ↑ risk psychiatric outcome after age and sex adjustment. ↑ % *C.b.* seronegative psychiatric symptoms i.c.w. seropositive | No evidence *T. gondii/C.b.* infections associated with neuropsychiatric morbidity, in particular poor concentration/fatigue | NA | ★  ★  ★ |  | ★  ★ |
| 2006, I. Hickie [15] | Australia, (sub study DIOS), yr study NR | CoS | N=253; 68 EBV (mean age 22, range 16-49, 57% ♀), 60 RRV (mean age 40, range 18-69, 45% ♀), 43 *C.b.* (mean age 40, range 16-73, 14% ♀), 82 not confirmed (mean age 38, range 16-77, 44% ♀). Control of fatigue: baseline, 3 and 6 wks, 3 and 12 mo post AI. Excluded; hypothyroidism/primary sleep-/psychiatric disorders. Controls (age and sex matched) recovered from AI at 6 mo | SPHERE, SOMA Laboratory and clinical examination | NA | Provisional PIFS case rate 35% at 6 wks, 27% at 3 mo, 12% at 6 mo, and 9% at 12 mo, regardless of the infective agent, age, gender or psychiatric disorders. Confirmed PIFS: 28 cases (14 ♂, 14 ♀, mean age 37, range 17-63); 5 EBV, 3 QF, 13 RRV, 8 unconfirmed infections. I.c.w. all participant, no difference in age/sex. I.c.w. controls, comparable: premorbid psychiatric diagnosis, intercurrent psychiatric disorders. Confirmed PIFS: median score acute sickness factor rapidly ↓ to zero, for fatigue, musculoskeletal pain and neurocognitive disturbance remained ↑ | Pro-inflammatory cytokines do not remain ↑ in PIFS. Key risk factor PIFS is severity acute illness; not demographic, psychological (premorbid/intercurrent psychiatric disorders) factors | A | ★  ★★  ★ | ★  ★ | ★ |
| 2010, G. Limonard [16] | Netherlands, 2008 | CC | 54 post AQF patients (61.1% ♂, mean age 53.1, SD14.2, co-morbidity 40.7%, current smoker 44.4%). 23 seronegative neighbourhood controls (sex matched, age ±10 yrs) (42.3% ♂, mean age 53.6, SD9.7, co-morbidity 39.1%, current smoker 26.1). Asses health status 1 yr post AQF | NCSI | NA | *C.b.* cases scored 1 yr post AQF significantly worse for all subdomains of symptoms. 52% cases clinically significant fatigue vs. 26% controls. Abnormal fatigue score QF patients 74% vs. controls 48%. Severe levels resp. 52% vs. 26%. NCSI scores of 11 seropositive and 23 seronegative controls not different for 8 subdomains health status | Sustained ↓ in health status 1 yr post AQF. NCSI scores from seropositive controls without clinical QF history comparable with seronegative controls, suggesting that clinical expression of AQF is essential in subsequent sustained ↓ health status | A | ★  ★  ★  ★ | ★  ★ | ★  ★ |
| 2010, G. Limonard [17] | Netherlands, yr study NR. Study period 2007-2008 | CoS | 85 AQF patients (62% ♂, mean age 49 (18-80)). No controls. Co-morbidity: n=26 (6 cardiovascular, 3 pulmonary, 1 neurological, 4 rheumatological, 1 haematological, 3 depression, 5 diabetes, 3 other). Hospitalisation: 24 AQF patients | Post AQF: history, physical examination (6, 12 mo), IFA, CFT (baseline, 3, 6, 12 mo). Single transthoracic echocardiography | NA | Post AQF 59% persistent symptoms at 6 mo and 30% at 12 mo FU. Self-reported fatigue initially 69%, at 6 mo 52%, at 12 mo 26%. No CQF. 59% had cardiac valvulopathy. ↑ antibody titres up to 3 mo, and ↓ in the following 9 mo | Screening echocardiography is no longer standard post AQF. At 6 mo fatigue is the most common complaint. Further studies needed with a control group to assess health status | NA | ★  ✰★  ★ | ✰✰ | ★  ★ |
| 2011, G. Morroy [18] | Netherlands, study period 2008-2011 | CC | 515 notified QF patients (2007 and 2008) with known 1^st^ day of illness (mean age 50.4 and 51.8 yrs, 60% ♂, 57.2 % co-morbidity) vs. healthy individuals (n=65) and severe COPD patients (n=128) assessed 12-26 mo post AQF | NCSI | NA | Abnormal fatigue score 58.9% QF patients, of which 43.5% severe. Similar scores for participants older and younger than 50 yrs. I.c.w. healthy controls (12.3% fatigue) QF patients scored significantly worse but better than COPD controls for subdomain fatigue. Hospitalisation, heart and lung disease, arthritis and depression significantly influence degree of fatigue | Sustained ↓ in health status 12-26 mo post AQF regardless of age. Policy makers ought to take this into account when considering measures to curb the extensive outbreak. Hospitalisation and co-morbidity predictors ↓ health status. More attention needed prevention and treatment long-term consequences | A | ★  ★  ★ | ★  ★ | ★  ★ |
| 2011, HC van Woerden [19] | UK, yr study 2008 | Nested-CC | 32 post AQF 6 yrs post outbreak Newport Wales, 2002 (mean age 50.18, SD 9.85). 13 controls (mean age 53.57, SD 8.86). Assess if i) CF ii) depression, and iii) ↓ physical functioning were more common in AQF patients 2002 i.c.w. controls | *C.b.* IFA, PHQ-9, Chalder Fatigue scale, GHQ | NA | Chalder Fatigue scores cases significantly ↑ (P=0.047). PHQ-9 and GHQ scores equal i.c.w. controls. CS analysis relationship IgGII in 2008 and Chalder Fatigue scores (P=0.004) and PHQ-9 scores (0.049). Longitudinal association AQF and CF 6 yrs later. CS analysis relationship depression scores (PHQ-9) and positive QF serology | CF more common 6 yrs later in QF positive patients. Possible relationship ↑ *C.b.* IgGII, symptoms CF and depression. High antibody levels may indicate ↑ responder status rather than presence micro-organism. Points up the desirability trial antibiotic treatment in QFS | P/T | ★  ★  ★  ★ |  | ★  ★ |
| 2012, B. Strauss [20] | Germany, yr study NR | CC | 84 post *C.b.* 2 yrs post Jena outbreak 2005 (mean age 48.4, SD15.2, ♀ 49%). 85 controls (mean age 49.3, SD16.8, ♀ 61% same general practitioner not controlled *C.b.)*. To investigate if fatigue/CF and/or CFS more frequent in *C.b.* infected vs. non-infected controls, and contrast QF patients with /without fatigue symptoms related to somatoform symptoms, hypochondrial worries/beliefs, psychosocial complaints and social support | MFI 20, SF-12, CDC-SI, SOMS, WI, OQ-45, F-Sozu K14, mini-DIPS | NA | Post *C.b.* more fatigue symptoms and CF i.c.w. controls (54.8 vs. 20%, 32.1 vs. 4.7%). Not more CFS criteria (1 patient each group). *C.b.* with fatigue symptoms had significantly ↑ scores SOMS, WI, ↑ psychosocial complaints with OQ-45. Health Related Quality of Life QF group ↓ than controls | Fatigue symptoms common among QF patients. No ↑ CFS prevalence among QF patients. Combination fatigue and other psychosocial symptoms support biopsychological aetiology. CBT might be optional for prolonged fatigue post QF for those with psychological distress | A, P/T | ★  ★  ★ | ★  ★ | ★ |
| 2012, G. Morroy [21] | Netherlands, yr study: 2008-2011, duration: 2007-2010 | CoS | 515 notified AQF, known 1^st^ day of illness in 2007 or 2008 (mean age resp. 50.4 and 51.8; 60% ♂, 57.2% with co-morbidity) FU 12 or 26 mo post AQF. Quantification of sick leave post AQF and long-term symptoms | NCSI and open questions regarding work | NA | Post AQF 39.6% more 1 mo absent work. Hospitalisation during AQF, smoking and heart disease independent risk factors for long-term sick leave. At 12-26 mo post AQF 9.3% unable to function at pre QF levels due to fatigue and ↓ concentration. >30% not fully resumed daily activities; 80.8% due to fatigue, 4.9% due to respiratory problems. 12-26 mo post AQF 40% reported health complaints; fatigue 19.8%, difficulty concentrating 9.5%, muscle pain 9.0%, night sweats 7.9%, eye problems 3.8% | QF has considerable impact on productivity and perceived health status. Hospitalisation, indicator of AQF severity, was a predictor for long-term sick leave and fatigue | NA | ★  ✰★ | ✰  ✰ | ★★ |
| 2012, Y. Arashima [22] | Japan, yr study NR | CR | ♂ 46 yrs, general fatigue, slightly elevated body temperature, night sweats, noise in ears, taste disturbance, headache, cough. Result: depressed with thoughts of death. Disease period 3 mo earlier. Co-morbidity: high-level depression (SDS 65) after start symptoms. PS 6. IgMI, IgMII, and IgGI negative, IgGII 1:64, n-PCR serum positive | PS, SDS, n-PCR, IFA | Minocycline 1 mo 200 mg/d, switched to 100mg/d (to-tal 3 mo). Antidepres-sant p.o. | <1-2 weeks treatment, arthralgia and slightly elevated body temperature ↓, other symptoms improved. At completion, clinical symptoms almost resolved. IgMI, IgMII, IgGI, IgGII all negative, n-PCR negative. PS 1. SDS 47. 1 yr FU: no exacerbations | PQFS is associated with depression. Minocycline seems effective. Carefully monitor depression in PQFS | Diag, A, P/T | -/-, +, ++, ++, ++, NA, +/-, -/- | | |
| 2012, S. Yakubo [23] | Japan, yr study NR | CR | ♂ 53 yrs, past QF infection, general fatigue, nausea, stomach pain, abnormal oral sensation, sore throat, trouble sleeping. Co-morbidity: depression | SDS | Antide-pres-sant, *C.b.* antibi-otic | Depression triggered by *C.b.* led to suicide | Treat *C.b.* with antibiotic. Check for depression, if present treat aggressive. Consider psychiatrist early. SDS is useful in these cases | Diag P/T | -/-, +/-, +/-, +/-, +/-, NA -,  -/- | | |
| 2013, M. van As-seldonk [24] | Netherlands, 2012. Study period: 2007-2011 | Economic evalua-tion | No patients/controls. Co-morbidity and characteristics: NR. Assess economic impact QF outbreak in the Netherlands, clarify costs-benefits control campaign, quantify and compare costs livestock sector, human health costs and disease burden. ±25% post AQF who seek medical attention expected to have CFS. Recovery period CFS 5-10 yrs (calculated with 7.5 yrs, working 50% contract time). Disability rate/weight factor: 0.14. 3.000 Euro/notified case | DALY (YLD, YLL). Deterministic socio-economic model | NA | Total disease burden 2462 DALY, of which CFS 1481 DALY, CQF 806 DALY. Income losses accumulate over time due to long duration paid sick leave. Treatment costs: <2% total human health costs. Using extreme upper and lower bounds; CFS 30% of cases, duration ≥10 yrs, disability weight 0.20, 18.167 Euro/DALY; CFS in 20% of cases, duration ≥5 yrs, disability weight 0.10; 87.602 Euro/DALY | Most long-term benefits implemented control programme reduced disease burden and human health costs. Majority short-term intervention costs in dairy goat sector. Estimated: total loss in public sector: 222 Million Euro; total loss 307 Million Euro. Estimated burden human health 2462 DALY's 2007-2011. CFS most prominent burden | NA | 16/19 checklist items positive ** | | |
| 2013, R. Brooke [25] | Netherlands, yr study NR. Study period Jan 2009-Apr 2010. Duration study NA | Burden of disease study | QF notifications Jan 2009-Dec 2009 (1407 ♂, 906 ♀) vs. influenza notifications Apr 2009-Apr 2010 (1219 ♂, 1508 ♀). Correction for underreporting QF (factor 12.6) and influenza (factor 4.4 to 5.6) | YLD, YLL (2009 Dutch life expectancy), DALYs, BCoDE comparison 2 infectious disease outbreaks | NA | QF: 5797 DALYs, 1771 from acute illness, 4027 from sequelae. PIFS 57% total burden, mainly 45-49 age group. Influenza: 24484 DALYs, 3033 from sequelae. Total no DALYs due to influenza higher than QF, but on per case basis QF more severe. QF is 8.28x worse than influenza regarding composite health measures due to long-term sequelae up to 10 yrs post AI | Intervention prioritization for QF should target immediate interventions for containment and support of long-term sequelae. Long-term sequelae contribute a high burden of disease | NA | NA | | |
| 2013, Y. Arashima [26] | Japan, yr study NR | CR | ♀ 31 yrs, general fatigue, cough, dyspnoea, slightly elevated body temperature, headache, dizziness, poor appetite, copious sweating, night sweating, nausea, vomiting, palpitations. QFS (IgMII 1:16, IgGII 1:128, n-PCR positive) 18 mo post URTI, no result antibiotic treatment. Bronchial asthma 1 mo post URTI, 3 mo steroid inhaler, no improvement. Co-morbidity: moderate/greater depression (SDS 54). Suicide attempt | PS, SDS, n-PCR, IFA | Steroid inhaler 3 mo. Minocycline 200mg/d post diagno-sis QFS | At least 3 mo minocycline: improvement generalized symptoms and bronchial asthma. PS 1. n-PCR negative. IgMII 1:16, IgGII 1:16. FU 9 mo post treatment: bronchial asthma and fatigue disappeared. Depression alleviated | *C.b.* can cause bronchial asthma and should be considered when resistant to standard treatment accompanied by slightly elevated body temperature or general fatigue. Be aware of suicide attempts | Diag, P/T | -/-, ++, ++, ++, ++, NA, +/-, -/- | | |
| 2014, J. van Loen-hout [27] | Netherlands, yr study NR. Study period: 2011-2012, single measurement 12 mo post onset of illness | CC | QF patients (n=309, 53.7% ♂, mean age 49.9 (13.8), current smoker 28.8%, pre-existing health problems 40.6%, hospitalised 36.6%) vs. Legionella patients (n=190, 68.9% ♂, mean age 61.1 (11.5), current smoker 37.4%, pre-existing health problems 59.5%, hospitalised 61.1%), and QF group matched (age, gender) healthy controls (normal lung function, n=121, 55.4% ♂, mean age 51.4)). Assess and compare health status patients 1 yr post QF/Legionella | NCSI, SF-36 | NA | Worse score QF vs. Legionella patients on subdomains fatigue (60.2% vs. 50.0%, i.c.w. 2.5% healthy controls), General Quality of Life (50.0% vs. 42.6%), Role Physical. Adjustment confounders: only Role Physical remained different. In both QF and Legionella: proportion severely affected patients ↑ i.c.w. controls | Certain infectious illnesses are followed by long term impaired health status, including PICF. QF and Legionella patients are affected on ≥1 aspects health status, especially fatigue, General Quality of Life, Role Physical. Impact QF seems higher than from Legionella. Health staff need to be aware of this impact in order to provide adequate care | NA | ★  ★  ★ | ★  ★ | ★ |
| 2014, A. van Dam [28] | Netherlands, 2009-2011, inclusion 1st May-30th September 2009 | CC | 50 QF seropositive LRTI (mean age 48.1, SD14.3) vs. 32 QF seronegative LRTI patients (mean age 57.2, SD14.4); 18-75 yrs. Comparable gender (60% vs. 50% ♂), current smoking (40% vs. 30%), hospitalisation during LRTI (10% vs. 7%), co-morbidity (42% vs. 56%). QF positive: more often pneumonia i.c.w. QF negative. Assess if LRTI due to QF has higher health status impairment i.c.w. other LRTIs 15 mo post AI | NCSI (completion 10-19 mo post LRTI, mean 15 mo). QF positive tested with PCR, IFA or CFT | NA | QF positive LRTI: severely affected General Quality of Life (40%) and fatigue (40%), QF negative LRTI: fatigue (64%) and subjective pulmonary symptoms (35%). 40% QF positive and 56% QF negative severely affected on >1 subdomain. No difference health status scores QF positive and QF negative LRTI patients for all subdomains except subjective pulmonary symptoms | Large group LRTI patients affected >1 aspect of health status 15 mo post LRTI. Little difference in health status QF positive and QF negative LRTI patients. General practitioners ought to be aware of long-term health problems in LRTI patients in general | NA | ★  ★  ★  ★ | ★  ★ | ★  ★ |
| 2015, J. van Loen-hout [29] | Netherlands, study period: 2010-2013, FU at 3, 6, 9, 12, 18, and 24 mo post AQF | CoS | 336 post AQF patients (in 2010-2011, 54.8% ♂, mean age 48.5, SD13.9, co-morbidity 39.7%), comparison NCSI scores matched (age, gender) healthy controls. To assess health status progression of QF patients over 24-mo period, and identify influencing factors | NCSI (3, 12, 18, and 24 mo), SF-36, questionnaires | NA | Significant linear improvement over time in 9/12 health status subdomains. Severely affected: fatigue 73.0% at 3 mo, 60.0% at 12 mo, 37.0% at 24 mo (vs. 2.5% healthy reference group), General Quality of Life 42.2% at 3 mo, 50.2% at 12 mo, 33.7% at 24 mo (vs. 19.8% healthy reference group). For 3 most severely affected subdomains (fatigue, General Quality of Life, Role Physical): females, young adults, pre-existing health problems, at baseline were associated with ↓ long-term health status | Despite linear improvement over time, >1/3 patients had ↓ health status at 24 mo. Results suggest that psychological distress is not an important factor in explaining ↑ fatigue levels | A | ★  ★ | ★  ★ | ★  ★ |
| 2015, J. van Loen-hout[30] | Netherlands, yr study 2011-2013, Single measurement 4 yrs post AQF | CC | 448 notified post AQF (2007-2008, 57.6% ♂, mean age 54.4, SD12.4, co-morbidity 51.1%) vs. 193 symptomatic non-notified post QF (2008-2009, 45.1% ♂, mean age 50.2, SD15.3, co-morbidity 52.6%), vs. healthy controls. To compare long-term health status notified and non-notified QF patients | NCSI | NA | Notified: more ♂, ↑ age vs. non-notified. Equal proportions followed additional treatment for long-lasting health effects of QF, but addition antibiotic treatment slightly ↑ in notified patients. In both groups: fatigue (notified 50.5% vs. non-notified 54.6%) and quality of life (notified 42.3% vs. non-notified 44.4%) most severely affected subdomains. No difference long-term health status notified vs. non-notified, patients scored worse all subdomains i.c.w. healthy controls | Long-term health status is not determined by symptoms during acute QF. Little improvement health status between 1 and 4 yrs post AQF. Implication 2007-2009 Dutch QF outbreak underestimated if only considering notified patients. True burden of disease due to QF outbreak is larger | A | ★  ★ | ★  ★ | ★ |
| 2015, J. van Loen-hout [31] | Netherlands, yr study NR. Study period: 2010-2012, FU 3, 6, 9 and 12 mo post AQF, 12 mo post AI Legionella | CoS, with partly CC | 336 QF, 190 Legionella patients. Assess (progress of) work participation of QF patients up to 12 mo post AQF, identify associated factors, and compare work participation between QF and Legionella patients 12 mo post AI | Questionnaire 3, 6, 9 and 12 mo post AQF, ADIQ at 12 mo both groups | NA | ↓ Proportion QF patients with ↓ work participation, 45% at 3 mo to 19% at 12 mo (vs. 15% Legionella patients at 12 mo). Median proportion reduction hours worked stable over time. ↑ Proportion patients not reporting symptoms up to 12 mo. No symptoms at 12 mo: QF 44% vs. 57% Legionella. Most frequent symptoms at 12 mo QF: fatigue, concentration/memory problems, headache (all 24%), and muscle pain 23%. Legionella: concentration/ memory problems (21%), fatigue, respiratory pro-blems, joint pains (13%). Grieving process: QF ↑ score denial and resistance, ↓ acceptance i.c.w. Legionella. QF; associated factors ↓ work participation: symptoms, ↑ level sorrow, former smoker (i.c.w. never smoked), no alcohol consumption, following treatment for long-term health effects. Median time to full return to work in QF group <3 mo | Almost 1/5 QF patient and 1/6 Legionella patient ↓ work participation at 12 mo. Occupational and insurance physicians need to be aware of long-term impact of QF and Legionella on work participation. Suggestion; undergoing QF leads to grief process similar to progressive disease, underlining the severity of sequelae due to QF | NA | ★  ★  ★  ★ |  | ★  ★ |

****Definition of used study population in articles explained in a different table, including definitions of QFS and/or fatigue is applicable. Main information in this table is on background/descriptive. Some articles also contain relevant information on other domains: Diag= Diagnosis, A= Aetiology, P/T= Prevention/therapy***

***** Quality assessment economic evaluation study was assessed using the ‘Evers checklist’ [32]***

***Abbreviations:*** ADIQ= Acceptance of Disease and Impairments Questionnaire, to assess the different stages of the grieving process due to the infection that patients underwent, AI= Acute infection, AQF= Acute Q-fever, BCoDE= Burden of Communicable Diseases in Europe project, attributes DALYs of an infectious disease to the year the acute infection occurs. This allows for the attribution of long-term sequelae, which may generate a higher number of DALYs, to the causative infection rather than only the initial acute illness, *C.b.=* *Coxiella burnetii,* CBT= Cognitive behavioural therapy, CC= Case-control study, CDC= Centres for Disease Control and Prevention, CDC-SI= German version of the CDC-Symptom Inventory. The inventory asks in detail for 11 symptoms that commonly accompany CFS. These symptoms have to be described with respect to their intensity and frequency related to the last months, CF= Chronic fatigue, CFS= Chronic fatigue syndrome, CFT= Complement fixation test, CIS-R=Revised Clinical Interview Schedule to assess the symptoms of neurotic psychopathology in the week prior to interview. The CIS-R is made up of 14 sections, each covering a particular area of neurotic symptoms. Summed scores from all 14 sections range from 0-57, the overall threshold for clinically significant psychiatric morbidity is 12, CNE= Culture negative endocarditis, CoS= Cohort study, CQF= Chronic Q-fever, CR= Case-report, CS= Cross-sectional, DALY= A composite health measure that represents one lost year of healthy life between the current health status and that of an ideal health situation. Calculated as the sum of YLD for incident cases and the YLL due to premature death, DIOS= Dubbo Infection Outcomes Study**,** cohort study of subjects ≥16 yrs followed from the onset of a confirmed and documented AI due to EBV; *C.b.*; or RRV ≤6 wks post AI until complete recovery, EBV= *Epstein-Barr virus*, ECG= Electrocardiography, F-Sozu K14= To assess social support, a 14-item questionnaire resulting in a total score describing the quality and quantity of a person’s social support, FU= Follow-up, FUO= Fever of unknown origin, GHQ= General health questionnaire, 12-item questionnaire to detect current cases of psychiatric co-morbidity, I.c.w.= In comparison with, ICF= Idiopathic chronic fatigue, IFA= Immunofluorescence assay, IgGI= Anti-phase IgG I titre, IgGII= Anti-phase IgG II titre, IgMI= Anti-phase IgM I titre, IgMII= Anti-phase IgM II titre, LRTI= Lower respiratory tract infection, MFI 20= German version of the Multidimensional Fatigue Inventory, a commonly used 20-item questionnaire indicating different dimensions of fatigue, Mini-DIPS= Diagnostic interview, a short form of the diagnostic interview of psychological disorders, Mo= Month(s), MOS= Medical outcome study 20-item questionnaire, used to define functional impairment in the construction of the CFS definition, NA= Not applicable, NCSI= Nijmegen clinical screening instrument, originally developed to provide a detailed assessment of health status of COPD patients. It combines a number of existing health status questionnaires, NOS= Newcastle–Ottawa Scale: S= selection (maximum of 4 stars), C= comparability (maximum of 2 stars), O= outcome (maximum of 3 stars); ★: star earned; ☆: item not applicable, N/No= Number (of), (n-)PCR= (nested-) Polymerase chain reaction, NR= Not reported, OQ-45= OQ-45, to measure psychological symptoms and general impairment. It is a common symptom inventory used in many psychotherapy studies to reflect total impairment, social as well as interpersonal distress and impairment of social role performance, PHQ-9= a self-administered subset of the PRIMA-MD diagnostic instrument for common mental disorders to assess symptoms severity of depression, PICF= Post-infectious chronic fatigue, PIF(S)= Post-infective fatigue (syndrome), P.o.= Oral, PO= Personal opinion, POB= Personal observation, PQCFS= Post-Q-fever chronic fatigue syndrome, PQDS= Post-Q-fever debility syndrome, PQFS= Post-(acute)Q-fever (fatigue) syndrome, Pros.= Prospective, PS= Performance status score (range 0-9), which reflects the grade of fatigue/malaise to assess the severity of CFS, QA-CR= Quality assessment; for CR no quality checklists are available. Therefore, the following eight criteria for quality assessment were determined; addressing an appropriate and clearly focused question, representative population, description of the survey method or data collection, outcome measures defined, outcome measures described, response rate reported and results valid and applicable to the patient group targeted. The articles scores on these items: -/-, -, +/-, +, or ++, based on the Coordination of Cancer Clinical Practice Guidelines in Europe criteria, QF= Q-fever, QF(F)S= Q-fever fatigue syndrome, Ref= Reference, RRV= *Ross River virus*, SD= Standard deviation, SDS= Self-rating depression scale, consisting of 20 questions, score per question: 1-4 points, SDQ= Somatic Discomfort Questionnaire, a checklist of 25 somatic symptoms, as somatic symptoms are important minor symptoms in the construction of CFS definition, SF-12= The Short Form (12) Health Survey, SF-36= The Short Form (36) Health Survey, a patient-reported survey of patient health to assess quality of life of patients, functional impairment and reduced health related quality of life, SOMA= Empirically derived subscale of the SPHERE, used to record PIFS or illness duration. This reliably predicts disability and reflects patients’ and doctors’ reports of reasons for presentation to primary care. Scores ≥3 represents a clinically-significant fatigue state. Provisional PIFS: SOMA scores ≥3 at all time points up ≤3 months. Confirmed PIFS: symptoms persisted >6 months, and alternative explanations for ongoing illness was excluded, SOMS= Screening for Somatoform Disorders, a 53-item questionnaire assessing symptoms common for somatoform and somatisation disorder leading to the calculation of different indices, SPHERE= Somatic and Psychological Health Report, to assess a wide range of physical and psychological symptoms, including severity and duration of symptoms, *T. gondii= Toxoplasma gondii,* UK= United Kingdom, URTI= Upper respiratory tract infection, VAS= Visual analogue score, 10cm scale to quantify symptom severity, Wks= Weeks, WI= Whiteley Index, to measure the patients’ tendency for hypochondriacal worries and beliefs, YLD= Number of years lost due to disability: number of incident cases x average duration of the disease x weight factor that reflects the severity of the disease on a scale from 0 (perfect health) to 1 (dead), YLL= Years of Life Lost due to premature death; number of deaths caused by the disease x standard life expectancy at the age at which death occurs, Yr(s)= Year(s)

**References**

1. Powell O. "Q" fever: clinical features in 72 cases. Aust Ann Med. 1960;9:214-23. PubMed PMID: 13737616.

2. Reilly S, Northwood JL, Caul EO. Q fever in Plymouth, 1972-88. A review with particular reference to neurological manifestations. Epidemiol Infect. 1990;105(2):391-408. Epub 1990/10/01. PubMed PMID: 2209742; PubMed Central PMCID: PMCPmc2271878.

3. Harvey-Sutton PL. Post-Q fever syndrome. Med J Aust. 1995;162(3):168. Epub 1995/02/06. PubMed PMID: 7854246.

4. Marmion BP, Shannon M, Maddocks I, Storm P, Penttila I. Protracted debility and fatigue after acute Q fever. Lancet. 1996;347(9006):977-8. Epub 1996/04/06. PubMed PMID: 8598796.

5. Ayres JG, Smith EG, Flint N. Protracted fatigue and debility after acute Q fever. Lancet. 1996;347(9006):978-9. Epub 1996/04/06. PubMed PMID: 8598797.

6. Ayres JG, Flint N, Smith EG, Tunnicliffe WS, Fletcher TJ, Hammond K, et al. Post-infection fatigue syndrome following Q fever. QJM. 1998;91(2):105-23. Epub 1998/05/14. PubMed PMID: 9578893.

7. Kato K, Arashima Y, Asai S, Furuya Y, Yoshida Y, Murakami M, et al. Detection of Coxiella burnetii specific DNA in blood samples from Japanese patients with chronic nonspecific symptoms by nested polymerase chain reaction. FEMS Immunol Med Microbiol. 1998;21(2):139-44. Epub 1998/07/31. PubMed PMID: 9685003.

8. Wildman MJ, Smith EG, Groves J, Beattie JM, Caul EO, Ayres JG. Chronic fatigue following infection by Coxiella burnetii (Q fever): ten-year follow-up of the 1989 UK outbreak cohort. QJM. 2002;95(8):527-38. Epub 2002/07/30. PubMed PMID: 12145392.

9. Marmion BP, Harris RJ, Storm PA, Semendric L. Q fever: still a mysterious disease. QJM. 2002;95(12):832-3. Epub 2002/11/28. PubMed PMID: 12454328.

10. Raoult D. Q fever: still a mysterious disease. QJM. 2002;95(8):491-2. Epub 2002/07/30. PubMed PMID: 12145387.

11. Harris RJ, Storm PA, Lloyd A, Arens M, Marmion BP. Long-term persistence of Coxiella burnetii in the host after primary Q fever. Epidemiol Infect. 2000;124(3):543-9. Epub 2000/09/12. PubMed PMID: 10982079; PubMed Central PMCID: PMCPmc2810941.

12. Wildman MJ, Ayres JG. Q fever: still a mysterious disease. QJM. 2002;95(12):833-4. Epub 2003/01/15. PubMed PMID: 12524714.

13. Hatchette TF, Hayes M, Merry H, Schlech WF, Marrie TJ. The effect of C. burnetii infection on the quality of life of patients following an outbreak of Q fever. Epidemiol Infect. 2003;130(3):491-5. Epub 2003/06/27. PubMed PMID: 12825734; PubMed Central PMCID: PMCPmc2869986.

14. Thomas HV, Thomas DR, Salmon RL, Lewis G, Smith AP. Toxoplasma and coxiella infection and psychiatric morbidity: a retrospective cohort analysis. BMC Psychiatry. 2004;4:32. Epub 2004/10/20. doi: 10.1186/1471-244x-4-32. PubMed PMID: 15491496; PubMed Central PMCID: PMCPmc526777.

15. Hickie I, Davenport T, Wakefield D, Vollmer-Conna U, Cameron B, Vernon SD, et al. Post-infective and chronic fatigue syndromes precipitated by viral and non-viral pathogens: prospective cohort study. BMJ (Clin Res ed). 2006;333(7568):575. Epub 2006/09/05. doi: 10.1136/bmj.38933.585764.AE. PubMed PMID: 16950834; PubMed Central PMCID: PMCPmc1569956.

16. Limonard GJ, Peters JB, Nabuurs-Franssen MH, Weers-Pothoff G, Besselink R, Groot CA, et al. Detailed analysis of health status of Q fever patients 1 year after the first Dutch outbreak: a case-control study. QJM. 2010;103(12):953-8. Epub 2010/08/31. doi: 10.1093/qjmed/hcq144. PubMed PMID: 20802011.

17. Limonard GJ, Nabuurs-Franssen MH, Weers-Pothoff G, Wijkmans C, Besselink R, Horrevorts AM, et al. One-year follow-up of patients of the ongoing Dutch Q fever outbreak: clinical, serological and echocardiographic findings. Infection. 2010;38(6):471-7. Epub 2010/09/22. doi: 10.1007/s15010-010-0052-x. PubMed PMID: 20857313; PubMed Central PMCID: PMCPmc3003145.

18. Morroy G, Peters JB, van Nieuwenhof M, Bor HH, Hautvast JL, van der Hoek W, et al. The health status of Q-fever patients after long-term follow-up. BMC Infect Dis. 2011;11:97. Epub 2011/04/20. doi: 10.1186/1471-2334-11-97. PubMed PMID: 21501483; PubMed Central PMCID: PMCPmc3110112.

19. van Woerden HC, Healy B, Llewelyn MB, Matthews IP. A nested case control study demonstrating increased chronic fatigue six years after a Q fever outbreak. Microbiol Res. 2011;2(e19):69-72. doi: 10.4081/mr.2011.e19.

20. Strauss B, Loschau M, Seidel T, Stallmach A, Thomas A. Are fatigue symptoms and chronic fatigue syndrome following Q fever infection related to psychosocial variables? J Psychosom Res. 2012;72(4):300-4. Epub 2012/03/13. doi: 10.1016/j.jpsychores.2012.01.010. PubMed PMID: 22405225.

21. Morroy G, Bor HH, Polder J, Hautvast JL, van der Hoek W, Schneeberger PM, et al. Self-reported sick leave and long-term health symptoms of Q-fever patients. Eur J Public Health. 2012;22(6):814-9. Epub 2012/02/09. doi: 10.1093/eurpub/cks003. PubMed PMID: 22315459.

22. Arashima Y, Yakubo S, Nagaoka H, Komiya T, Murakami M, Nakayama T, et al. A patient in whom treatment for coxiella burnetii infection ameliorated a depressive state and thoughts of impending death. International Medical Journal. 2012;19(1):65-6. PubMed PMID: 2012223501.

23. Yakubo S, Ueda Y, Tanekura N, Arashima Y, Nakayama T, Komiya T, et al. The first case of a patient suffering from Coxiella burnetii infection attempting suicide arising from a state of depression. International Medical Journal. 2012;19(4):312-3. PubMed PMID: 2012700928.

24. van Asseldonk MA, Prins J, Bergevoet RH. Economic assessment of Q fever in the Netherlands. Prev Vet Med. 2013;112(1-2):27-34. Epub 2013/07/23. doi: 10.1016/j.prevetmed.2013.06.002. PubMed PMID: 23866818.

25. Brooke RJ, van Lier A, Donker GA, W VDH, Kretzschmar ME. Comparing the impact of two concurrent infectious disease outbreaks on The Netherlands population, 2009, using disability-adjusted life years. Epidemiol Infect. 2014:1-10. Epub 2014/01/31. doi: 10.1017/s0950268813003531. PubMed PMID: 24476696.

26. Arashima Y, Yakubo S, Ueda Y, Munemura T, Komiya T, Isa H, et al. A first case of asthma thought to be caused by coxiella burnetti infection. International Medical Journal. 2013;20(6):699-700. PubMed PMID: 2014054225.

27. van Loenhout JA, van Tiel HH, van den Heuvel J, Vercoulen JH, Bor H, van der Velden K, et al. Serious long-term health consequences of Q-fever and Legionnaires' disease. J Infect. 2014. Epub 2014/01/29. doi: 10.1016/j.jinf.2014.01.004. PubMed PMID: 24468188.

28. van Dam S, van Loenhout JA, Peters JB, Rietveld A, Paget WJ, Akkermans RP, et al. A cross-sectional study to assess the long-term health status of patients with lower respiratory tract infections, including Q fever. Epidemiol Infect. 2014:1-7. Epub 2014/03/15. doi: 10.1017/s0950268814000417. PubMed PMID: 24625631.

29. van Loenhout JA, Hautvast JL, Vercoulen JH, Akkermans RP, Wijkmans CJ, van der Velden K, et al. Q-fever patients suffer from impaired health status long after the acute phase of the illness: results from a 24-month cohort study. J Infect. 2015;70(3):237-46. doi: 10.1016/j.jinf.2014.10.010. PubMed PMID: 25452036.

30. van Loenhout JA, Wielders CC, Morroy G, Cox MJ, van der Hoek W, Hautvast JL, et al. Severely impaired health status of non-notified Q fever patients leads to an underestimation of the true burden of disease. Epidemiol Infect. 2015:1-8. Epub 2015/01/15. doi: 10.1017/s0950268814003689. PubMed PMID: 25582890.

31. van Loenhout JA, Hautvast JL, Akkermans RP, Donders NC, Vercoulen JH, Paget WJ, et al. Work participation in Q-fever patients and patients with Legionnaires' disease: A 12-month cohort study. Scand J Public Health. 2015;43(3):294-301. doi: 10.1177/1403494815571030. PubMed PMID: 25724468.

32. Evers S, Goossens M, de Vet H, van Tulder M, Ament A. Criteria list for assessment of methodological quality of economic evaluations: Consensus on Health Economic Criteria. Int J Technol Assess Health Care. 2005;21(2):240-5. PubMed PMID: 15921065.
