# Supplementary material for: Fatigue following Acute Q-Fever: A Systematic Literature Review
Source: PLoS One. 2016 May 25;11(5):e0155884. doi: 10.1371/journal.pone.0155884 (PMC4880326; doi:10.1371/journal.pone.0155884)
Supplement: S4 Table — (DOCX) [file pone.0155884.s005.docx]

**S4 Table. Domain aetiology**

| **Ref** | **Country, yr study, period and duration** | **Study type** | **Patients, controls, characteristics, co-morbidity*** | **Tool** | **Inter-**  **ven-**  **tion** | **Outcome** | **Conclusions/recommendations** | **Other do-main** | **QA (NOS)** | | |
| --- | --- | --- | --- | --- | --- | --- | --- | --- | --- | --- | --- |
| 1998, B. Bennet [1] | Australia, yr study NR (sub study DIOS). Study period NR | Pros. CoS | 17 EBV, 8 QF, 5 RRV (82% ♂, mean age 29 (15-77)). Explore longitudinal relationships between physical and psychological symptoms and immunological factors during peak illness (symptoms <4 wks before presentation) and recovery phase (2 and 4 wks after baseline) of AI (EBV, *C.b.*, and RRV) | Baseline: interview, POMS, GHQ, SOFA, CIDI, DTH skin response. At 2 wks: interview, POMS, GHQ, SOFA. At 4 wks: interview, POMS, GHQ, SOFA, DTH test | NA | Baseline: fatigue and malaise most common symptoms. Depressive and anxiety symptoms not prominent. 46% cases no DTH skin response, indicative of impaired cell-mediated immunity. Over 4 wk period, improvement somatic and psychological symptoms, but 63% remained fatigue. Most symptoms improved; somatic changes notable in fatigue and malaise, rather than psychological (anxiety and depression). Psychological changes due to changes in perception fatigue and vigour. ↓ reported fatigue correlated with ↑ DTH skin response (indicating relation between fatigue and cell-mediated immunity) and GHQ scores | Fatigue commonly remains a prominent complaint at 4 wks. Resolution of fatigue is associated with improvement in cell-mediated immunity, supporting an immunological basis for PIF | NA | ★  ★  ★  ★ |  | ★  ★ |
| 1998, I. Penttila [2] | Australia, yr study NR. Single measure-ment study | CC | 18 QFS patients (mean age 39 (33-45), symptom score >100, mean 145 (133-157)). 27 controls; 6 resolving QFS (symptom score <100, mean age 39 (31-48)), 5 past AQF (>6 mo) without QFS (symptom score 15 (1-35), mean age 39 (25-54)), 8 QF skin test - vaccinated (mean age 44 (31-56), symptom score 7 (1-13)), 8 QF - healthy controls (mean age 32 (23-40), symptom score 5 (1-9)). Mean age groups equal. Asses cytokine release patterns PBMC stimulated with QF (phase I and II), measles antigens and PHA, in 72-h culture | Interview; clinical history, presence, frequency, and intensity of 16 minor and major QFS symptoms scored on linear numerical scale 1-20. Analysis of: IL-1, IL-2, IL-4, IL-5, IL-6, IL-10, IFNy, TNFa, TNFb and TGFB | NA | Aberrant cytokine release patterns of PBMC QFS patients stimulated with QF antigens; ↑ IL-6 release (mean 502 pg/ml, i.c.w. 47-53 pg/ml in other groups, p=0.018). Mean PBMC response of QFS to PHA ↑ than controls, not significant. 72% QFS patients: IL-6 release values >100 pg/ml; 66pg/ml max value for 95% CI of control groups. QFS ↓ IL-2 responders with QF antigens (p=0.014), but equal IL-2 release post PHA stimulation. QFS: ↑ IFNy responders (p=0.0008); no difference median [IFNy] released (p=0.14). IL-1 ↑ on PHA stimulation PBMC from QFS than controls (p=0.03), no difference with QF antigens. >IL-5 responders among QFS with QF antigens. Correlation IL-6 in conditioned medium, total symptom score, and scores other key symptoms. In QFS: persistent IL-6 upregulation, no time-correlation with AQF | Hypothesis: cytokine deregulation due to chronic immune stimulation and modulation by persistent *C.b.*/antigens. Aberrant IL-6 response not claimed to explain QFS pathogenesis. IL-6 might contribute to QFS symptomatology. QFS development appears to require cellular immune response against *C.b.* antigens. A speculative unifying concept is that QFS and QIE represent different poles of dysfunctional cell-mediated immunity response to *C.b*. QFS patients have positive LMR to QF antigens, greatly ↑ IL-6 release patterns from PBMC, IFNy upregulated, but IL-2 downregulated. Fatigue can be intermittent (relapsing) or continuous in QFS | Diag, B/D | ★  ★  ★  ★ | ★ | ★  ★  ★ |
| 2000, R. Harris [3] | Australia, yr study NR. Study period NR. Mean period sampling 37 mo post AI (2 after 9 mo, remainder after ≥12) | CC | QFS (n=29); 18 from [2], 11 additional with QFS post AQF. PBMC (n=29), liver biopsy (n=14), BMA (n=20). Controls from [2]. PBMC: patients no QFS post AQF (n=5); post-vaccination (n=7), *C.b.* seronegative healthy controls without CFS (n=6). BMA (n=6) of patients with diseases other than QF. Positive PCR controls; QIE or recrudescent infection in pregnancy (n=10) | PCR (target IS1111a), several primer sets in conventional PCR and TaqMan PCR system | NA | *C.b.* detection in QFS: PBMC 5/29, liver biopsy 2/14, BMA 13/20. In PBMC: no QFS 0/5, vaccinated 0/7, seronegative 0/6. In BMA: other diseases 0/6. PCR positive in QIE/placentitis 10/10 | *C.b.* DNA in bone marrow 0.75-5 yrs post AQF infection unveils new QF pathology state. *C.b.* live/dead/other bio-entities not defined. Pattern suggestive paucibaccillary infection presumably under immune control, but not eliminated. Supports previous reports relationship QFS, cytokine dysregulation and immunomodulation from *C.b.* persistence. Bone marrow could be focus cryptic infection which might seed other sides. Before drawing conclusions on QFS, investigate bone marrow in more patients with/without QFS/other sequelae | Diag | ★  ★  ★  ★ |  | ★  ★ |
| 2002, J. Ayres [4] | UK, 1999. Study period 10 yrs post AQF | Nested CC | N=85 *C.b.*-exposed (85.6% ♂, mean age 54.7, SD12.0, co-morbidity 29.4%) vs. n=75 matched (sex & smoking) QF seronegative controls (86.7% ♂, mean age 55.3, SD11.4, co-morbidity 29.3%). Determine if persistent fatigue post AQF represents sub-clinical cardiomyopathy | Questionnaires, 12-lead ECG, echocardiography, spirometry, shuttle walk distance, MUGA scan (only in subset) | NA | 68.2% *C.b.* cases fatigue any duration, 42.4% fatigue excluding co-morbidity. 20% CDC-defined CFS vs. 5.3% controls, 8.2% excluding co-morbidity vs. 0% controls. Normal ECG's 76.5% cases, 69.3% controls, no differences. Echocardiography: controls ↓ fractional shortening. Fatigued vs. non-fatigued QF cases: comparable echocardiography, ECG, shuttle walk distances, pack years smoking. Normal MUGA scan 6 *C.b.* cases (CDC-defined CFS without co-morbidity) | Findings do not support the existence of a sub-clinical cardiomyopathy in patients with fatigue after AQF, therefore not explaining breathlessness and fatigue. Chronic heart disease following AQF is rare and limited to IE | B/D | ★  ★  ★ | ★  ★ | ★  ★  ★ |
| 2002, D. Raoult [5] | France, 2002. Duration study NA | PO | No patients/controls. Characteristics and co-morbidity: NR | NA | NA | 6 mo post AQF 5-10% residual asthenia, very few >1 yr. Subjective symptoms difficult to quantify. CF: difficult to define, with different prevalence. Unknown if CF psychological in origin/directly caused by bacterium. Might reflect observational bias, *C.b.* strain or cultural differences, or genetic susceptibility | Amplicon production PCR in peripheral blood CF patients needs confirmation. New tools might allow to examine aetiology incompletely understood diseases caused by intracellular bacteria | B/D | NA | | |
| 2003, K. Helbig [6] | Australia, yr study NR. Single measurement study | CC | 23 active/recovered QFS, 42 controls Red Cross blood donors, all Caucasians. To compare variability in phenotype distribution among range of cytokine and accessory immune response genes in PQFS and controls | Genotyping within NRAMP1 gene, HLA typing for HLA-DR and HLA-B; 25 polymorphic variants 14 genes analysed | NA | No significant variation frequency individual SNP patients and controls, but more variants differing from wild type in patients i.c.w. controls, p=0.025. Differences allelic frequencies HLA-DR, significant ↑ frequency HLA-DR11 in QFS, but not HLA-B. Phenotype frequencies SNP in genes not significantly different from controls. Variation allele distribution QFS and controls INFy di-nucleotide repeat. IFNy genes; ↑ prevalence homozygous state IFNy allele 2 in intron 1 in QFS | Possible genetic role expression overt chronic manifestations, e.g. individual variation *C.b.* immune response. Given complexity of genetic control of immune system, a simple 1-to-1 relation between QFS expression/other chronic complication QF and a particular polymorphic variation in a cytokine or immune control gene is unlikely. Effects are more likely multigenic | Diag | ★  ★  ★ | ★ | ★ |
| 2003, K. Ikuta [7] | Japan, yr study NR. Single measurement study | CC | 44 CFS (H1: 22 CFS, 14 ♂, 23-61 yrs; H2: 22 CFS, 17 ♂, 20-46 yrs), 38 healthy controls (20 ♂, 20-59 yrs). To investigate association viral infections with CFS and 2-5AS activity in PBMC in Japan in 2 hospitals (H1, H2) different areas | *C.b.,* IFA IgGII positive titre ≥1:64 | NA | 2-5AS activity: 19 (mean 2.23) in H1, 7 (mean 0.91) in H2, 4 in controls (mean 0.74). Differences H1 and H2, and H1 and controls (p<0.01). No difference H2 and controls. IFNa similar in few CFS patients and controls. No relationship 2-5AS and IFNa positivity. EBV anti-EA-IgG antibodies in 9% and 32%in H1 and H2. IgG *C.b.* positive 6/22 H1, 0/22 H2, 1/9 controls. No difference *C.b.* positive H1 and controls/patients H2 and controls. No correlation 2-5AS activity and *C.b.* titres (p>0.05) | 2-5AS activity ↑ PBMC CFS patients. CFS may be associated EBV/*C.b*. ↑ 2-5AS suggests immunological dysfunctions with virus infections in CFS. No relation titres *C.b*. and 2-5AS activities. 2-5AS activity changed from positive to negative in 1 CFS patient when *C.b.* antibodies disap-peared, suggests *C.b.* association 2-5AS activity some CFS patients. Imply 2-5AS in some CFS patients activated by other mechanisms, in addition to EBV and *C.b.* | NA | ★  ★ |  | ★  ★ |
| 2005, B. Marmion [8] | Australia and UK, 2001, study period NR | CC (case follow-up study) | *C.b.* positive UK cases (n=92) 12 yr post AQF (Birmingham 1989, n=92 blood samples, n=91 PBMC, n=35 BMA), Australian cases (n=29) 9 mo-5 yrs post AQF (n=29 blood samples and PBMC, n=20 BMA, n=14 liver biopsy) with CFS (CDC-criteria). To compare prevalence infection markers between cohorts | I. *C.b.* PCR (directed against several targets in the genome) DNA detection PBMC and bone mar-row, II. CFT, IFA Phase I & II, III. isolation *C.b.* cell cultures of mice - PCR positive | NA | Both groups remained seropositive irrespective clinical state. *C.b.* genomic DNA detected by PCR in 65% of BMA from Australian vs. 88% Birmingham patients. No *C.b.* isolated from PCR positive samples | Results indicate more complex interaction between host-regulated, persistent carriage of *C.b.* and disease. An additional variable factor of host regulation of cellular immune response must determine levels of persistence and symptomatic outcomes. Hypothesis: in QF without sequelae, process largely confined to bone marrow. In QFS, modulation by the patient’s immunogenetic background causes ↑ levels of *C.b.* genomes in bone marrow and ↑ shedding into peripheral blood | Diag | ★  ★★  ✰ |  | ★★ |
| 2005, K. Helbig [9] | Australia and UK, yr NR, study duration NR | CC (gene-tic asso-ciation) | 31 QFS patients vs. uncomplicated recovery up to 12 yrs post AQF (n=22) vs. QIE (n=22, mean age 57, range 29-78, time lag infection-IE 8.8 yrs, SD12, range 2-40) i.c.w. standard control panels general population. To compare frequencies of allelic polymorphisms in immune response genes in different QF patient groups | Whole blood, DNA extraction, HLA typing, microsatellite typing, SNP analysis | NA | Significant differences between 3 groups. QFS patients differed from QIE, the uncomplicated and controls in frequency of HLA-DRB1*11 and 2/2 genotype of IFNy intron 1 microsatellite. Carriage HLA DRB1*11 allele associated with ↓ IFNy and IL-2 responses from PBMC. QIE showed differences in IL-10 promoter microsatellites R and G, and ↑ frequency TNFa receptor II 196R polymorphism. QF patients with uncomplicated recovery, differed from those with QFS/QIE, but similar in allelic frequencies to control panels | Conclusions *C.b.*, parvovirus B19 infection and CFS studies suggest that ‘idiopathic’ CFS patients from the wider population, away from outbreaks/occupationally exposed groups, are unlikely to have laboratory evidence of infection with the same infective agent. A common immuno-genetically determined failure of cytokine homeostasis to infective agents with the capacity to persist long in hosts is more likely | Diag | ★  ★★  ★ | ✰✰ | ★★ |
| 2007, U. Vollmer-Conna [10] | Australia, 1999 (sub study DIOS); 12 mo collection period. Appraisal 1, 2, 3, 6, 12 mo post AI | Pros. CoS | 22 PIFS patients (11 EBV, 6 RRV, 5 *C.b.*) vs. 42 aged-matched controls who recovered <6 wks of EBV (n=17), RRV (n=14), and QF (n=11). Analysis influence PIFS status on symptom severity and cytokine production i.c.w. controls | SPHERE, BDQ. SOMA score ≥3 to record PIFS | NA | No group differences cytokine levels. Severity symptoms ↓ in time. ↑ Age associated with ↑ musculoskeletal pain and neurocognitive disturbance. PIFS stereotyped post different triggers, with equal acute-phase cytokine production. Psychological/ microbial factors not predictive PIFS. PIFS: ↑ mean no. bed-days acute phase, and more days “out of role” | Ongoing production IL-1b, IL-2, IL-4, IL-6, IL-10, IL-12, TNFa and INFy have no role in PIFS. Evidence against hypothesis associating prolonged fatigue with altered cytokine levels. AI triggers, not drives symptoms. PIFS can persist wks to mo | B/D | ★★★★ | ★★ | ★★ |
| 2009, B. Marmion [11] | Australia and UK, yr study NR | Laboratory case study | 10 Birmingham (1989) *C.b.* PCR positive and 1 IE. To retest PCR positive samples with more sensitive methods for viable *C.b.* and *C.b.* cell components antigen and specific LPS ≥12 yrs post AQF, and re-interpret previous results. Review literature for a concept of immunomodulatory complex generated by current studies | 3 SCID mice; spleen and liver examination by PCR (targets COM1 and IS1111a sequences), IFA | Inoculation patient sam-ples in SCID mice for 60 d | All patients’ specimens including heart valve with endocarditis were infection negative in SCID mice. Mice spleens and livers PCR negative. Spleen sections of all specimens showed Coxiella antigen LPS complex by IFA | Long-term persistence non-infective, biodegradable immunomodulatory complex traces genomic DNA. Immuno-modulatory complex survival >12 yrs, in 1 patient 70 yrs, implies repeated passage macrophages ↓ regulation biodegrading function. Systemic symptoms QFS may reflect wide distribution parasitized mononuclear phagocytes. QFS follows clinical overt infection, rarely subclinical infection | NA | NA | | |
| 2009, L. Zhang [12] | United Kingdom, yr study and period NR. Single measurement study | CC | 117 patients idiopathic CFS/ME; 6 Q-CFS/ME. Controls: endogenous depression (n=14), blood donors (n=29). Attempt to reproduce genomic subtypes CFS/ME (with distinct: SF-36, clinical phenotypes, severity and geographical distribution), determine specificity signature CFS/ME, and test associations CFS/ME subtype and infection by determining expression levels 88 human genes | Chalder Fatigue Scale, SF-36, Somatic and Psychological Health Report, PSQ, McGill Pain Questionnaire, PAXgene blood RNA kit, micro-spectrophoto-metry, qPCR | NA | In CFS/ME differential expression confirmed for all 88 genes. 8 genomic CFS/ME subtypes with marked differences global functioning, clinical symptoms, severity levels and geographical distribution. Q-CFS/ME similar patterns gene expression in peripheral blood to idiopathic CFS/ME, and markedly different from normal group. 5/6 Q-CFS/ME patients clustered in subtype A, but no subtype-specific relationships found with *C.b.* antibodies. Evidence subtype-specific relationships EBV and enterovirus. Gene expression in endogenous depression similar to normal controls, except ↑ regulation 5 genes (APP, CREBBP, GNAS, PDCD2, and PDCD6). Q-CFS/ME patients ↑ McGill Pain Questionnaire scores i.c.w. other groups. SF-36 ↑, Mental and physical fatigue, and SPHERE scores ↓ i.c.w. all groups, except normal blood | Q-CFS/ME had similar patterns gene expression as idiopathic CFS/ME | Diag, B/D | ★  ★  ★ |  | ★  ★ |
| 2010, Y. Kadota [13] | Australia, 1999 (sub study DIOS); single measurement | Pros. CC | 23 PIFS patients (9 RRV, 7 EBV, 4 QF, 3 viral infection unknown origin) vs. 25 matched (age, sex, BMI, activity levels) healthy controls. Evaluation association of PIFS with bidirectional autonomic signalling disturbance | Pulse oximeter, pain test algometer, Stroop task, SPHERE, SOMA K10, BDQ, DS14 | NA | PIFS patients: ↑ symptoms in general, fatigue related, or psychological distress, more days not fulfilling normal roles past mo, ↑ experience negative emotions, ↑ reporting functional impairment daily activities, ↑ resting heartbeat, ↑ sensitivity to physiological signals. Relation between heartbeat discrimination accuracy and pressure pain sensitivity. Different heart rate pattern in response to ongoing mental stressors | PIFS: ↑ interoceptive sensitivity (with strong symptoms correlation), distinct pattern cardiac response; evidence physiological hyper-vigilance and response inflexibility. ↑ Resting heart rate with ↓ heart rate variability: ↓ parasym-pathic drive. Autonomic dysfunction involves both disturbance processing incoming homeostatic information, and altered reactivity to stressors | B/D | ★★★ | ★★ | ★  ★  ✰ |
| 2010, O. Sukoche-va [14] | Australia, yr study NR, duration NR | CC (laboratory case study) | No patients/controls. Samples post AQF patients (Birmingham, 1989), 3 groups; recGr3: asymptomatic recovery post AQF. QFSGr5: QFS, no co-morbidity. QFSGr6: QFS fatigue associated co-morbidity. 12 yrs post outbreak, groups sampled *C.b.* antibody, blood leucocytes, PCR on BMA. PCR positive samples (bone marrow, PBMC, or aortic valve specimens) 10 patients from subsets inoculated intraperitoneal NOD/SCID mice. Control animals received blood PCR negative, seronegative controls. To isolate living *C.b.* to ascertain pathological effects, retest and determine nature residual *C.b.* cell components | Cell culture assay, PCR (target COM1 and IS1111a), CBA, skin granuloma test in guinea pigs, immunoche-mistry,histoche-mistry, image acquisition | Inoculation patient sam-ples in NOD/SCID mice, FU for infec-tion evidence and presence DNA and specific anti-gens in spleen and liver macrophages | Culture samples 10 QF patients NOD/SCID mice, 12 yrs post AQF no viable *C.b*. No AI induced. Complexes material *C.b.* antigens found in mouse spleens, significantly higher amounts in samples QFS, also in bone marrow and liver in all cases. Immunomodu-latory complex stimulate cytokine release in mice and THP-1 macrophages, and to provoke inflammatory reaction on intradermal injection into skin of QF hyperimmunized guinea pigs (with Qvax). QFSGr5 and 6: weight ↓ 1^st^ week post inoculation, later recovered and steady weight gain consistent with absence infection. All mouse spleen specimens PCR negative (1:100 dilutions). Despite absence active infection, changes: moderate spleen enlargement QFSgr5 and 6 i.c.w. controls (p<0.05), no massive splenomegaly by live *C.b*. Sections mouse spleens with variable amounts aggregates stained to detect specific antigen, also in NOD/SCID mouse bone marrow and liver inoculated with QFS specimens. *C.b.* antigens no correlation low levels *C.b.*, suggests complexes to represent incompletely degraded cell material. *C.b.* antigens localized in spleen phagocytes, and *C.b.* immunomodulatory complex in lysosomes mouse splenocytes. L-6/IL-10 ratio and ↑ level IL-10 might signal important role in facilitating survival non-degraded bacterial material | In QFS viable, infective *C.b.* are rarely, if ever, isolated from PBMC or bone marrow, but complex of antigen and Phase 1 LPS (immunomodulatory com-plex) is regularly present. This non-infec-tive complex of *C.b.* antigens survives in host and provokes aberrant humoral and cell-mediated immunity responses – a possi-ble pathogenic link between initial infec-tion and PQFFS. Different responses between endocarditis, asymptomatic/ recovered and QFS patients considered due to immunogenetic differences in handling immunomodulatory complex and cytokine responses. Hypothetical pathogenetic sequence QFS; overt clinical QF and immunogenetic polymorphism --> defective antigen clearance (immune-modulatory complex persistence) --> persistent cell-mediated immunity and cytokine dysregulation --> cytokine-mediated somatic gene modulation --> QFS | NA | NA | | |
| 2011, S. Galbraith [15] | Australia, yr study NR (sub study DIOS). Study period: baseline measure (T1 0<6 wks), T2 6<12 wks, T3 3<9 mo or >9 mo, T4 >12 mo, FU after 2 and 4 wks | Longitudinal, nested CC | Caucasians with PIFS (n=18; EBV, RRV, *C.b.*) (mean age: 40, SD18 years). Matched (age, sex & infection type) controls (n=18) who recovered promptly (mean age: 39, SD16). 11 ♂ per group. 127 samples analysed, 3-4 time points/subject. In longitudinally collected samples peripheral blood transcriptomes studied for gene expression patterns in PIFS patients and controls. Differential expression sought between early illness and late recovery (within-subject comparison), PIFS cases and recovered controls (between subjects comparison), and genes correlated with end phenotypes derived by principal components analysis (between-cohorts) | Microarray and confirmatory qPCR. SPHERE, SOMA | NA | 23 genes with modest differential expression (0.6-2.3-fold change) in within-subject comparisons of early, symptomatic time points with late, recovered time points. Modest differences 63 genes, in CS comparison cases-controls 6 mo post AI in regression model. 223 genes correlated with individual symptom domains. qPCR confirmed 33/45 genes, none consistent across cohorts. Within subject comparison: 12 subjects (5 with QF) T1 SOMA scores ≥3, T4 SOMA scores <3. No genes with adjusted significance <0.05. Relative lack variance gene expression levels over ≥12 mo. Between subject comparisons: 17 cases (6 QF), 11 controls (2 QF). No genes with adjusted significance <0.05. QF subjects predominantly ♂ and older. 13 genes adjusted significance <0.05, 1 (CYBA) associated with fatigue in 2 of 3 infective cohorts (EBV, QF). Analysis identified illness severity, fatigue and neurocognitive disturbance, correlated for EBV and QF cohorts. Correlation test: 96 genes unadjusted significant at 5% for EBV and QF for severity, 93 for fatigue symptom domain, 106 for neurocognitive disturbance. Repeated correlation analysis: no genes correlated for EBV and QF in association with severity, fatigue, neurocognitive disturbance | Several infections trigger PIFS, which share key illness characteristics with each other and CFS. Previous CS CC studies of CFS suggested unique gene expression signature in peripheral blood samples. Although illness characteristics of PIFS patients have more similarities than differences, no reliable peripheral blood gene expression correlate is evident. No genes consistently associated with illness. CFS incidence closely comparable between EBV, RRV, *C.b.* Lack of coherent set of gene expression correlates across cohorts argues against validity of previously proposed signatures for PIFS or CFS. PIFS likely to be truly post-infective, un-associated with ongoing active replication of triggering agent | NA | ★  ★  ★  ★ | ★  ★ | ★  ★ |
| 2012, B. Piraino [16] | Australia, yr study NR (sub study DIOS). Study period NR. Baseline, FU 2-3wks, 4-6wks, 3-mo interval until 12 mo post AI | CoS | Caucasians (mean age 34.2, 49% ♀), <6 wks post AI (n=296), EBV, RRV, QF. Princi-pal components analysis acute phase, self-report symptom data to empirically derived indices fatigue, pain, neuro-cognitive difficulties, mood disturbance, overall illness severity. Apply endophenotype concept to clinical dataset describing symptom domains of acute sickness response post viral/non-viral pathogens, and validation by showing association with SNP in cytokine genes (IL-6, TNFa, IFNy, IL-10) | SPHERE (and SOMA), PSC, BDQ, principal component analysis, NanoDropR ND-1000 (DNA quantification), Sequenom MassARRAY® (genotyping of SNP) | NA | Individual symptom indices correlated with overall severity and functional status. Domain scores stable over time within subjects, but varied between subjects with same infection, and across infection sub-cohorts. Overall illness severity may have been comparable in some subjects, relative contributions from individual symptom domains making up the illness complex varied between these subjects. T allele IFNy+874T/A SNP best predictor of ↑ fatigue. ♀ more likely grouped in ↑ fatigue extreme. C allele of IL-10-592C/A SNP exerted protective effect on neurocognitive difficulties. A allele IL-10-592 SNP and G allele IL-6-174G/C SNP associated ↑ mood disturbance | Acute illness response has discrete symptoms including fatigue with unique genetic associations. Study offers new pathophysiological inside fatigue states. Illness severity phenotype not dependent on age/sex/infection subtype. Robust correlation between illness severity and reported disability in AI. ♀ over represented in high severity group fatigue, mood disturbance, neurocognitive difficulties | NA | ★  ★  ★ | ★  ★ |  |
| 2012, H. Hussain-Yusuf [17] | UK, 2008 | CC | Cohort 211 UK factory workers *C.b.*-exposed 2002. FU 6 yrs post outbreak, comparison QF serology, presence viable *C.b.*, its DNA and fatigue in post AQF cases (n=38, 3 uncertain serology 2002) vs. seronegative, same outbreak (n=14). Assess if *C.b.* antigens (immunomodulatory complex) remain undegraded in some post AQF, with abnormal cytokine profile causing ongoing fatigue | Chalder Fatigue Scale, qPCR (com1 gene) on PBMC and VERO cultures (detect *C.b.* DNA), IFA, SCID mice inocula-tion (detect viable *C.b.*) | NA | 18% became seronegative, remainder 10 phase I, 21 phase I en II antibodies. 29% controls became seropositive. No patient/control PBMC contained viable *C.b.*/DNA. No viable *C.b.* in PMBC tested in cell culture and SCID mice inoculation. Chalder Fatigue Scale score after 6 yrs (n=11): 4 significant fatigue, 4 some, 3 not fatigued. No relationship between fatigue levels and serology, nor with presence of viable *C.b.*/DNA | 6 yrs post AQF, some patients became seronegative but none contained viable *C.b.*/DNA in their PBMC. Correlation PQFF and persistent DNA could not be examined. A more sensitive DNA assays or more invasive sampling needed to test hypothesis. IgGII most useful to test past QF exposure | B/D | ★  ★  ★  ★ |  | ★  ★ |
| 2014, M. Kremers [18] | Netherlands, yr study 2013-2014. Study period: April- August 2009, FU 4 yrs post AQF | CoS | 102 seronegative PCR positive, symptomatic, AQF patients (64.7% ♂, mean age 48, SD16, range 17-85); 24 hospitalised. 93 FU 3, 6 or 12 mo for IFA IgGI and II. NCSI 4 yrs post AQF (n=58). Assess if ↑ CRP AQF coincides with ↑ IL-6 and if levels correlate with *C.b.* DNA load and disease severity, expressed by hospital admission and fatigue development | NCSI, PCR (Ct value), IFA, CRP, IL-6 | NA | 92 patients ↑ IL-6, 101 ↑ CRP during AQF. Significant weak negative correlation *C.b.* DNA loads, IL-6 and CRP, significant moderate-strong positive correlation IL-6 and CRP. Hospitalised patients: ↑ IL-6 and CRP than the non-hospitalised, *C.b.* DNA load equal. NCSI: 58 respondents, 34 abnormal outcome (58.6%) mild and severe fatigue. No difference in Ct values, CRP and IL-6 in AQF between patients with normal outcome and abnormal outcome subdomain fatigue | Correlation IL-6 and CRP in AQF points to immune activation pathway in which IL-6 induces CRP. Differences IL-6 and CRP between hospitalised vs. the non-hospitalised despite identical DNA load suggest an important role for host factors. ↑ IL-6 and CRP seems predictive of more severe disease. No support that IL-6 or CRP levels during AQF are prognostic for fatigue development | NA | ★  ✰★ |  | ★  ★ |

**** Definition of used study population in articles explained in a different table, including definitions of QFS and/or fatigue is applicable. Main information is on aetiology. Some articles also contain relevant information on other domains: Diag= Diagnosis, B/D= Background/descriptive, P/T= Prevention/therapy***

***Abbreviations:*** 2-5AS= 2',5'-oligoadenylate synthetase, AI= Acute infection, AQF= Acute Q-fever, BDQ= Brief Disability Questionnaire, assessment of the impact of illness on functional capacity, and days out of role quantified the days over the past months the respondent was unable to carry out usual daily activities fully, BMA= Bone marrow aspirate, BMI= Body Mass Index, *C.b.=* *Coxiella burnetii,* CBA= Cytometric bead array, uses the sensitivity of amplified fluorescence detection by flow cytometry to measure soluble analytes (e.g. interleukins) in a particle-based immunoassay, CC= Case-control study, CDC= Centres for Disease Control and Prevention, CF= Chronic fatigue, CFS(/ME)= Chronic fatigue syndrome (/myeloencephalitis), CFT= Complement fixation test, CIDI= Composite international diagnostic interview to screen for any history of depression, anxiety or somatisation disorder. This computerised program formulates ICD-10 and DSM-III-R diagnoses and records current as well as pre-existing psychiatric morbidity, CoS= Cohort study, CRP= C-reactive protein, CS= Cross-sectional, DIOS= Dubbo Infection Outcomes Study**,** cohort study of subjects ≥16 yrs followed from the onset of a confirmed and documented AI due to EBV; *C.b.*; or RRV ≤6 wks post AI until complete recovery, DS14= Distressed personality scale, assessment of negative affectivity (an enduring tendency to experience negative emotions) and trait social inhibition (the tendency to feel inhibited, tense, and insecure when with others), DTH= Delayed-type hypersensitivity, to assess cell-mediated immune function in vivo, EBV= *Epstein-Barr virus*, ECG= Electrocardiography, FU= Follow-up, GHQ= General health questionnaire, 12-item questionnaire to detect current cases of psychiatric co-morbidity, I.c.w.= In comparison with, IFA= Immunofluorescence assay, IFN= Interferon, IgG= Anti-phase IgG, IgGI= Anti-phase IgG I titre, IgGII= Anti-phase IgG II titre, IL= Interleukin, IS= Insertion sequence, K10= Kessler 10, to assess current psychological distress, LMR= Lymphocyte mitogenic responses, LPS= Lipopolysaccharide, Mo= Month(s), MUGA scan= Multi Gated Acquisition Scan (gated cardiac radio-nuclide scans), a time-proven nuclear medicine test to evaluate the function of the right and left ventricles of the heart, allowing informed diagnostic intervention in heart failure, NA= Not applicable, NCSI= Nijmegen clinical screening instrument, originally developed to provide a detailed assessment of health status of COPD patients. It combines a number of existing health status questionnaires, NOS= Newcastle–Ottawa Scale: S= selection (maximum of 4 stars), C= comparability (maximum of 2 stars), O= outcome (maximum of 3 stars); ★: star earned; ☆: item not applicable, N/No= Number (of), (n-)PCR= (nested-) Polymerase chain reaction, NR= Not reported, Pain test algometer= For pressure pain threshold test to measure pain sensitivity, PBMC= Peripheral blood mononuclear cells, PHA= phytohaemagglutinin, PIF(S)= Post-infective fatigue (syndrome), PO= Personal opinion, POMS= Profile of Mood States to assess current mood status. This instrument includes 7 subscales: ‘fatigue’, ‘depression’, ‘anxiety’, ‘vigour’, ‘anger’, ‘friendliness’, and ‘confusion’, PQFF= Post-Q-fever fatigue, PQF(F)S= Post-(acute)Q-fever (fatigue) syndrome, Pros.= Prospective, PSC= Physical Symptoms Checklist, consisting of 51 symptom items, PSQ= Pittsburgh Sleep Questionnaire, to assess sleep abnormalities, QA = Quality assessment, Q-CFS(/ME)= Q-fever induced chronic fatigue syndrome (/myeloencephalitis), QF= Q-fever, QF(F)S= Q-fever fatigue syndrome, (Q)IE= (Q-fever induced) Infective endocarditis, Ref= Reference, RRV= *Ross River virus*, SCID= Severe combined immunodeficiency, SD= Standard deviation, SF-36= The Short Form (36) Health Survey, a patient-reported survey of patient health to assess quality of life of patients, functional impairment and reduced health related quality of life, SNP= Single nucleotide polymorphism, SOFA= Schedule of Fatigue and Anergy to identify cases of chronic fatigue syndrome. The subject rates 10 items on a 4-point scale. Subjects who score ≥3 items as ‘a good part of the time’ or ‘most of the time’ are classified as cases of ‘fatigue/neurasthenia’, SOMA= Empirically derived subscale of the SPHERE, used to record PIFS or illness duration. This reliably predicts disability and reflects patients’ and doctors’ reports of reasons for presentation to primary care. Scores ≥3 represents a clinically-significant fatigue state. Provisional PIFS: SOMA scores ≥3 at all time points up ≤3 months. Confirmed PIFS: symptoms persisted >6 months, and alternative explanations for ongoing illness was excluded. SPHERE= Somatic and Psychological Health Report, to assess a wide range of physical and psychological symptoms, including severity and duration of symptoms, Stroop task= To assess cardiac response, TGFB= Transforming growth factor beta, TNF= Tumor necrosis factor, UK= United Kingdom, Wks= Weeks, Yr(s)= Year(s)

**References**

1. Bennett BK, Hickie IB, Vollmer-Conna US, Quigley B, Brennan CM, Wakefield D, et al. The relationship between fatigue, psychological and immunological variables in acute infectious illness. Aust N Z J Psychiatry. 1998;32(2):180-6. Epub 1998/05/20. PubMed PMID: 9588296.

2. Penttila IA, Harris RJ, Storm P, Haynes D, Worswick DA, Marmion BP. Cytokine dysregulation in the post-Q-fever fatigue syndrome. QJM. 1998;91(8):549-60. Epub 1999/01/20. PubMed PMID: 9893758.

3. Harris RJ, Storm PA, Lloyd A, Arens M, Marmion BP. Long-term persistence of Coxiella burnetii in the host after primary Q fever. Epidemiol Infect. 2000;124(3):543-9. Epub 2000/09/12. PubMed PMID: 10982079; PubMed Central PMCID: PMCPmc2810941.

4. Ayres JG, Wildman M, Groves J, Ment J, Smith EG, Beattie JM. Long-term follow-up of patients from the 1989 Q fever outbreak: no evidence of excess cardiac disease in those with fatigue. QJM. 2002;95(8):539-46. Epub 2002/07/30. PubMed PMID: 12145393.

5. Raoult D. Q fever: still a mysterious disease. QJM. 2002;95(8):491-2. Epub 2002/07/30. PubMed PMID: 12145387.

6. Helbig KJ, Heatley SL, Harris RJ, Mullighan CG, Bardy PG, Marmion BP. Variation in immune response genes and chronic Q fever. Concepts: preliminary test with post-Q fever fatigue syndrome. Genes Immun. 2003;4(1):82-5. Epub 2003/02/22. doi: 10.1038/sj.gene.6363912. PubMed PMID: 12595908.

7. Ikuta K, Yamada T, Shimomura T, Kuratsune H, Kawahara R, Ikawa S, et al. Diagnostic evaluation of 2', 5'-oligoadenylate synthetase activities and antibodies against Epstein-Barr virus and Coxiella burnetii in patients with chronic fatigue syndrome in Japan. Microbes Infect. 2003;5(12):1096-102. Epub 2003/10/14. PubMed PMID: 14554250.

8. Marmion BP, Storm PA, Ayres JG, Semendric L, Mathews L, Winslow W, et al. Long-term persistence of Coxiella burnetii after acute primary Q fever. QJM. 2005;98(1):7-20. Epub 2004/12/31. doi: 10.1093/qjmed/hci009. PubMed PMID: 15625349.

9. Helbig K, Harris R, Ayres J, Dunckley H, Lloyd A, Robson J, et al. Immune response genes in the post-Q-fever fatigue syndrome, Q fever endocarditis and uncomplicated acute primary Q fever. QJM. 2005;98(8):565-74. Epub 2005/06/16. doi: 10.1093/qjmed/hci086. PubMed PMID: 15955794.

10. Vollmer-Conna U, Cameron B, Hadzi-Pavlovic D, Singletary K, Davenport T, Vernon S, et al. Postinfective fatigue syndrome is not associated with altered cytokine production. Clin Infect Dis. 2007;45(6):732-5. doi: <http://dx.doi.org/10.1086/520990>. PubMed PMID: 2007442464.

11. Marmion BP, Sukocheva O, Storm PA, Lockhart M, Turra M, Kok T, et al. Q fever: persistence of antigenic non-viable cell residues of Coxiella burnetii in the host--implications for post Q fever infection fatigue syndrome and other chronic sequelae. QJM. 2009;102(10):673-84. Epub 2009/06/27. doi: 10.1093/qjmed/hcp077. PubMed PMID: 19556396.

12. Zhang L, Gough J, Christmas D, Mattey DL, Richards SC, Main J, et al. Microbial infections in eight genomic subtypes of chronic fatigue syndrome/myalgic encephalomyelitis. J Clin Pathol. 2010;63(2):156-64. Epub 2009/12/04. doi: 10.1136/jcp.2009.072561. PubMed PMID: 19955554; PubMed Central PMCID: PMCPmc2921262.

13. Kadota Y, Cooper G, Burton AR, Lemon J, Schall U, Lloyd A, et al. Autonomic hyper-vigilance in post-infective fatigue syndrome. Biol Psychol. 2010;85(1):97-103. doi: <http://dx.doi.org/10.1016/j.biopsycho.2010.05.009>. PubMed PMID: 2010421837.

14. Sukocheva OA, Marmion BP, Storm PA, Lockhart M, Turra M, Graves S. Long-term persistence after acute Q fever of non-infective Coxiella burnetii cell components, including antigens. QJM. 2010;103(11):847-63. Epub 2010/07/20. doi: 10.1093/qjmed/hcq113. PubMed PMID: 20639288.

15. Galbraith S, Cameron B, Li H, Lau D, Vollmer-Conna U, Lloyd AR. Peripheral blood gene expression in postinfective fatigue syndrome following from three different triggering infections. J Infect Dis. 2011;204(10):1632-40. Epub 2011/10/04. doi: 10.1093/infdis/jir612. PubMed PMID: 21964398.

16. Piraino B, Vollmer-Conna U, Lloyd AR. Genetic associations of fatigue and other symptom domains of the acute sickness response to infection. Brain Behav Immun. 2012;26(4):552-8. Epub 2012/01/10. doi: 10.1016/j.bbi.2011.12.009. PubMed PMID: 22227623.

17. Hussain-Yusuf H, Islam A, Healy B, Lockhart M, Nguyen C, Sukocheva O, et al. An analysis of Q fever patients 6 years after an outbreak in Newport, Wales, UK. QJM. 2012;105(11):1067-73. Epub 2012/07/10. doi: 10.1093/qjmed/hcs119. PubMed PMID: 22771556.

18. Kremers MN, Janssen R, Wielders CC, Kampschreur LM, Schneeberger PM, Netten PM, et al. Correlations between peripheral blood coxiella burnetii DNA load, interleukin-6 levels, and C-reactive protein levels in patients with acute Q fever. Clin Vaccine Immunol. 2014;21(4):484-7. doi: <http://dx.doi.org/10.1128/CVI.00715-13>. PubMed PMID: 2014239787.
