# Supplementary material for: Fatigue following Acute Q-Fever: A Systematic Literature Review
Source: PLoS One. 2016 May 25;11(5):e0155884. doi: 10.1371/journal.pone.0155884 (PMC4880326; doi:10.1371/journal.pone.0155884)
Supplement: S5 Table — (DOCX) [file pone.0155884.s006.docx]

**S5 Table. Domain prevention/therapy**

| **Ref** | **Country, yr study, period and duration** | **Study type** | **Patients, controls, characteristics, co-morbidity*** | **Tool** | **Intervention** | **Outcome** | **Conclusions/**  **recommendations** | **Other do-main** | **QA (CR or NOS)** | | |
| --- | --- | --- | --- | --- | --- | --- | --- | --- | --- | --- | --- |
| 2004, Y. Arashima [1] | Japan, yr study NR. Period: Jul-Nov 2001, baseline, 4, 8 and 12 wks post start treatment | CoS | 20 QFS patients (3 ♂, mean age 34.6±5.7) with subjective symptoms (duration 20.8±3.3 mo, range 3 mo-4 yrs): fatigue (20/20), slightly elevated body temperature (17/20), arthralgia or myalgia (10/20), headache (12/20), cough or sore throat (16/20), ↑ sweating (10/20), and gastrointestinal symptoms (13/20). To address presence post QFS in Japan, and evaluation of minocycline for post QFS in changes in subjective symptoms, *C.b.* antibody titres and *C.b.* DNA. No controls | Questionnaires (assess severity of subjective symptoms), PS score, IFA, n-PCR. Antibiotic side effects evaluated by interview and laboratory examination results | 3 mo: minocycline 100mg/d (n=18)/ erythromycin 400mg/d (n=1)/ levofloxacin 200mg/d (n=1) | No leucocytosis or ↑ ESR. Slightly ↑ CRP 5 patients. All 7 who had been DNA positive, became negative with improvement subjective symptoms. IgM and IgG antibodies became negative post treatment. Clinical picture all patients improved: general fatigue (20/20), ↓ body temperature (12/17), gastrointestinal symptoms (10/13) and headache (9/12). PS score related to fatigue unchanged in 2 mo, but finally ↓, PS scores ↑ | Minocycline administration useful for improving chronic nonspecific symptoms considered to be post QFS, and should be first-line drug for QFS. Observations may reflect existence of live *C.b.* in QFS patients | Diag, B/D | ✰★  ✰ | ✰  ✰ | ★  ★★ |
| 2005, E. Iwakami [2] | Japan, May 2001-March 2003. Period: baseline, 3 mo treatment | CoS | 4/8 CFS patients (2 ♂; 1 with IgGII 1:128; 3 with *C.b.* DNA positive); mean age 29, SD4, range 23-33, duration complaints: 52.0 mo, SD55.3, range 8 mo-11 yrs. Fatigue (PS score 7±1.2), slightly elevated body temperature, headache, arthralgia/myalgia (100%), cough/sore throat (75%). 54 QFS patients (10 ♂) positive *C.b.* DNA (n=34), IgMII ≥1:32 (n=15)/IgGII ≥1:128 (n=34); mean age 38, SD16, range 11-77, duration complaints: 21.1 mo, SD24.3, range 1 mo-10 yrs. Fatigue (PS score 5.3±2.4), slightly elevated body temperature (100%), headache (63%). To explore *C.b.* in CFS by antibiotic treatment, monitor symptom changes, PCR and *C.b.* antibodies | n-PCR, *C.b.* antibodies initial examination and 3 mo after start treatment. Questionnaire survey, PS | 3 mo: minocycline 100mg/d (n=29)/ doxycycline 100mg/d (n=26)/levofloxacin 200mg/d (n=3) | All 58 patients tested *C.b.* after treatment; all n-PCR positives became negative. CFS group (n=4): no improvement PS (p=0.422), no difference pre- and post-treatment temperatures (p=0.07) or headache (p=0.39) scores. QFS group: PS scores improved (p<0.001), temperature (p<0.001) and headache scores ↓ (p<0.001) post treatment | Possibility direct involvement *C.b.* pathological state CFS low. Different response to tetracycline suggest direct *C.b.* involvement pathological state QFS. Latent *C.b.* infection not involved either onset CFS or appearance symptoms | Diag, B/D | ★  ★ |  | ★  ★ |
| 2007, D. Ledina [3] | Croatia, yr study NR, study period: 2000-2004 | Case-  series | N=3 post AQF with PQFS. 2 ♂ (34 and 30 yrs), 1 ♀ (30 yrs). Initial treatment AQF: erythromycin and gentamycine 2 wks (n=1), doxycycline 2 wks (n=2). Emphasize existence and incidence CFS post AQF according to CDC CFS criteria, and show effects antibiotic treatment in QFS | Questionnaires before and after treatment for subjective symptoms. Noted in 4 degrees absent-severe | Case 1: 9 mo doxycycline 200mg/d + ciprofloxacin 1000mg/d. Case 2: ciprofloxacin 1000mg/d 2 mo, then doxycycline 200mg/d 4 mo. Case 3: 1 mo corticosteroids, then 3 mo doxycycline | Case 1: still fatigue after physical activity (disappears after 30 min rest) and low intensity headache. Muscle pain and slightly elevated body temperature disappeared. No criteria CFS post-treatment. Case 2: regression symptoms, except minor headache. No criteria CFS post-treatment. Case 3: still fatigued, disrupted sleep, headache, muscle and joint pains, still fulfils CFS criteria post treatment | Results prolonged antibiotic treatment CFS inconsistent. Diagnostic criteria and therapeutic recommendations for PQFS require further investigation | Diag, B/D | 9/18 criteria ** | | |
| 2013, S. Keijmel [4] | Netherlands, yr study: 2011-2015 | RCT proto-col | Objective: include 180 QFS patients, ♂ and ♀. Evaluation of efficacy of long-term doxycycline and CBT in QFS-patients | CIS; SIP total score, total score SCL-90, *C.b.* PCR and serology | 24 wks of: placebo, doxycycline 200 mg/d, or CBT | Still treating patients | NA | Diag | NA | | |
| 2013, S. Yakubo [5] | Japan, yr study NR | CR | ♀ 71 yrs, 6 yrs post AI with general malaise, spasm left hand, slightly elevated body temperature. Co-morbidity: NR. Negative n-PCR for *C.b.*, IgMI and IgMII <1:16, IgGI <1:16, IgGII 1:32 | n-PCR, IFA | Kampo formula Tsumura Shakuyaku-Kanzo-To granules (7,5g/d) 3 mo | Alleviation of stiffness in hand and arm after 2 days treatment, symptom disappeared completely. 6 mo after start treatment reappearance stiffness and IgGI 1:128 | QFS may feature intermittent muscle spasms, ameliora-ted by Shakuyaku-Kanzo-To granules, warrants further research | NA | -/-, -,  +, +, +, NA, -/-, -/- | | |
| 2013, S. Yakubo [6] | Japan, yr study NR | CR | ♂ 13 yrs, fatigue and severe malaise, slightly elevated body temperature, arthralgia, myalgia, lassitude, disease period 2 mo earlier. Extended period no school attendance. Co-morbidity: NR. IFA IgMII and IgGII negative, n-PCR positive | n-PCR, IFA | Kampo formula Tsumura Hochu-ekki-To granules (7,5mg/d) 1 mo, then erythromycin 800mg/d 1 mo, then doxycycline 200mg/d 1 mo, then erythromycin 800mg/d at least 6 mo | Slight improvement 1 mo post erythromycin, none post doxycycline, fever stopped after long-term erythromycin, general malaise continued. Improvement after continued treatment | Consider *C.b.* as possible cause in cases of long-term school absence due to severe malaise similar to that caused by CFS | Diag, B/D | -/-, +,  -/-, +/-, +/-, NA, +/-, -/- | | |

**** Definition of used study population in articles explained in a different table, including definitions of QFS and/or fatigue is applicable. Main information is on prevention/therapy. Some articles also contain relevant information on other domains: Diag= Diagnosis, B/D= Background/descriptive, A= Aetiology***

***** Quality assessment for case-series was performed with a quality appraisal tool making use of 18 criteria with a considered acceptable quality if at least 14 criteria were scored (≥70%) [7]***

***Abbreviations:*** AI= Acute infection, AQF= Acute Q-fever, *C.b.=* *Coxiella burnetii,* CBT= Cognitive behavioural therapy, CDC= Centre of Disease Control, CFS= Chronic fatigue syndrome, CIS= subscale fatigue of the Checklist Individual Strength, to indicate the level of fatigue experienced in the previous two weeks, measured with eight items on a seven-point Likert-scale (range 8–56), CoS= Cohort study, CRP= C-reactive protein, CR= Case-report, ESR= Erythrocyte sedimentation rate, IFA= Immunofluorescence assay, IgG= Anti-phase IgG, IgGII= Anti-phase IgG II titre, IgM= Anti-phase IgM, IgMII= Anti-phase IgM II titre, Mo= Month(s), NA= Not applicable, NOS= Newcastle–Ottawa Scale: S= selection (maximum of 4 stars), C= comparability (maximum of 2 stars), O= outcome (maximum of 3 stars); ★: star earned; ☆: item not applicable, N/No= Number (of), (n-)PCR= (nested-) Polymerase chain reaction, NR= Not reported, PQFS= Post-(acute)Q-fever (fatigue) syndrome, PS= Performance status score (range 0-9), which reflects the grade of fatigue/malaise to assess the severity of CFS, QA-CR= Quality assessment; for CR no quality checklists are available. Therefore, the following eight criteria for quality assessment were determined; addressing an appropriate and clearly focused question, representative population, description of the survey method or data collection, outcome measures defined, outcome measures described, response rate reported and results valid and applicable to the patient group targeted. The articles scores on these items: -/-, -, +/-, +, or ++, based on the Coordination of Cancer Clinical Practice Guidelines in Europe criteria, RCT= Randomised controlled trial, QF(F)S= Q-fever fatigue syndrome, Ref= Reference, SCL-90= Symptom Checklist 90, to measure the level of psychological distress, consisting of 90 items scored on a five-point Likert-scale (range 90-450), SD= Standard deviation, SIP= Sickness Impact Profile, to measure the level of functional impairment. A total score is derived out of the scores on the subscales: sleep-rest, household, mobility, social interactions, walking, alertness and intellectual functioning, work, and recreation, Wks= Weeks, Yr(s)= Year(s)

**References**

1. Arashima Y, Kato K, Komiya T, Kumasaka K, Matsukawa Y, Murakami M, et al. Improvement of chronic nonspecific symptoms by long-term minocycline treatment in Japanese patients with Coxiella burnetii infection considered to have post-Q fever fatigue syndrome. Intern Med. 2004;43(1):49-54. Epub 2004/02/18. PubMed PMID: 14964579.

2. Iwakami E, Arashima Y, Kato K, Komiya T, Matsukawa Y, Ikeda T, et al. Treatment of chronic fatigue syndrome with antibiotics: pilot study assessing the involvement of Coxiella burnetii infection. Intern Med. 2005;44(12):1258-63. Epub 2006/01/18. PubMed PMID: 16415546.

3. Ledina D, Bradaric N, Milas I, Ivic I, Brncic N, Kuzmicic N. Chronic fatigue syndrome after Q fever. Med Sci Monit. 2007;13(7):Cs88-92. Epub 2007/06/30. PubMed PMID: 17599032.

4. Keijmel SP, Delsing CE, Sprong T, Bleijenberg G, van der Meer JW, Knoop H, et al. The Qure study: Q fever fatigue syndrome--response to treatment; a randomized placebo-controlled trial. BMC Infect Dis. 2013;13:157. Epub 2013/03/30. doi: 10.1186/1471-2334-13-157. PubMed PMID: 23536997; PubMed Central PMCID: PMCPmc3620935.

5. Yakubo S, Yakubo S, Ueda Y, Tanekura N, Arashima Y, Munemura T, et al. Kampo Formula Shakuyaku-kanzo-To Alleviates Sensation of Muscle Spasm in Coxiella burnetii Infection. International Medical Journal. 2013;20(2):218-20. PubMed PMID: 2012154829.

6. Yakubo S, Ueda Y, Arashima Y. Long-term absence from school of a boy suffering severe general Malaise from coxiella burnetii infection. International Medical Journal. 2013;20(6):688-90. PubMed PMID: 2014054222.

7. Moga C, Guo B, Schopflocher D, Harstall C. Development of a quality appraisal tool for case series studies using a modified Delphi technique; 2012.
