# Supplementary material for: Fatigue following Acute Q-Fever: A Systematic Literature Review
Source: PLoS One. 2016 May 25;11(5):e0155884. doi: 10.1371/journal.pone.0155884 (PMC4880326; doi:10.1371/journal.pone.0155884)
Supplement: S6 Table — (DOCX) [file pone.0155884.s007.docx]

**S6 Table. Grey literature**

| **Ref** | **Country, yr study, period and duration** | **Docu-ment** | **Patients, controls, characteristics, co-morbidity** | **Tool** | **Outcome/advice** | **Conclusions/recommendations** | **Do-mains** | **QA** |
| --- | --- | --- | --- | --- | --- | --- | --- | --- |
| 1992, M. Shannon [1] | Australia, yr study NR, study period NR | Thesis | Abattoir workers (n=117), immune status assessed 1981-1986. Group of clinical history AQF and serology CFT Phase I and II, and IFA (n=39). All either ↑ CFT antibody titre and/raised IFA IgM as indication current QF. Unexposed comparison cohort (n=39): vaccinated and non-vaccinated (seropositives without clinical history AQF). Occurrence infection not noted | *C.b.* CFT, IFA, questionnaires | Definition QFS; laboratory proven, clinically manifest QF, commences within 12 mo of illness, duration ≥6 mo. 5 major symptoms; 1. fatigue of 2-≥7dys, ≥6x/yr continuously with some absence from work, 2. malaise – as above except work, 3. muscle twitches/ fasciculations, 4. nausea ≥6x/yr, 5. abnormal sweating ≥10x/yr, might be accompanied by other symptoms. Most subjects healthy before AQF regarding depression. Mental problems; depression, lack of concentration, impairment short memory, mood lability, altered sleep pattern following AQF. Some general practitioners stated that tricyclic antidepressants were beneficial. 30-40 cases/1000 abattoir workers/yr, each costs 2-88.000 in medical care and loss of wages, endocarditis 50-10.000/yr, QFS 20-50.000/yr. Duration QFS 6 mo-20 yrs | Approximately 23% develops QFS post overt AQF. No grounds to dismiss QFS as a psychiatric depressive illness. Aetiology is unclear, might be due to immune stimulation and a disordered function of the lymphocyte-macrophage interaction. Same pathways to mood change may be involved in depression and QFS and altered by chemotherapy | **B/D**,  P/T | NA |
| 2009, B. Marmion [2] | Australia and UK, yr study NR, study period NR | Book (chap-ter) | No patients/controls. Characteristics and co-morbidity: NR. Experience from several studies | NA | Start often 6 mo-1 yr post AQF. Symptoms complex not limited to fatigue, also nausea, headache, night sweats, myalgia, arthralgia, fasciculations, painful lymph nodes, disturbed sleep pattern, anger, ↓ concentration, mental acuity ↓. Duration: >1 yr, often 5-10 yrs. Antigens in samples SCID mice, cellular immune response heightened, cytokine dysregulation: IL-6 ↑, IL-10, IL-2 ↓, low fever. Pathogenesis; no consensus. Bacteraemia restricted by humoral and cell-mediated immunity, by product clearing *C.b.* DNA containing components with an immunomodulatory effect. Cell-mediated immunity and dendritic cells causing dysregulation, cytokines and other immune mediators give rise to symptoms | In Australia QFS is the most common chronic sequel of AQF affecting 10-15% of patients. It usually follows AQF and rarely if ever subclinical infection | **B/D**,  A | NA |
| 2011, C. Tempel-man [3] | Netherlands, yr study 2011 | Report on eco-nomic evalua-tion | Economic costs –human and veterinary Dutch QF outbreak 2007-2010 assessed with 4024 notification AQF. Assumptions: 25% (n=503) AQF get QFS duration 5-10 yrs. Results: quality of life ↓, assumed period sick leave 5-10 yrs, productivity 50% ↓. Assumption 60% of those with QFS were gainfully employed | Interviews, public data outbreak | QFS duration 5-10 yrs costs ↓ quality of life 55.6-104.7 million euros. Costs of sick-leave due to QFS are not separately presented but together with CQF and therefore not mentioned | Economic costs due to QF outbreak are considerable as the course of disease especially due to QFS is protracted and reflected in ↓ quality of life, ↓ productivity, and ↓ income | **B/D** | NA |
| 2012 Guideline working group on QFS [4] | Netherlands, yr study 2011-2012 | Guideline | Achieve uniformity diagnosis and treatment QFS | QFS and CFS literature and multidiscipline-ry consensus | QFS definition: severe fatigue causing significant disabilities daily life ≥6 mo, reference to lab confirmed AQF, not caused by somatic/psychiatric co-morbidity, fatigue absent before AQF/significantly ↑ since. Diagnosis on history, physical and laboratory examination excluding other causes of fatigue (including ESR, CRP, CK, TSH, leukocytes with differentiation, creatinine, alkaline phosphatase, ALT, glucose, ferritin, urinary sediment). Cave diagnosis in case of morbid obesity (BMI>40) or substance abuse. Impossible to diagnose QFS in case of: depression/depression preceded current symptoms, schizophrenia, psychoses, any type dementia, eating disorders, unless resolved ≥5 yrs | Advice patients ≤6 mo post AQF: i) stay mentally/physically active, adjust pace if necessary; ii) alternate activities, also within activities; iii) keep fulfilling daily role; iv) keep steady sleep-wake pattern; v) avoid focussing on fatigue; vi) focus on feasible activities, appreciate accomplishments. Advice CBT/GET after QFS diagnosis. GET might be an additional treatment strategy | **Diag**, B/D, A, P/T | NA |

***These documents contain relevant information for the domains: Diag= Diagnosis, B/D= Background/descriptive, A= Aetiology, P/T= Prevention/therapy. Main domain indicated in bolt***

***Abbreviations:*** ALT= Alanin aminotransferase, AQF= Acute Q-fever, BMI= body mass index, *C.b.=* *Coxiella burnetii,* CBT= Cognitive behavioural therapy, CFS= Chronic fatigue syndrome, CFT= complement fixation test, CK= creatine kinase, CRP= C-reactive protein, CQF= chronic Q-fever, ESR= Erythrocyte sedimentation rate, IFA= Immunofluorescence assay, IL= Interleukin, Mo= Month(s), NA= Not applicable, NR= Not reported, QF= Q-fever, QF(F)S= Q-fever fatigue syndrome, Ref= Reference, TSH= thyroid stimulating hormone, Yr(s)= Year(s)

**References**

1. Shannon M. The post Q fever fatigue syndrome: an epidemiological study (dissertation). Adelaide: University of Adelaide; 1992.

2. Marmion B. A guide to Q fever and Q fever vaccination. Australia: CSL Biotherapies; 2009. 125 p.

3. Tempelmann C, Prins J, Koopmans C. Economical consequences of the Q fever outbreak [in Dutch], SEO Econ. Res. (2011) 2011-2015.

4. National Institute for Public Health and the Environment. Dutch guideline Q fever fatigue syndrome (QFS) [in Dutch] 2012. Available from: <http://www.rivm.nl/dsresource?objectid=rivmp:118226&type=org&disposition=inline>.
